# T2 and T17 cytokines alter the cargo and function of airway epithelium-derived extracellular vesicles

**DOI:** 10.1186/s12931-020-01402-3

**Published:** 2020-06-19

**Authors:** Elisabeth Ax, Zala Jevnikar, Aleksander Cvjetkovic, Carina Malmhäll, Henric Olsson, Madeleine Rådinger, Cecilia Lässer

**Affiliations:** 1 Krefting Research Centre, Institute of Medicine at the Sahlgrenska Academy, University of Gothenburg, Gothenburg, Sweden; 2 Translational Science and Experimental Medicine, Research and Early Development, Respiratory & Immunology, BioPharmaceuticals R&D, AstraZeneca, Gothenburg, Sweden

**Keywords:** Asthma, Exosomes, Mediators of inflammation, Proteomics, Respiratory epithelium

## Abstract

**Background:**

Asthma is a common and heterogeneous disease that includes subgroups characterized by type 2 (T2) or type 17 (T17) immune responses for which there is a need to identify the underlying mechanisms and biomarkers in order to develop specific therapies. These subgroups can be defined by airway epithelium gene signatures and the airway epithelium has also been implicated to play a significant role in asthma pathology. Extracellular vesicles (EVs) carry functional biomolecules and participate in cell-to-cell communication in both health and disease, properties that are likely to be involved in airway diseases such as asthma. The aim of this study was to identify stimulus-specific proteins and functionality of bronchial epithelium-derived EVs following stimulation with T2 or T17 cytokines.

**Methods:**

EVs from cytokine-stimulated (T2: IL-4 + IL-13 or T17: IL-17A + TNFα) human bronchial epithelial cells cultured at air-liquid interface (HBEC-ALI) were isolated by density cushion centrifugation and size exclusion chromatography and characterized with Western blotting and electron microscopy. Transcriptomic (cells) and proteomic (EVs) profiling was also performed.

**Results:**

Our data shows that EVs are secreted and can be isolated from the apical side of HBEC-ALI and that cytokine stimulation increases EV release. Genes upregulated in cells stimulated with T2 or T17 cytokines were increased also on protein level in the EVs. Proteins found in T17-derived EVs were suggested to be involved in pathways related to neutrophil movement which was supported by assessing neutrophil chemotaxis ex vivo.

**Conclusions:**

Together, the results suggest that epithelial EVs are involved in airway inflammation and that the EV proteome may be used for discovery of disease-specific mechanisms and signatures which may enable a precision medicine approach to the treatment of asthma.

## Background

Asthma is a common airway disease that is heterogeneous with variations in severity, clinical phenotypes and underlying molecular mechanisms (endotypes) [1, 2]. The most common and studied asthma phenotype is type 2 (T2) asthma characterized by type 2 cytokines interleukin (IL)-4, − 5 and − 13, whereas different immune cells and mediators drive the inflammation in the less studied type 17 (T17) asthma [3]. The majority of people with T2 asthma respond well to standard treatment with inhaled corticosteroids whereas individuals with T17 asthma are usually less responsive [2, 4, 5]. Non-invasive monitoring of local immune mechanisms in asthma remains challenging, but there is a need to identify and better understand such mechanisms in order to develop improved treatment of asthma patient subgroups [6, 7].

The airway epithelium acts as the first line of defense against inhaled agents by forming a physical barrier and by bridging innate and adaptive immune responses [8]. Previous studies have demonstrated the role of the airway epithelium in inflammatory respiratory diseases, and it has been suggested to use epithelial gene and protein signatures for patient stratification [9–12].

Extracellular vesicles (EVs) are nano-sized cell-derived vesicles that can be found in body fluids such as blood, saliva and bronchoalveolar lavage fluid (BALF) [13]. EVs consist of several subpopulations of vesicles, with exosomes and microvesicles being the best defined. They commonly contain message molecules and provide a means for donor cells to alter the phenotype of recipient cells [14, 15]. Furthermore, the EV cargo is representative of its origin, and disease markers, such as proteins and microRNAs, have been found in body fluid-derived EVs from for example cancer patients [16, 17]. However, less is known about EVs in inflammatory airway diseases, but differences in cargo and properties have been demonstrated for EVs isolated from BALF from asthmatics and healthy controls [18, 19]. The lung epithelium has been shown to be a major source of vesicles in the airways, suggesting that epithelial EVs may play an important role in the pathology of asthma [18, 20–22].

The aim of this study was to determine how protein composition and function of bronchial epithelium-derived EVs are altered during disease-relevant inflammatory conditions in vitro, and whether EVs may mediate endotype-specific mechanisms related to asthma.

## Methods

### Cell culture and stimulation

Primary human bronchial epithelial cells (HBECs; Lonza, Basel, Switzerland) were expanded to passage 2, then seeded onto 0.4 μm Corning® HTS Transwell®-24 well permeable supports (Sigma-Aldrich, St Louis, MO) and differentiated at Air-Liquid Interface (ALI) using the PneumaCult™ media system (STEMCELL Technologies, Vancouver, British Columbia, Canada) according to manufacturer’s protocol.

Once fully differentiated, cells were stimulated for 24-48 h by basolateral addition of human recombinant proteins IL-4 and IL-13 or IL-17A and TNFα (all 30 ng/ml each; R&D Systems, Minneapolis, MN) diluted in PneumaCult™-ALI medium.

### EV isolation

Cells stimulated with cytokines for 48 h or left untreated were incubated with 200 μl PBS (Thermo Fisher Scientific, Waltham, MA) apically for 2 h which was then collected and pooled (hereafter called apical washes). In parallel, the media on the basolateral side of the cells was also collected. All samples were immediately stored at − 80 °C.

**Fig. 1:**
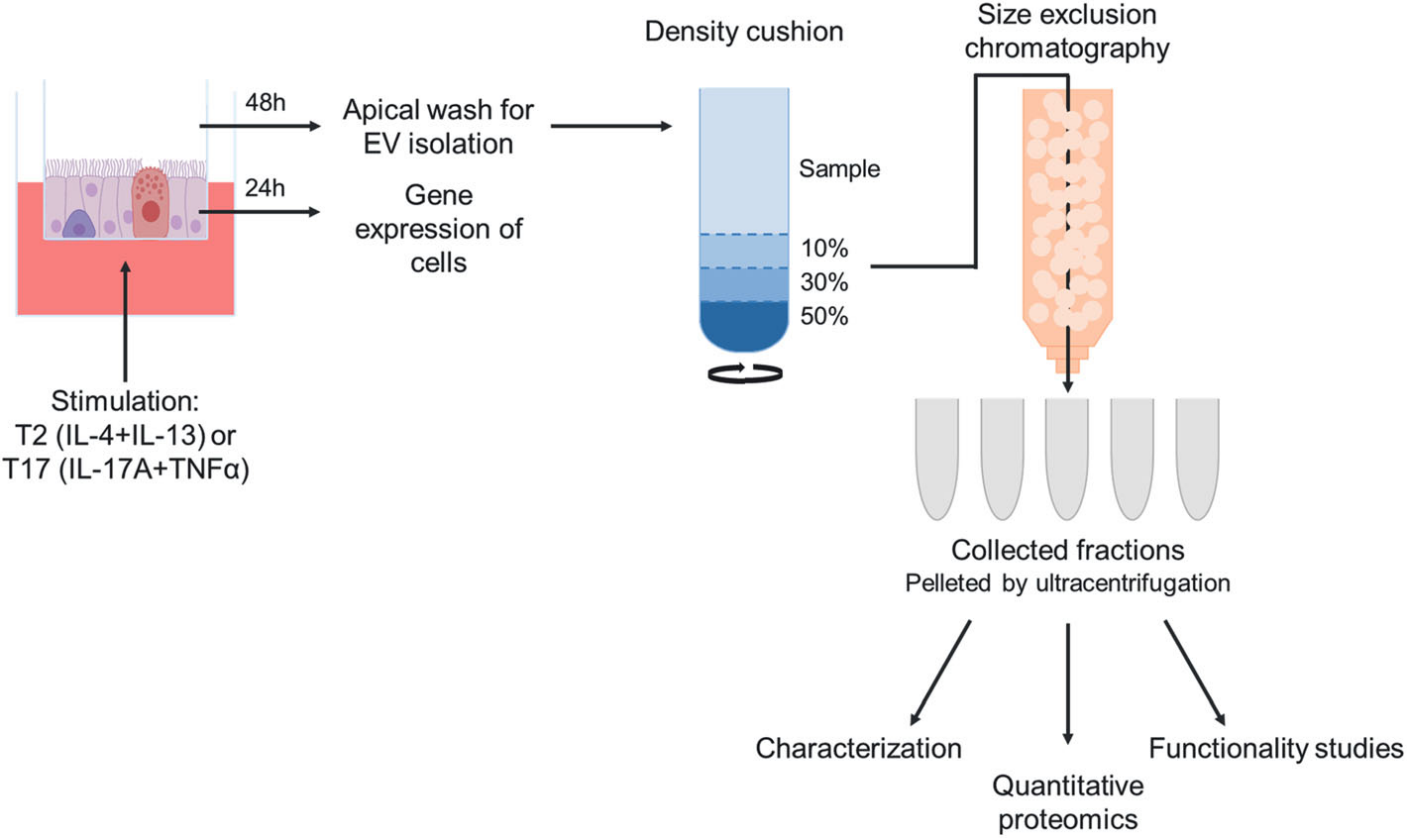
Schematic overview of the experimental workflow. Human bronchial epithelial cells were cultured at air-liquid interface, stimulated with T2 (IL-4 and IL-13) or T17 (IL-17A + TNFα) cytokines or left untreated. Cellular gene expression was analyzed, extracellular vesicles were isolated, and the characteristics and protein cargo of the extracellular vesicles released during the different conditions were evaluated

The methods applied here for isolating EVs have been described in detail previously for isolation of EVs from plasma [23] (Fig. 1). In brief, the apical washes and basolateral media samples were centrifuged at 300×g for 10 min at 4 °C to remove debris. Six milliliter sample was then loaded onto iodixanol density cushions (Optiprep, Sigma-Aldrich) and centrifuged for 2 h at 4 °C (SW 41 Ti swinging bucket rotor at 97,000 × g_avg_, k-factor: 265.1, Beckman Coulter, Brea, CA). After centrifugation, the interphase between 10 and 30%, consisting of the majority of vesicles, was collected in a total volume of 1 ml. This was then run through size exclusion chromatography consisting of Telos columns (Kinesis, Cambridgeshire, UK) packed with sepharose CL-2B beads (GE Healthcare Life Sciences, Marlborough, MA) and 30 fractions of 0.5 ml were collected. Where applicable, fractions were pooled (fractions 1–6, 7–12, 13–18, 19–24, and 25–30), concentrated by centrifugation for 70 min at 4 °C (TLA 100.3 rotor at 116,000 × g_avg_, k-factor: 57.6, Beckman Coulter) and finally the pellets were resuspended in PBS.

### Nanoparticle tracking analysis

Samples were diluted in PBS to have suitable particle concentration for measurement. Measurements were performed with ZetaView PMX 110 (Particle Metrix, Meerbusch, Germany) with the camera sensitivity set to 80 and the shutter set to 100. Data was analysed using the ZetaView analysis software (version 8.2.30.1) with minimum and maximum size of 5 nm and 5000 nm respectively and minimum brightness of 20.

### Protein concentration determination

Samples were lysed with RIPA buffer and sonicated 3 × 5 min with vortexing before protein concentration was determined using microBCA assay kit according to manufacturer’s instructions (Pierce™ microBCA Protein Assay Kit, Thermo Fisher Scientific). For functional studies, protein concentration was determined using Qubit 3.0 Fluorometer (Thermo Fisher Scientific) according to manufacturer’s instructions.

### Electron microscopy

Formvar/carbon-coated copper grids (Ted Pella, Redding, CA) were glow discharged before samples were loaded. Grids and samples were incubated for 15 min, fixed sequentially in 2% paraformaldehyde and 2.5% glutaraldehyde, and contrasted in 2% uranyl acetate. The preparations were examined using LEO 912AB Omega electron microscope (Carl Zeiss NTS, Jena, Germany).

### Western blot

Thirty μl from each SEC fraction or 15 μl from the pooled fractions were separated on 10% polyacrylamide gels and further transferred onto PVDF membranes using a Trans-Blot Turbo Transfer system (Bio-Rad, Hercules, CA). Membranes were blocked using TBS containing 0.01% Tween-20 (TBST) and 5% non-fat dry milk and then incubated with primary antibodies against calnexin (1:1000; clone H-70; Santa Cruz Biotechnology, Dallas, TX) and flotillin-1 (1:1000; clone H-104, Santa Cruz Biotechnology) diluted in TBST containing 0.25% non-fat dry milk over night at 4 °C. Following this, membranes were washed in TBST before incubation with secondary antibodies (donkey anti-rabbit IgG HRP-linked F(ab′)_2_ fragment 1:10,000 dilution, or sheep anti-mouse IgG HRP-linked F(ab′)_2_ fragment 1:10,000 dilution, GE Healthcare) diluted in TBST containing 0.25% non-fat dry milk. Bands were visualised using ECL Prime Western Blotting Detection (GE Healthcare) on VersaDoc 4000 MP (Bio-Rad).

### EV proteomic profiling

Proteomic profiling was performed by the Proteomics Core Facility at the Sahlgrenska Academy, University of Gothenburg. EV samples (40 μg/sample) were digested using the filter-aided sample preparation as previously described [23, 24]. Digested peptides were labelled using TMT 10-plex isobaric mass tagging reagents (Thermo Scientific) according to manufacturer’s instructions. Sodium deoxycholate was removed by acidification with trifluoroacetic acid and the sample was further purified using High Protein and Peptide Recovery Detergent Removal Resin (Thermo Fisher Scientific). The TMT-set was fractionated into eight fractions using Pierce High pH Reversed-Phase Peptide Fractionation Kit (Thermo Fisher Scientific).

Each fraction was analysed on Orbitrap Fusion Tribrid mass spectrometer (Thermo Fisher Scientific) interfaced with nLC 1200 liquid chromatography system. Peptides were trapped on an Acclaim Pepmap 100 C18 trap column (100 μm × 2 cm, particle size 5 μm, Thermo Fisher Scientific) and separated on an in-house constructed analytical column (300 × 0.075 mm I.D.) packed with 3 μm Reprosil-Pur C18-AQ particles (Dr. Maisch, Ammerbuch-Entringen, Germany) using a 75 min gradient from 7 to 80% acetonitrile in 0.2% formic acid. The Orbitrap Fusion Tribrid mass spectrometer was operated in data-dependent MultiNotch MS3 mode. The full scans were acquired at a resolution of 120,000 and the MS2 scans were performed in the ion trap using a collision energy of 35%. The 10 most intense fragment ions were selected for further fragmentation using HCD and a collision energy of 65%. The MS3 scans were acquired at a resolution of 60,000.

The data files for the set were merged for identification and relative quantification using Proteome Discoverer version 1.4 (Thermo Fisher Scientific). Search was against *Homo sapiens* Swissprot Database version November 2017 (Swiss Institute of Bioinformatics, Switzerland) using Mascot 2.5 (Matrix Science) as a search engine with precursor mass tolerance of 5 ppm and fragment mass tolerance of 500 mmu. Tryptic peptides were accepted with zero missed cleavage, variable modifications of methionine oxidation and fixed cysteine alkylation, TMT-label modifications of N-terminal and lysine were selected. The sum of the control samples were used as denominator and for calculation of the ratios. Percolator was used for the validation of identified proteins, missing values was replaced by the software and the quantified proteins were filtered at 1% FDR and grouped by sharing the same sequences to minimize redundancy. Only peptides unique for a given protein were considered for quantification.

### Cell transcriptomics

RNA isolation and next-generation sequencing was performed as described previously [11] (data from IL-4 + IL-13 stimulated cells is the same as used here). In short, after 24 h stimulation, HBEC-ALI cultures were lysed in QIAzol Lysis Reagent (QIAGEN, Hilden, Germany) and RNA isolated using the RNeasy Mini Kit (QIAGEN) following the manufacturer’s instructions and RNA integrity was assessed (Bioanalyzer 2100, Agilent, Santa Clara, CA). RNA was converted to mRNA libraries using the TruSeq Stranded mRNA kit (Illumina, San Diego, CA) and sequenced using a High Output flow cell on an Illumina NextSeq500 in 2 × 76 cycles. Differential gene expression was assessed with DESeq2 [25] using raw counts as input. Genes were considered significantly differentially expressed if they had a q < 0.05 with Benjamini-Hochberg multiple correction.

For quantitative PCR (qPCR), total RNA isolated from cells as described above was converted into cDNA using the High-Capacity cDNA Reverse Transcription Kit (Applied Biosystems, Waltham, MA). qPCR was run using the comparative Ct method on a QuantStudio 7 Flex Real-Time PCR system (Thermo Fisher Scientific) using TaqMan™ Fast Advanced Master Mix (Thermo Fisher Scientific). Gene expression was determined with probes from TaqMan Real-Time PCR Assays (*CCL26, NOS2, ANO1, POSTN, SLC26A4, CSF3, PI3* and *CCL20*, Thermo Fisher Scientific) and normalized against the housekeeping gene *ACTB*.

### Neutrophil chemotaxis

Neutrophils were isolated from whole peripheral blood from healthy volunteers using the MACSxpress® Whole Blood Neutrophil Isolation Kit (Miltenyi Biotec, Bergisch Gladbach, Germany) according to protocol and resuspended in media consisting of RPMI 1640 (GE Healthcare) supplemented with 110 μg/ml of sodium pyruvate (Sigma-Aldrich). Media alone, media with EVs or with 5% FBS (used as positive control, as previously described [26]) were added to the lower wells of a 48-Well Micro Chemotaxis Chamber (NeuroProbe, Gaithersburg, MD) on top of which a gelatin-coated 8 μm polycarbonate membrane (NeuroProbe) was placed. After assembly of the chamber, 170,000 neutrophils were added to the upper wells and the chamber was incubated at 37 °C for 3 h. The chamber was then disassembled and number of migrated cells in the lower wells counted using a Bürker chamber with Türk staining solution (Sigma-Aldrich).

### Bioinformatics and data analysis

Lists of proteins associated with EVs used to create Table 1 were obtained from EVpedia [27] and Vesiclepedia [28]. Proteins related to EV biogenesis and release were collated from relevant reviews [29–33] and TIBCO Spotfire was used to analyse this data in Supplementary Fig. 2. GO Term Finder [34] and Ingenuity Pathway Analysis (QIAGEN) were used for pathway analysis, Qlucore Omics Explorer (Qlucore, Lund, Sweden) for principal component analysis and GraphPad Prism v.8 (GraphPad Software, San Diego, CA) for performing paired t-tests and mixed-effects analysis unless otherwise stated.

**Table 1:** Common EV proteins detected in the epithelial EV proteome

Protein group	Proteins
Rabs	Rab-1A, −1B, −2A, −3D, −4A, −5A, −5B, −5C, −6A, −7A, −8A
	Rab-10, −11A, − 13, − 14, − 18
	Rab-20, − 21, − 23, − 25, −27A, −27B
	Rab-34, − 35
Annexins	Annexin A1, A2, A3, A4, A5, A6, A7, A11
Tetraspanins	CD9, CD63, CD81, CD82, CD151, TSPAN6, TSPAN8, TSPAN14
Common EV markers	MHC class I, MHC class II, Ezrin, Flotillin-1, Flotillin-2, Cofilin-1, Profilin-1, CD59, 14–3-3 protein (beta/alpha, epsilon, eta, gamma, sigma, theta, zeta/delta),
Heat shock proteins	HSPA1A, HSPA4, HSPA8, HSPH1, HSPB1, HSP90AA1, HSP90AB1
ESCRT	ESCRT-0 – HGS
	ESCRT-I – VPS-28, VPS-37B, VPS-37C, TSG101, MVB-12A
	ESCRT-II – VPS-36
	ESCRT-III – CHMP1A, CHMP1B, CHMP2A, CHMP2B, CHMP4A, CHMP4B, CHMP5, CHMP6, IST1
	ESCRT accessory – VPS-4A, VPS-4B, VTA1, Clathrin, Alix

List of common EV proteins were obtained from the databases EVpedia and Vesiclepedia.

## Results

### Primary HBEC-ALI release small EVs on their apical side

EVs were isolated from apical and basolateral compartments of HBEC-ALI (Fig. 1) and nanoparticle tracking analysis demonstrated that the majority of particles were found in SEC fractions 8–10 from the apical samples (Supplementary Fig. 1a ). Western blot confirmed the presence of the EV marker flotillin-1 and electron microscopy displayed vesicles with size ≤100 nm (Supplementary Fig. 1b-c ) in these fractions. Soluble proteins eluted beyond SEC fraction 16 (Supplementary Fig. 1a ). Only few particles with no SEC peak nor flotillin-1 were detected in the basolateral sample (Supplementary Fig. 1a-b ). Thus, henceforth only apical samples were analyzed.

**Fig. 2:**
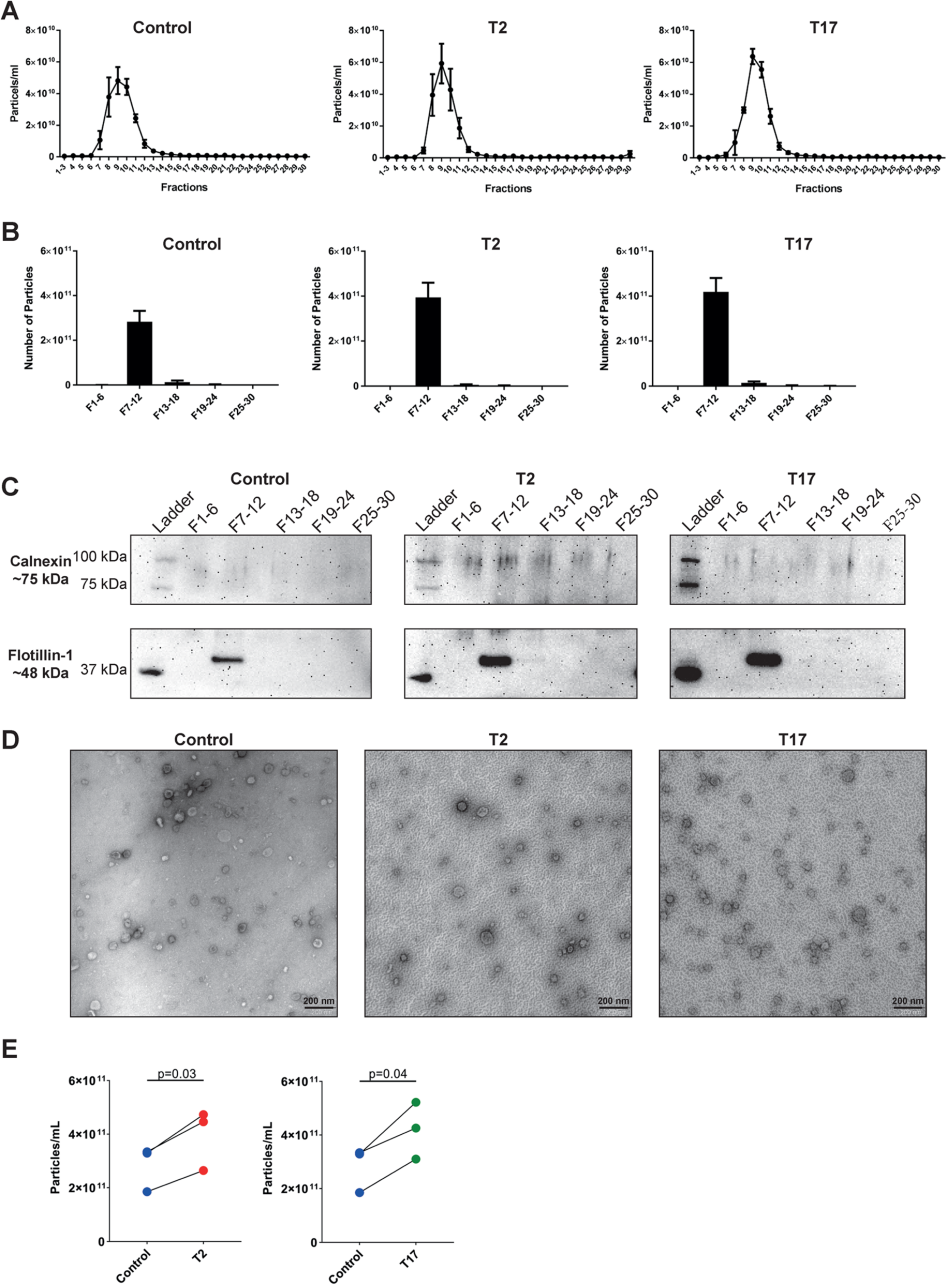
T2 and T17 cytokines induce increased release of extracellular vesicles from HBECs at air-liquid interface. Cells were stimulated with T2 (IL-4 + IL-13) or T17 (IL-17A + TNFα) cytokines or left untreated (Control) and vesicles were isolated from the apical side. **a** Number of particles was measured in each fraction of the size exclusion chromatography by nanoparticle tracking analysis. (*n* = 3) Data are presented as the mean and SEM. **b** Number of particles was measured in each pool, each consisting of six fractions from the size exclusion chromatography, by nanoparticle tracking analysis. (*n* = 3) Data are presented as the mean and SEM. **c** Presence of the extracellular vesicle marker flotillin-1 and the endoplasmic reticulum protein calnexin were determined by Western blot in all pools. **d** Size and morphology of vesicles was determined by electron microscopy. Scale bars are 200 nm in the electron micrographs. **e** Individual particle concentrations for the pools consisting of fractions 7–12 from b are plotted. *p*-values calculated using paired t-test

HBEC-ALI stimulated basolaterally with T2 or T17 cytokines, or media alone, released EVs that eluted in SEC fractions 7–12 which were again containing flotillin-1 but not the endoplasmic reticulum marker calnexin and were of size ≤100 nm (Fig. 2a-d). The F7–12 pools were used for subsequent studies. Stimulation with T2 and T17 cytokines significantly increased the release of EVs as compared to control cells (Fig. 2e). Together, these results demonstrate that primary HBEC-ALI cultures release EVs at their apical side and that this release is increased when the cells are stimulated with T2 or T17 cytokines.

### Induction of specific gene expression signatures upon T2 and T17 cytokine stimulation

**Fig. 3:**
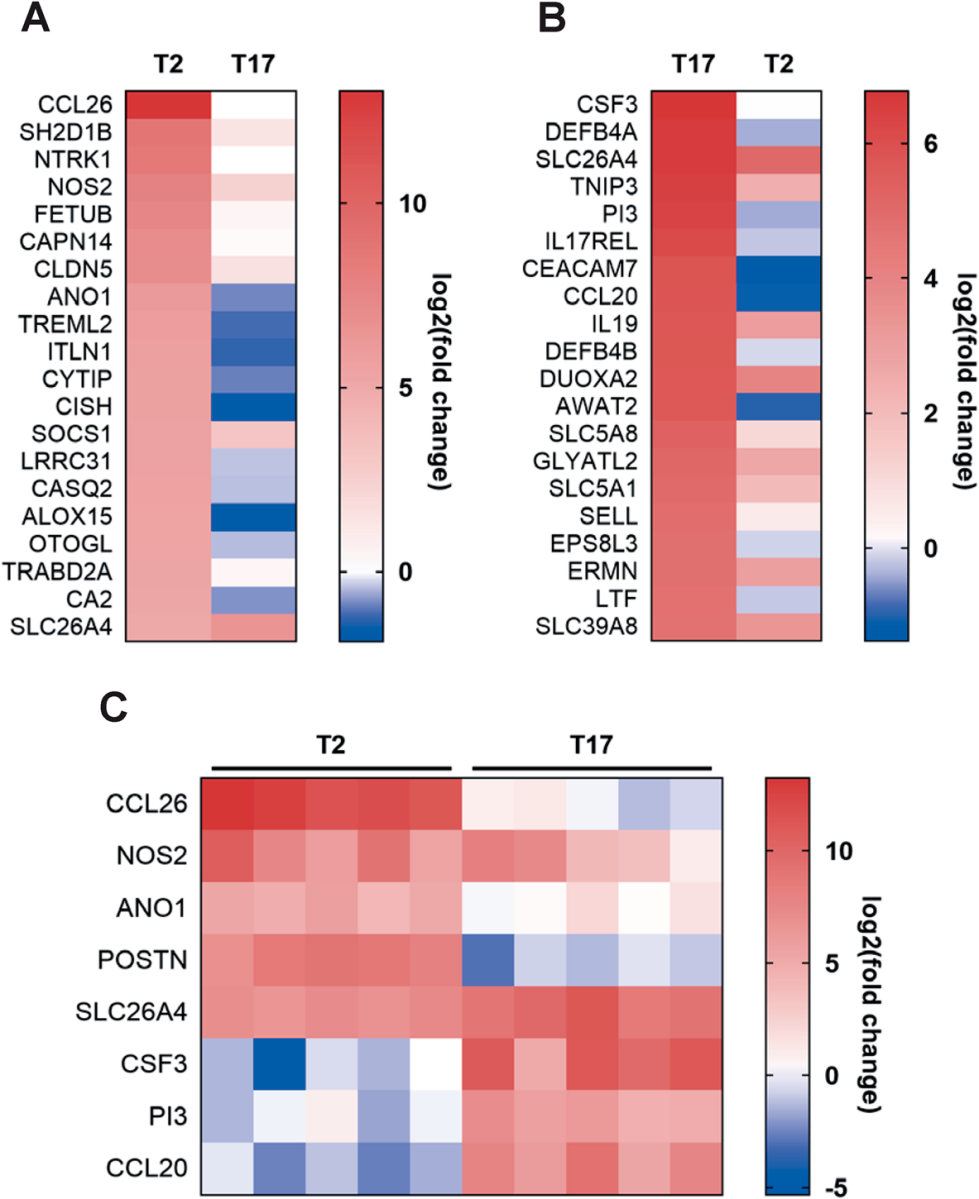
T2 and T17 cytokines induce specific gene expression signatures in HBECs at air-liquid interface. **a**-**b** Next-generation sequencing was used to analyze RNA isolated from a single donor after 24 h stimulation. Top 20 upregulated genes after T2 (IL-4 + IL-13, **a**) and T17 (IL-17A + TNFα, **b**) stimulation. **c** Validation of a selection of upregulated genes by quantitative PCR (*n* = 5, each column represents expression in one donor). All data are expressed as log2 fold change compared to non-stimulated control

To determine the response to T2 and T17 cytokines, the gene expression in cytokine-stimulated HBEC-ALI cultures was analyzed by RNA sequencing which resulted in 3646 and 5379 differentially expressed genes, respectively. Analysis of the 20 most upregulated genes for each stimulation (Fig. 3a-b, and Supplementary Table 1) revealed several genes previously described in context of T2 or T17 immune responses. Genes induced by T2 stimulation included C-C motif chemokine 26 (*CCL26*; also called eotaxin-3) [35], inducible nitric oxide synthase (*NOS2*) [36] and arachidonate 15-lipoxygenase (*ALOX15*) [37], whereas genes induced by T17 stimulation included granulocyte colony-stimulating factor (*CSF3*) [10], C-C motif chemokine 20 (*CCL20*) [38], and the putative IL-17 receptor E-like (*IL17REL*) [39]. A few genes such as the ion exchanger pendrin (*SLC26A4*) [40] were however shown to be induced by both T2 and T17 stimulation. The expression of a selection of genes was confirmed by qPCR in additional HBEC donors after stimulation (Fig. 3c).

The increased release of EVs upon cytokine stimulation (Fig. 2e) might be explained by an increased expression of genes encoding proteins involved in EV biogenesis, transport, or release (Supplementary Fig. 2). A total of 61 such genes were found to be differentially expressed upon T2 and/or T17 cytokine stimulation and out of these, 95% were upregulated compared to non-stimulated cells (Supplementary Fig. 2).

Together, these results demonstrate that distinct gene expression signatures reflecting immune responses involved in airway inflammation, as well as proteins involved in EV biogenesis and release, are induced in airway epithelial cells upon T2 and T17 cytokine stimulation.

### Identification of the bronchial epithelium EV proteome by mass spectrometry

**Fig. 4:**
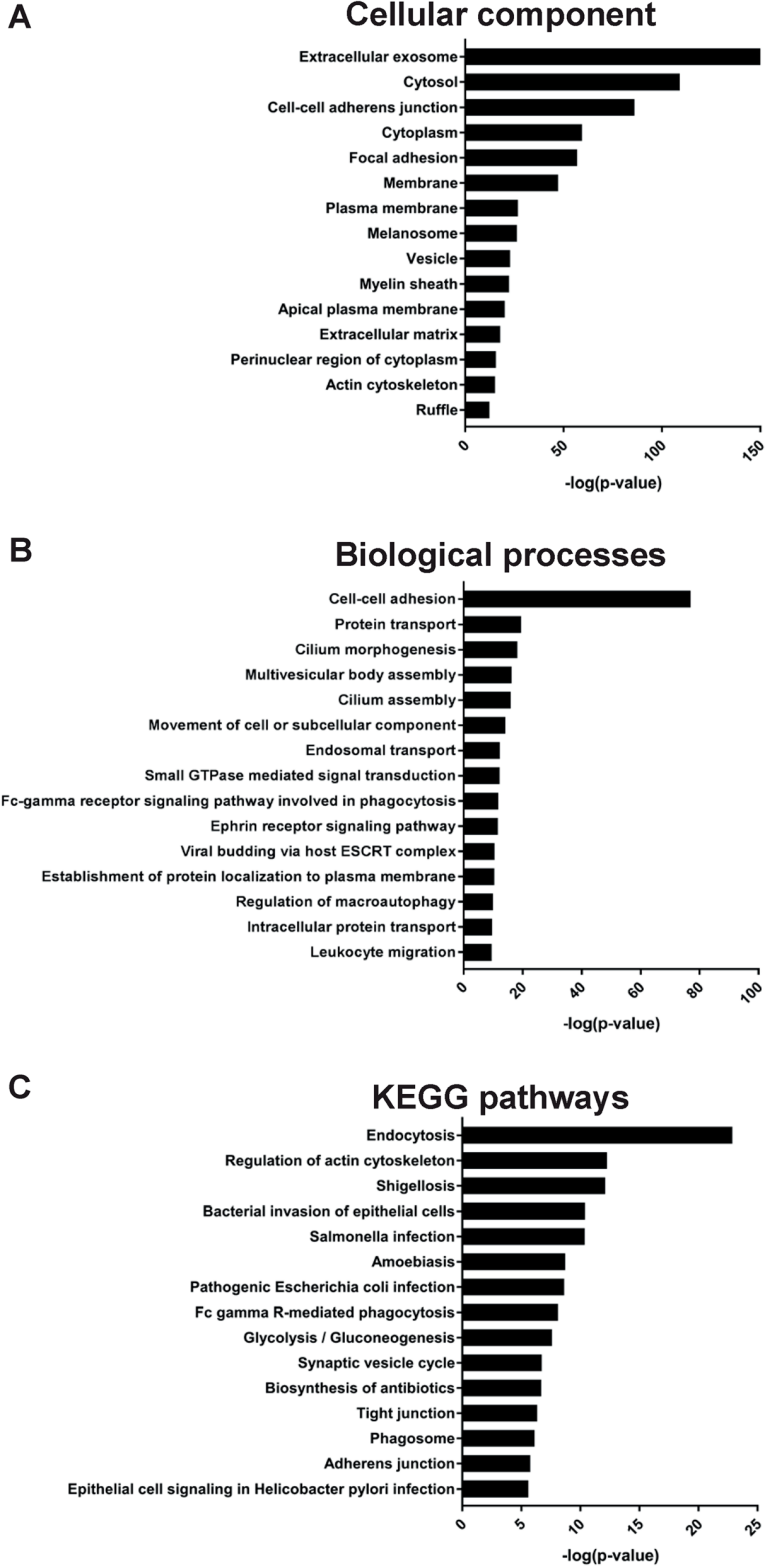
GO Terms associated with the proteome of bronchial epithelium-derived extracellular vesicles. Gene Ontology Term Finder was used to determine the most enriched cellular compartments (**a**), biological processes (**b**) and KEGG pathways (**c**) associated with proteins detected by mass spectrometry in isolated vesicles, compared to the genome frequency. The 15 most enriched terms (based on *p*-value) in each category are displayed

To further characterize the bronchial epithelium-derived EVs, mass spectrometry was used to profile the proteome of EVs isolated from three donors. In total, 1358 proteins were identified in all samples (Supplementary Table 2). Of 121 top-ranking EV proteins extracted from the EVpedia [27] and Vesiclepedia [28] databases, 101 (83%) were represented in the HBEC-ALI-derived EVs. This included several common EV proteins such as Rab proteins, annexins and tetraspanins (Table 1). Gene ontology analysis focused on cellular compartments showed that over 60% of the proteins were associated with the top term “extracellular exosome” and there was strong association with the term “apical plasma membrane”, confirming that the isolated vesicles were specifically released from the apical side (Fig. 4a). Further analysis, focused on biological processes, revealed association between the epithelium-derived EV proteome and the terms “multivesicular-body assembly”, “endosomal transport” and “viral budding via host ESCRT complex”, which may suggest that at least a part of the isolated EVs have an endosomal pathway-associated biogenesis (Fig. 4b). Interestingly, “cell-to-cell adhesion” and “leukocyte migration” were also among the top-15 terms. Furthermore, top-15 KEGG pathways associated with the EV proteome also showed association to the endosomal pathway (“endocytosis”) and “adherence junction”/“tight junctions” (Fig. 4c). Together, this indicates that EVs of high purity have been isolated and the pathway analysis suggests possible biological roles such as leukocyte migration and cell adhesion.

### The proteome of EVs is altered upon T2 and T17 cytokine stimulations

**Fig. 5:**
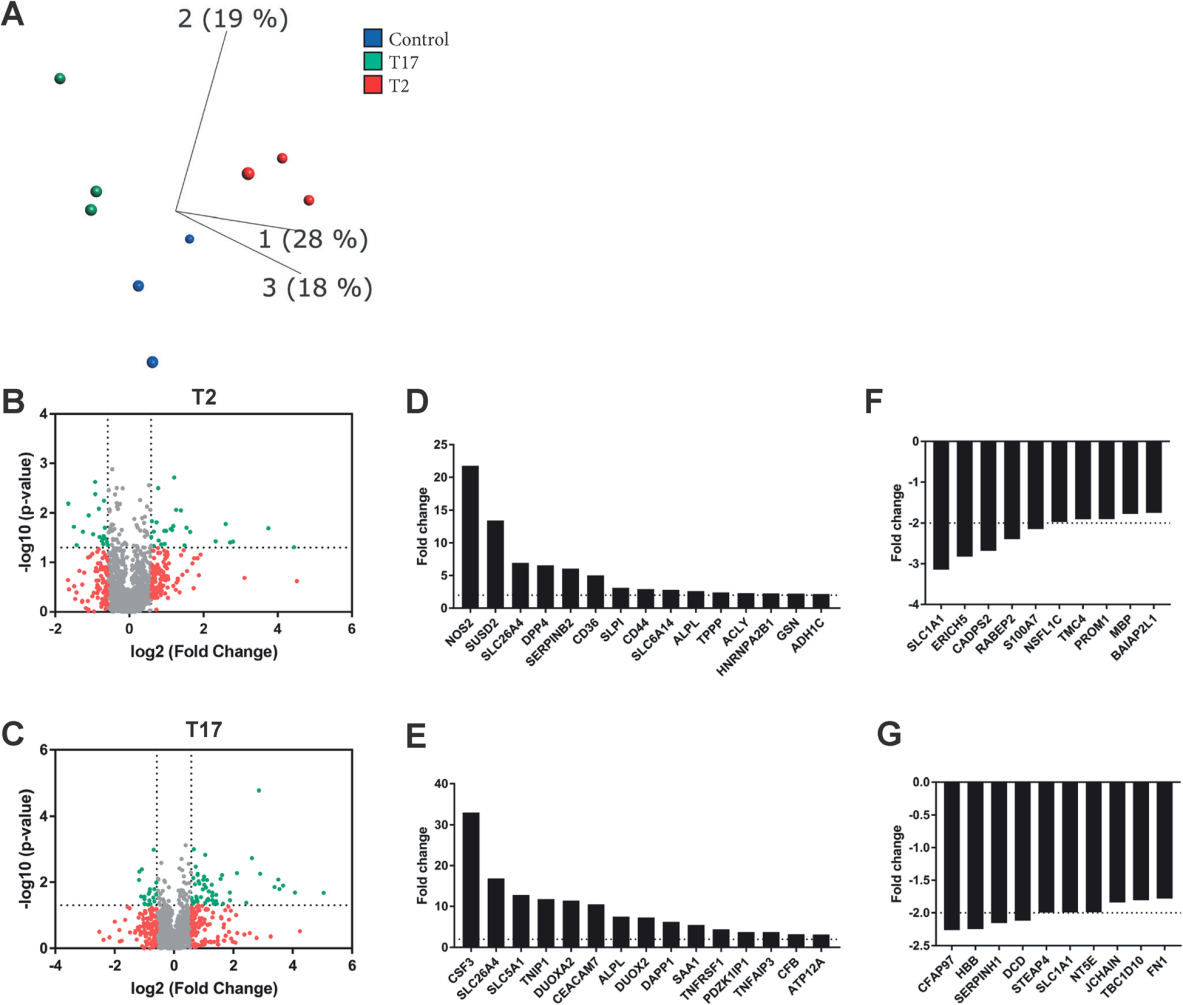
The proteome of bronchial epithelium-derived extracellular vesicles is altered upon T2 and T17 stimulation. Quantitative proteomics (tandem mass tag; TMT) was used to determine the influence of T2 and T17 cytokines on bronchial epithelium-derived extracellular vesicles. **a** Principle component analysis illustrating the relationship between T2-derived EVs (red), T17-derived EVs (green) and EVs isolated under non-stimulated condition (blue) (*n* = 3). b-c) Volcano plots of the proteome after T2 (**b**) and T17 (**c**) stimulation. Dotted lines indicate cut offs, which is 1.3 on the Y-axis (corresponding to *p* < 0.05) and 0.67 on the X-axis (corresponding to fold change> 1.5). d-e) Top 15 upregulated proteins in extracellular vesicles derived from bronchial epithelial cells after T2 (**d**) and T17 (**e**) stimulation based on fold change compared to non-stimulated cells f-g) Top 10 downregulated proteins in extracellular vesicles derived from bronchial epithelial cells after T2 (**f**) and T17 (**g**) stimulation based on fold change compared to non-stimulated cells

As T2 and T17 cytokine stimulation altered the HBEC-ALI transcriptome and increased EV release, quantitative proteomics was performed to determine if also the EV proteome was altered. Principle component analysis showed clear separation between EVs isolated from non-stimulated versus stimulated HBEC-ALIs, as well as between EVs isolated from T2 and T17 cytokine stimulated cells (Fig. 5a).

In total 26 and 55 proteins were significantly upregulated after treatment with the T2 and T17 cytokines, respectively (Fig. 5b-e). Under T2 conditions, the most upregulated EV proteins were inducible nitric oxide synthase (NOS2), sushi domain containing 2 (SUSD2) and dipeptidyl peptidase-4 (DPP4). Proteins most upregulated in T17-derived EVs were granulocyte-colony stimulating factor (CSF3), TNF-α induced protein 3-interacting protein 1 (TNIP1) and dual oxidase 2 (DUOX2). None of these proteins were significantly upregulated in EVs in response to the other stimulus, however, some proteins, such as pendrin (SLC26A4) were increased in EVs released under both stimuli. The EVs contained reduced levels of 19 proteins in response to each stimulus (Figs. 5f-g) such as S100A7 and prominin-1 (PROM1) under T2 stimulation, and cilia and flagella associated protein 97 (CFAP97) and dermacidin (DCD) under T17 stimulation. Further, differentially abundant EV-proteins correlate well with expression of the corresponding genes in the stimulated parent epithelial cells (Supplementary Fig. 3a-b).

Ingenuity Pathway Analysis was used to predict upstream regulators of the proteins significantly altered in cytokine-derived EVs. Under T17 conditions, TNF was the only predicted activator, whereas under T2 conditions, both IL-13 and IL-4 had positive activation scores. These findings together confirm that the observed changes in EV proteins are directly related to the stimuli applied.

### T17 cytokines induce EV-proteins involved in neutrophil migration

**Fig. 6:**
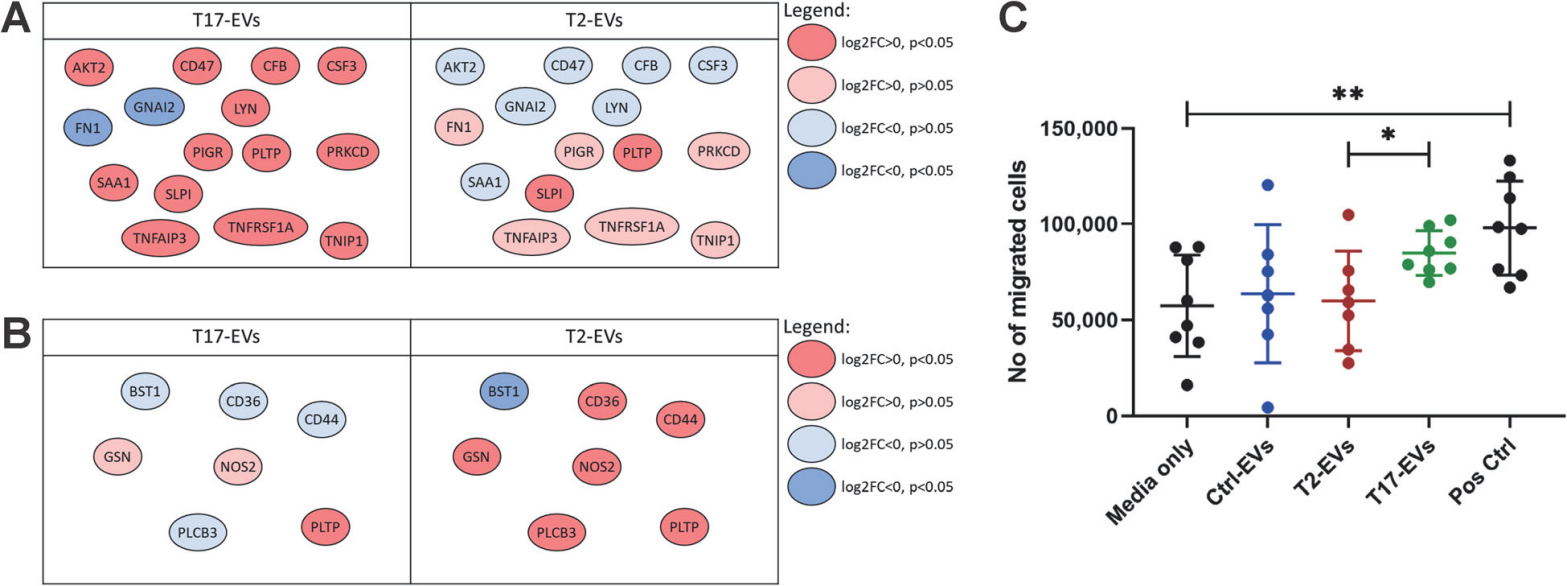
The altered T17 EV proteome contains proteins with activating effects on neutrophil migration. **a-b** Differential levels of proteins involved in the pathways related to neutrophil migration identified in Table 2 that were predicted to be activated by T17-EV proteins (**a**) or inhibited by T2-EV proteins (**b**). **c** Peripheral blood neutrophils allowed to migrate towards; media only as negative control, EVs (30 μg/ml) from non-stimulated epithelial cells (blue) or cells stimulated with T2 (red) or T17 (green) cytokines, or the positive control (5% FBS). Each dot represents the median number of migrated cells after 3 h of incubation (2–4 replicate wells) for one donor, with group means ± SD indicated by lines. (*n* = 7–8) Group means were compared using mixed-effects analysis. Only adjusted *p*-values ≤0.05 after Tukey’s multiple comparisons test are shown (*: *p* ≤ 0.05, **: *p* ≤ 0.01)

Ingenuity Pathway Analysis also suggested that pathways related to neutrophil recruitment might be activated by T17-derived EV proteins whereas the opposite was seen for T2-derived EV proteins (Table 2). Implicated EV proteins were for example CSF3, TNFAIP3, TNIP1 and SAA1 that were increased under T17 conditions (Table 2 and Fig. 6a) and NOS2, CD36 and CD44 which were increased under T2 conditions (Table 2 and Fig. 6b).

**Table 2:** Pathways related to neutrophil migration predicted to be differentially activated by proteins in T2- and T17-EVs

	T17-EVs		T2-EVs	
Pathway	Activation z-score	Implicated proteins	Activation z-score	Implicated proteins
Migration of neutrophils	1.929	AKT2, CD47, FN1, SAA1, TNFRSF1A		
Quantity of neutrophils	1.504	CD47, CSF3, GNAI2, LYN, PRKCD, SLPI, TNFAIP3, TNFRSF1A		
Cell movement of neutrophils	1.367	AKT2, CD47, CFB, CSF3, FN1, GNAI2, LYN, PIGR, PLTP, SAA1, TNFAIP3, TNFRSF1A, TNIP1	−0.287	BST1, CD36, CD44, GSN, NOS2, PLCB3, PLTP
Infiltration by neutrophils	0.468	CFB, CSF3, PLTP, TNFAIP3, TNFRSF1A, TNIP1	−1.77	CD36, CD44, NOS2, PLCB3, PLTP
Immune response of neutrophils	−0.134	CD47, CSF3, FN1, LYN, SAA1		
Adhesion of neutrophils			−0.655	CD44, NOS2, PLCB3, PLTP

This table contains all pathways containing the term “neutrophil” that were given an activation z-score using Ingenuity Pathway Analysis on the EV proteomics. Activation z-score > 0 suggests activated pathway, Activation z-score < 0 suggests inhibited pathway. See data for implicated proteins in Fig. 6.

To test this possible function in vitro, the capacity of HBEC-ALI-derived EVs to drive migration of neutrophils isolated from blood was determined. Although not reaching statistical significance, there was a trend for T17-derived EVs to induce an increased neutrophil migration compared to media alone, whereas the T2-derived EVs, as suggested by the pathway analysis, were unable to do so. Importantly, the migration of neutrophils towards T17-derived EVs was significantly higher than that towards T2-derived EVs (Fig. 6c). The increase in migration induced by T17-derived EVs showed, in similarity with the positive control, less variation among donors compared to the other EV conditions, suggesting a more true induction of neutrophil migration.

## Discussion

In this study we show that EVs are secreted from the apical side of HBEC-ALI cultures and can be isolated at high purity using a combination of density cushion centrifugation and SEC. The HBEC-ALI cultures were stimulated with pro-inflammatory cytokines to mimic T2 or T17 asthma endotypes, which led to increased release of EVs and altered composition of the EV proteome. Regulation of most EV proteins was stimulus-specific, closely reflecting the parent cell transcriptome, pointing to the possibility of distinguishing T2- or T17-driven inflammation based on protein profiles in bronchial epithelium-derived EVs. Additionally, we observed that the EVs may drive a stimulus-dependent functional response in recipient cells, as exemplified by a suggested effect on neutrophil chemotaxis both in silico and in vitro for T17-derived EVs which was in contrast to T2-derived EVs.

The HBEC-ALI-derived EVs were isolated with our previously developed isolation method [23] which separates EVs from soluble proteins and cell debris. Isolated EVs were carefully characterized and shown to be smaller than 100 nm in diameter and of high purity. Although it has previously been suggested that EVs can be isolated from the basolateral side of HBEC-ALI cultures [41, 42], we could only detect EVs in the apical compartment. To exclude this as a technical artefact we repeated the EV isolation using ultracentrifugation as an alternative method. However, still no EVs in the basolateral samples were detected (data not shown) and the reasons for this was not investigated further. Based on the available data we suggest that EVs released apically from bronchial epithelium in vitro are reflective of those that are present in the central airways, making them a relevant model for studying EVs reflecting local processes in airway diseases. Apical EVs may be involved in signal propagation in the airways and thus help maintain homeostasis or propagate pathogenic signals as suggested previously [18, 43].

HBEC-ALI cultures stimulated with T2 or T17 cytokines induced expression of several genes previously described to be induced upon similar conditions or that have been found to be altered in asthmatic airways, which shows the translatability of our model. Genes upregulated by T2 cytokines and that have been associated with T2 asthma included the eosinophil chemoattractant *CCL26* [35] and *POSTN* (periostin) which is implicated in extracellular matrix remodeling [9], both biological processes associated with asthma. Of the genes induced by T17 cytokines and that have been described in the context of T17 inflammation, *CSF3* stimulates bone marrow granulopoiesis and is involved in neutrophilic inflammation [44] and *CCL20* is a chemoattractant for Th17 cells and neutrophils [45]. This regulated gene expression clearly indicates that our model is biologically relevant and useful for studying airway inflammatory mechanisms.

Both stimuli increased the number of released EVs compared to untreated cells, which is in line with previous findings demonstrating increased EV secretion from epithelial cells upon IL-13 treatment [20]. On gene level in the cells, almost 60 genes corresponding to proteins involved in EV biogenesis were upregulated, which could explain the observed increased EV release. To explore whether the gene expression profiles in the cytokine-stimulated cells were reflected on protein level in their EVs, quantitative proteomics was applied. The high correlation between the EV proteomes and the corresponding cell transcriptomes after cytokine stimulation demonstrates that the EVs reflect the phenotype of their source cells. Notably, not all differentially expressed genes were represented in the EV proteome. For instance, *POSTN, CCL26* and *CCL20* were instead found to be released from the cells as soluble proteins (determined by ELISA, data not shown), suggesting that sorting of proteins to different compartments is an active process.

The majority of differentially altered EV proteins and differentially expressed genes are non-overlapping between the two stimuli which is in alignment with reports by Choy et al. for T2/T17 induced gene expression in bronchial epithelial cells [10]. One exception to this pattern is pendrin (SLC26A4), which was increased under both conditions. Pendrin has been associated with mucus production in asthma and chronic obstructive pulmonary disease [40], and thus its expression in bronchial epithelium and presence on their released EVs from either condition suggests that it may drive this process in both inflammatory endotypes, and that epithelial EVs could be involved in propagating a mucus hypersecretory phenotype throughout the airways.

The most increased protein in EVs released upon stimulation with T2 cytokines was NOS2 which has been shown to be upregulated in the respiratory epithelium of patients with asthma [46] and is the main producer of exhaled nitric oxide, a biomarker of T2 asthma [36]. We have previously shown that NOS2 is a prominent component of EVs from nasal lavage [22], however, altered levels of airway NOS2^+^ EVs in asthmatics are yet to be demonstrated. Another increased T2 EV protein was DPP4, which, in line with our data, has previously been shown to be induced by IL-13 in bronchial epithelium of asthmatics and to increase proliferation of fibroblasts and smooth muscle cells [47], consistent with a role in airway remodelling. In addition, studies have demonstrated that DPP4 on in vivo-derived EVs is enzymatically active [48], suggesting that airway DPP4^+^ EVs may participate in these mechanisms in asthma.

The antimicrobial peptide S100A7 was one of the decreased proteins in EVs after T2 stimulation. Previous observations have shown that this peptide and its family members are decreased in the airways of individuals with allergic diseases [22, 49], which is particularly interesting as allergic asthma is commonly associated with T2 inflammation.

Under T17 conditions, CSF3 was the most increased protein in the released EVs. CSF3 stimulates the activity of neutrophils [44, 50], suggesting that EVs released during T17 inflammation may promote neutrophilic airway inflammation. Interestingly, CSF3 has not been reported to be present in EVs prior to this study (EVpedia September 13th 2019). Another asthma-related protein that was increased in T17-derived EVs was CEACAM7, which together with CEACAM6 has been implicated in severe neutrophilic asthma [51]. The presence of these proteins suggests that epithelial EVs may propagate pathogenic processes related to asthma, and that the EV protein set-up may reflect the ongoing inflammatory response.

In addition to CSF3 and its effect on neutrophils mentioned above, pathway analysis of the EV proteomes highlighted differential levels of proteins promoting or inhibiting neutrophil recruitment for T17- and T2-derived EVs, respectively. In contrast, no pathways related to eosinophil migration were found to be associated with the EV proteins altered in response to the cytokine stimulations. This suggests a possible role of bronchial epithelium-derived EVs in T17 versus T2 inflammation. To address this hypothesis, the effect of T17- and T2-derived EVs on neutrophil migration was investigated. Indeed, there was a trend towards an increase in neutrophil migration induced by the T17-derived EVs compared to the media only control, while no such trend was seen for T2-derived EVs. Additionally, the number of migrated neutrophils was significantly higher than that induced by T2-EVs. These findings suggest that EVs released from the bronchial epithelium in response to T17 immune responses may contribute to establishing the neutrophilic airway inflammation associated with T17-driven asthma.

One limitation of our current study is that the cell model used was based on cells from healthy donors treated with cytokines in vitro to reproduce the T2 and T17 asthma endotypes. To validate that the EVs described here are representative of human asthma, further studies are required using airway samples from well-characterized asthmatics.

## Conclusions

This is the first study describing a potential role for bronchial epithelium-derived EVs in T2 and T17-driven airway inflammation. The isolation and characterization of these EVs adds to previous knowledge around the role of the airway epithelium in mediating immune responses associated with respiratory diseases such as asthma. Further, the EV proteomes contain signatures that can differentiate between two types of inflammatory environment, and these EVs may represent a valuable opportunity for future patient stratification strategies.

In summary, our findings suggest that bronchial epithelium-derived EVs may actively promote disease processes associated with asthma and, importantly, provide a novel opportunity for understanding of local airway mechanisms enabling a precision medicine approach to treating asthma.

## Supplementary information

**Additional file 1 Supplementary Fig. 1** Primary HBECs cultured at air-liquid interface release extracellular vesicles on their apical side. The apical side of the cells was washed with PBS and the PBS and the media from the basolateral side were collected. Samples were processed on an Optiprep density cushion followed by size exclusion chromatography. a) Number of particles and amount of protein were measured in each fraction of the size exclusion chromatography by nanoparticle tracking analysis (black bars) and bicinchoninic acid assay (BCA, grey bars), respectively. b) Presence of the extracellular vesicle marker flotillin-1 was determined by Western blot in fractions 6–11. c) Size and morphology of vesicles was determined by electron microscopy. Scale bars are 200 nm in the electron micrographs and 100 nm in the magnifications.

**Additional file 2 Supplementary Fig.  2** Genes involved in EV biogenesis, transport, and release are increasingly expressed upon cytokine stimulation. A list of 106 genes corresponding to proteins involved in processes related to EV generation and release was generated based on previous publications and the expression of those found to be differentially expressed by RNAseq (FDR < 0.05) are shown here, along with hierarchical clustering on the gene expression level.

**Additional file 3 Supplementary Fig. 3** The airway epithelial EV proteomes correlate with their cellular transcriptomes. Proteins differentially abundant between EVs from T2 (IL-4 + IL-13) and T17 (IL-17A + TNFα) stimulated epithelial cells were correlated to the differential expression of their corresponding gene in cells treated under the same condition. a-b) Plots for T2 stimulation (a) and T17 stimulation (b), fold change for EV proteins on the X-axis and fold change for cellular genes on the Y-axis. All values are log2-transformed and using non-stimulated cells, or EVs from these cells, as control. Solid line corresponds to the interpolated correlation with r^2^ and *p*-values as presented in the figures.

**Additional file 4 Supplementary Table 1.** Top 50 genes induced by T2 stimulation followed by top 50 genes induced by T17 stimulation.

**Additional file 5 Supplementary Table 2.** 1358 proteins identified in EVs from bronchial epithelial cells, with differential abundance in EVs from cells after T2 and T17 stimulation.

## Data Availability

Original data can be requested from corresponding authors.

## Abbreviations

ALI: Air-liquid interface
BALF: Bronchoalveolar lavage fluid
EV: Extracellular vesicle
GO: Gene ontology
HBEC: Human bronchial epithelial cell
HBEC-ALI: Human bronchial epithelial cells cultured at air-liquid interface
IL: Interleukin
qPCR: Quantitative PCR
SEC: Size-exclusion chromatography
T2: Type 2
T17: Type 17
