# Supplementary figures and images for: T2 and T17 cytokines alter the cargo and function of airway epithelium-derived extracellular vesicles
Source: Respir Res. 2020 Jun 19;21:155. doi: 10.1186/s12931-020-01402-3 (PMC7304225; doi:10.1186/s12931-020-01402-3)

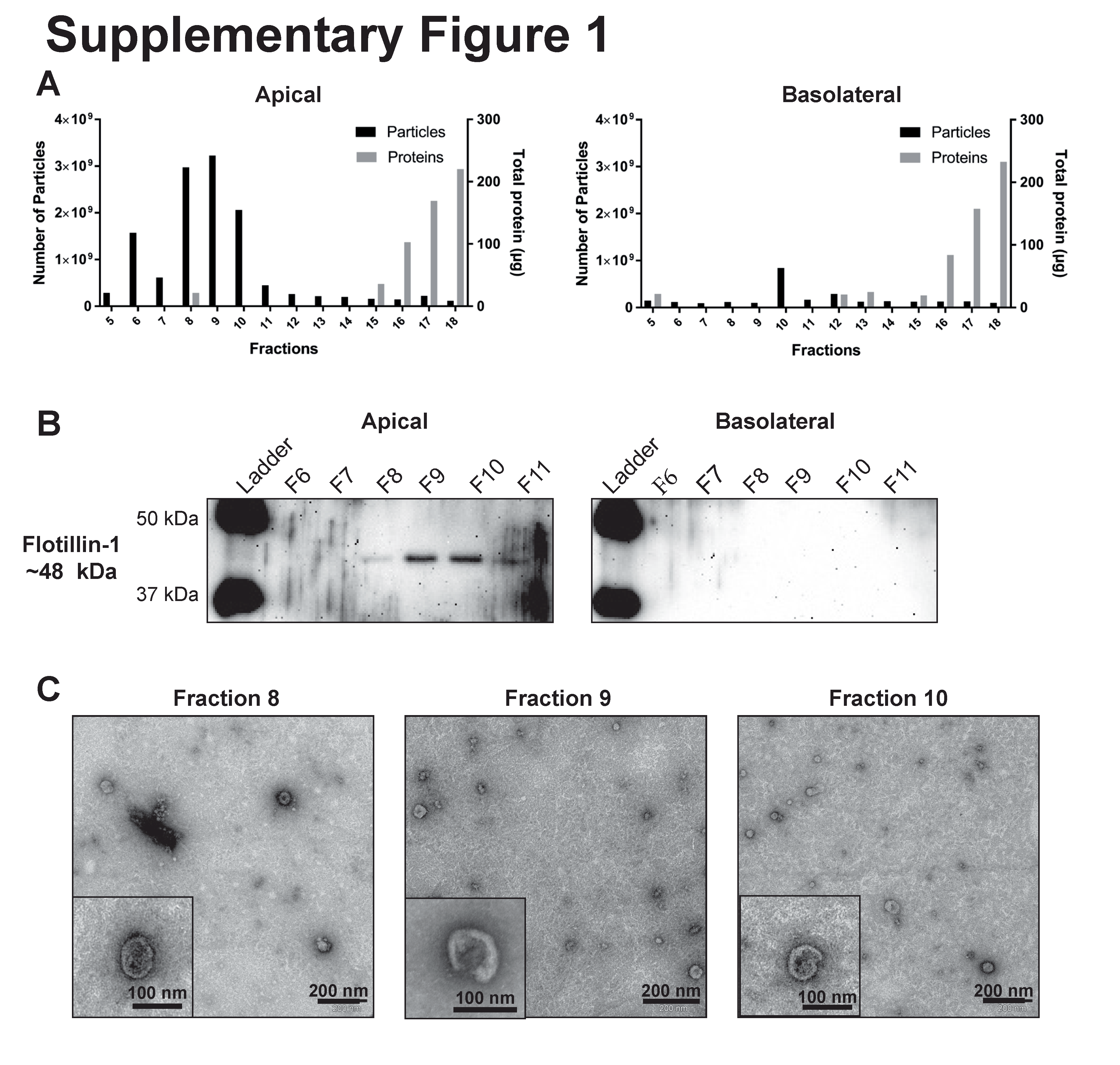

Supplement: Supplementary file 1 — Additional file 1 Supplementary Fig. 1 Primary HBECs cultured at air-liquid interface release extracellular vesicles on their apical side. The apical side of the cells was washed with PBS and the PBS and the media from the basolateral side were collected. Samples were processed on an Optiprep density cushion followed by size exclusion chromatography. a) Number of particles and amount of protein were measured in each fraction of the size exclusion chromatography by nanoparticle tracking analysis (black bars) and bicinchoninic acid assay (BCA, grey bars), respectively. b) Presence of the extracellular vesicle marker flotillin-1 was determined by Western blot in fractions 6–11. c) Size and morphology of vesicles was determined by electron microscopy. Scale bars are 200 nm in the electron micrographs and 100 nm in the magnifications. [file 12931_2020_1402_MOESM1_ESM.tif]

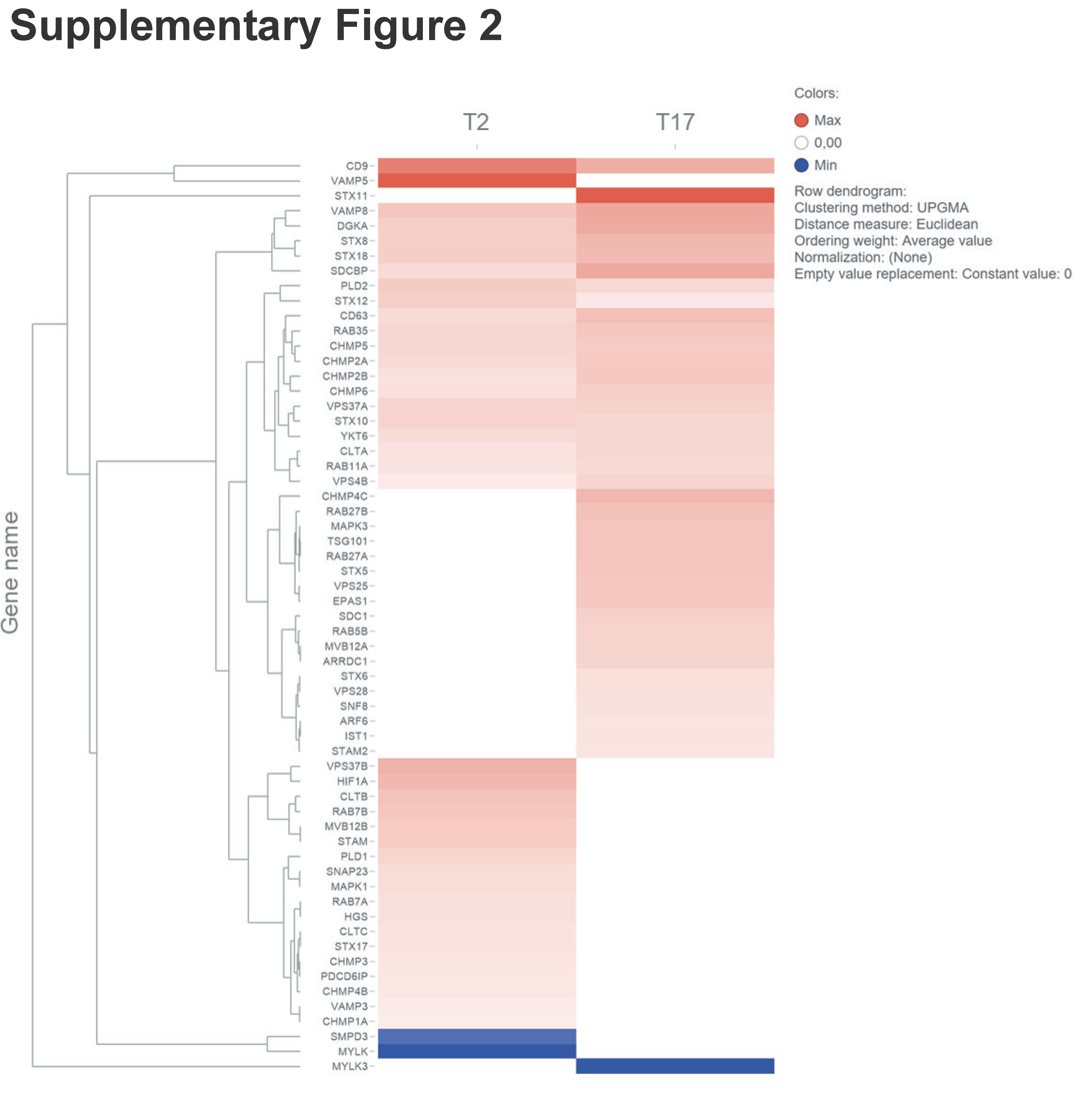

Supplement: Supplementary file 2 — Additional file 2 Supplementary Fig. 2 Genes involved in EV biogenesis, transport, and release are increasingly expressed upon cytokine stimulation. A list of 106 genes corresponding to proteins involved in processes related to EV generation and release was generated based on previous publications and the expression of those found to be differentially expressed by RNAseq (FDR < 0.05) are shown here, along with hierarchical clustering on the gene expression level. [file 12931_2020_1402_MOESM2_ESM.tif]

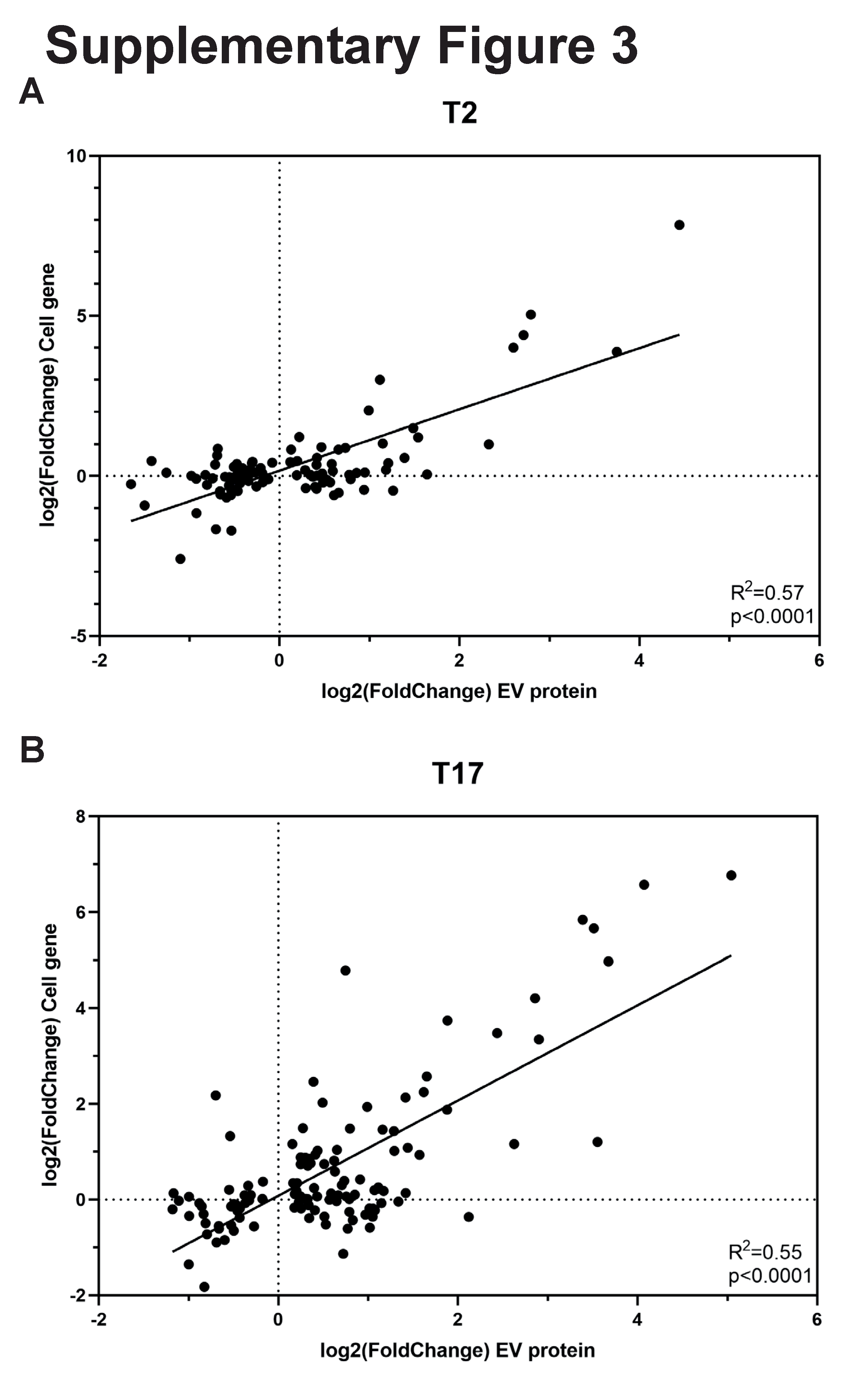

Supplement: Supplementary file 3 — Additional file 3 Supplementary Fig. 3 The airway epithelial EV proteomes correlate with their cellular transcriptomes. Proteins differentially abundant between EVs from T2 (IL-4 + IL-13) and T17 (IL-17A + TNFα) stimulated epithelial cells were correlated to the differential expression of their corresponding gene in cells treated under the same condition. a-b) Plots for T2 stimulation (a) and T17 stimulation (b), fold change for EV proteins on the X-axis and fold change for cellular genes on the Y-axis. All values are log2-transformed and using non-stimulated cells, or EVs from these cells, as control. Solid line corresponds to the interpolated correlation with r2 and p-values as presented in the figures. [file 12931_2020_1402_MOESM3_ESM.tif]
