# Supplementary material for: T2 and T17 cytokines alter the cargo and function of airway epithelium-derived extracellular vesicles
Source: Respir Res. 2020 Jun 19;21:155. doi: 10.1186/s12931-020-01402-3 (PMC7304225; doi:10.1186/s12931-020-01402-3)
Supplement: Supplementary file 4 — Additional file 4 Supplementary Table 1. Top 50 genes induced by T2 stimulation followed by top 50 genes induced by T17 stimulation. [file 12931_2020_1402_MOESM4_ESM.docx]

| Table S1 Top 50 genes induced by T2 stimulation followed by top 50 genes induced by T17 stimulation. | | | | | |
| --- | --- | --- | --- | --- | --- |
| T2 stimulation | | | | | |
| Gene name | **log2FC T2vCtrl** | **p-adj T2vCtrl** | **log2FC T17vCtrl** | **p-adj T17vCtrl** | **EnsemblID** |
| CCL26 | 13,03 | 1,6E-202 | . | . | ENSG00000006606 |
| SH2D1B | 8,82 | 2,4E-110 | 1,38 | 8,2E-03 | ENSG00000198574 |
| NTRK1 | 8,54 | 5,3E-57 | . | . | ENSG00000198400 |
| NOS2 | 7,84 | 2,1E-93 | 2,40 | 1,3E-08 | ENSG00000007171 |
| FETUB | 7,46 | 3,8E-63 | . | . | ENSG00000090512 |
| CAPN14 | 6,98 | 1,1E-69 | . | . | ENSG00000214711 |
| CLDN5 | 6,87 | 1,6E-51 | 1,58 | 4,4E-03 | ENSG00000184113 |
| ANO1 | 6,05 | 1,3E-185 | -0,86 | 3,0E-03 | ENSG00000131620 |
| TREML2 | 5,88 | 5,0E-30 | . | . | ENSG00000112195 |
| ITLN1 | 5,71 | 2,5E-26 | -1,28 | 4,9E-02 | ENSG00000179914 |
| CYTIP | 5,59 | 3,0E-34 | . | . | ENSG00000115165 |
| CISH | 5,58 | 1,3E-98 | -1,76 | 1,5E-05 | ENSG00000114737 |
| SOCS1 | 5,50 | 1,2E-121 | 3,19 | 7,1E-39 | ENSG00000185338 |
| LRRC31 | 5,48 | 2,2E-33 | . | . | ENSG00000114248 |
| CASQ2 | 5,47 | 2,5E-20 | . | . | ENSG00000118729 |
| ALOX15 | 5,37 | 3,8E-52 | -1,89 | 8,2E-07 | ENSG00000161905 |
| OTOGL | 5,37 | 1,0E-29 | . | . | ENSG00000165899 |
| TRABD2A | 5,26 | 2,9E-64 | . | . | ENSG00000186854 |
| CA2 | 5,19 | 1,3E-29 | . | . | ENSG00000104267 |
| SLC26A4 | 5,04 | 6,2E-33 | 6,57 | 5,9E-56 | ENSG00000091137 |
| CLDN22 | 4,92 | 6,2E-65 | 1,47 | 1,3E-05 | ENSG00000177300 |
| CDH26 | 4,84 | 3,0E-54 | . | . | ENSG00000124215 |
| RP1-122P22,4 | 4,72 | 4,4E-27 | . | . | ENSG00000268628 |
| DPP4 | 4,39 | 5,2E-37 | -3,00 | 1,7E-15 | ENSG00000197635 |
| LYPD1 | 4,39 | 1,8E-67 | -1,02 | 3,2E-03 | ENSG00000150551 |
| SERPINB10 | 4,37 | 7,8E-14 | . | . | ENSG00000242550 |
| CLLU1OS | 4,22 | 1,0E-14 | 2,86 | 9,1E-07 | ENSG00000205057 |
| SLC26A4-AS1 | 4,20 | 1,0E-27 | 4,41 | 1,7E-30 | ENSG00000233705 |
| OIT3 | 4,17 | 3,1E-11 | . | . | ENSG00000138315 |
| PADI3 | 4,10 | 4,3E-30 | -1,76 | 1,1E-04 | ENSG00000142619 |
| ADRA2C | 4,09 | 4,2E-17 | . | . | ENSG00000184160 |
| SLAMF6P1 | 4,08 | 1,5E-21 | 1,23 | 2,1E-02 | ENSG00000227243 |
| SERPINB2 | 4,00 | 4,9E-79 | -1,16 | 7,6E-07 | ENSG00000197632 |
| LINC01215 | 3,99 | 4,9E-27 | 3,98 | 7,9E-27 | ENSG00000271856 |
| DUOXA2 | 3,94 | 3,8E-21 | 5,66 | 1,3E-43 | ENSG00000140274 |
| HS3ST4 | 3,94 | 3,4E-10 | . | . | ENSG00000182601 |
| POSTN | 3,89 | 4,6E-20 | . | . | ENSG00000133110 |
| PLA2G3 | 3,88 | 9,8E-17 | 4,10 | 8,8E-19 | ENSG00000100078 |
| SUSD2 | 3,88 | 4,2E-27 | . | . | ENSG00000099994 |
| GADD45G | 3,85 | 8,5E-27 | . | . | ENSG00000130222 |
| LINC00626 | 3,84 | 7,6E-13 | 1,44 | 3,1E-02 | ENSG00000225826 |
| VWF | 3,83 | 1,8E-21 | . | . | ENSG00000110799 |
| KCNK3 | 3,74 | 1,6E-26 | . | . | ENSG00000171303 |
| HAS2 | 3,72 | 1,8E-156 | 1,54 | 4,8E-25 | ENSG00000170961 |
| TRPV6 | 3,70 | 7,8E-18 | . | . | ENSG00000165125 |
| SLC24A3 | 3,69 | 8,9E-13 | . | . | ENSG00000185052 |
| IL1R2 | 3,67 | 2,2E-09 | . | . | ENSG00000115590 |
| B3GNT6 | 3,67 | 3,1E-14 | . | . | ENSG00000198488 |
| SLC9A3 | 3,65 | 2,8E-17 | 4,57 | 5,6E-27 | ENSG00000066230 |
| SELP | 3,63 | 8,2E-18 | 1,83 | 5,7E-05 | ENSG00000174175 |
| T17 stimulation | | | | | |
| Gene name | **log2FC T17vCtrl** | **p-adj T17vCtrl** | **log2FC T2vCtrl** | **p-adj T2vCtrl** | **EnsemblID** |
| CSF3 | 6,77 | 3,0E-36 | . | . | ENSG00000108342 |
| DEFB4A | 6,58 | 7,1E-52 | . | . | ENSG00000171711 |
| SLC26A4 | 6,57 | 5,9E-56 | 5,04 | 6,2E-33 | ENSG00000091137 |
| TNIP3 | 6,50 | 9,9E-63 | 2,44 | 6,2E-09 | ENSG00000050730 |
| PI3 | 6,37 | 2,3E-258 | . | . | ENSG00000124102 |
| IL17REL | 6,16 | 2,6E-28 | . | . | ENSG00000188263 |
| CEACAM7 | 5,84 | 9,4E-51 | -1,37 | 3,8E-03 | ENSG00000007306 |
| CCL20 | 5,76 | 3,0E-49 | -1,09 | 2,6E-02 | ENSG00000115009 |
| IL19 | 5,72 | 2,2E-35 | 3,07 | 2,6E-10 | ENSG00000142224 |
| DEFB4B | 5,70 | 3,5E-37 | . | . | ENSG00000177257 |
| DUOXA2 | 5,66 | 1,3E-43 | 3,94 | 3,8E-21 | ENSG00000140274 |
| AWAT2 | 5,64 | 3,0E-29 | . | . | ENSG00000147160 |
| SLC5A8 | 5,33 | 5,3E-33 | . | . | ENSG00000256870 |
| GLYATL2 | 5,11 | 5,4E-60 | 2,66 | 1,9E-15 | ENSG00000156689 |
| SLC5A1 | 4,97 | 2,8E-69 | 1,96 | 9,4E-11 | ENSG00000100170 |
| SELL | 4,86 | 4,9E-41 | . | . | ENSG00000188404 |
| EPS8L3 | 4,78 | 1,2E-21 | . | . | ENSG00000198758 |
| ERMN | 4,78 | 8,8E-15 | 3,00 | 6,8E-06 | ENSG00000136541 |
| LTF | 4,70 | 8,8E-23 | . | . | ENSG00000012223 |
| SLC39A8 | 4,67 | 1,2E-52 | 3,31 | 1,5E-26 | ENSG00000138821 |
| SLC9A3 | 4,57 | 5,6E-27 | 3,65 | 2,8E-17 | ENSG00000066230 |
| FCRL2 | 4,57 | 6,8E-14 | . | . | ENSG00000132704 |
| CD83 | 4,45 | 1,8E-32 | . | . | ENSG00000112149 |
| SLC26A4-AS1 | 4,41 | 1,7E-30 | 4,20 | 1,0E-27 | ENSG00000233705 |
| DDIT4L | 4,40 | 5,4E-22 | . | . | ENSG00000145358 |
| CPXM1 | 4,35 | 1,2E-12 | . | . | ENSG00000088882 |
| TNFAIP6 | 4,34 | 3,7E-22 | 1,23 | 3,2E-02 | ENSG00000123610 |
| EBI3 | 4,30 | 7,1E-20 | . | . | ENSG00000105246 |
| MIR3142HG | 4,20 | 1,6E-18 | . | . | ENSG00000253522 |
| DUOX2 | 4,20 | 3,2E-53 | 3,39 | 1,3E-34 | ENSG00000140279 |
| S100A7 | 4,17 | 1,0E-21 | -2,59 | 1,3E-06 | ENSG00000143556 |
| HP | 4,12 | 8,2E-20 | -1,49 | 7,3E-03 | ENSG00000257017 |
| PLA2G3 | 4,10 | 8,8E-19 | 3,88 | 9,8E-17 | ENSG00000100078 |
| CXCL3 | 4,09 | 5,7E-21 | . | . | ENSG00000163734 |
| IL4I1 | 4,06 | 4,7E-17 | . | . | ENSG00000104951 |
| LINC01215 | 3,98 | 7,9E-27 | 3,99 | 4,9E-27 | ENSG00000271856 |
| RP11-321F8,4 | 3,96 | 5,2E-10 | . | . | ENSG00000257543 |
| CXCL6 | 3,92 | 5,3E-15 | . | . | ENSG00000124875 |
| SLC6A20 | 3,91 | 4,7E-62 | . | . | ENSG00000163817 |
| CXCL1 | 3,88 | 5,7E-35 | . | . | ENSG00000163739 |
| SERPINB7 | 3,88 | 6,9E-25 | . | . | ENSG00000166396 |
| C6orf223 | 3,87 | 9,4E-12 | 1,54 | 2,6E-02 | ENSG00000181577 |
| CXCL8 | 3,82 | 5,7E-15 | . | . | ENSG00000169429 |
| PDZK1IP1 | 3,74 | 6,3E-55 | 1,11 | 3,4E-05 | ENSG00000162366 |
| SOCS2-AS1 | 3,72 | 7,2E-24 | . | . | ENSG00000246985 |
| TNFRSF1B | 3,69 | 3,4E-16 | . | . | ENSG00000028137 |
| PGLYRP4 | 3,68 | 5,8E-29 | -1,92 | 1,2E-05 | ENSG00000163218 |
| RP11-211N11,5 | 3,64 | 3,9E-15 | 1,72 | 1,4E-03 | ENSG00000234393 |
| PRRX2 | 3,64 | 2,6E-50 | . | . | ENSG00000167157 |
| PARM1 | 3,61 | 7,5E-36 | 3,39 | 1,4E-31 | ENSG00000169116 |
| *Definition of abbreviations:* FC = fold change; p-adj = adjusted p-value (Benjamini-Hochberg multiple correction, FDR). | | | | | |
| . Indicates that the gene did not pass an FDR <0.05. | | | | | |
