# Supplementary material for: T2 and T17 cytokines alter the cargo and function of airway epithelium-derived extracellular vesicles
Source: Respir Res. 2020 Jun 19;21:155. doi: 10.1186/s12931-020-01402-3 (PMC7304225; doi:10.1186/s12931-020-01402-3)
Supplement: Supplementary file 5 — Additional file 5 Supplementary Table 2. 1358 proteins identified in EVs from bronchial epithelial cells, with differential abundance in EVs from cells after T2 and T17 stimulation. [file 12931_2020_1402_MOESM5_ESM.docx]

| **Table S2. 1358 proteins identified in EVs from bronchial epithelial cells, with differential abundance in EVs from cells after T2 and T17 stimulation.** | | | | | | | | | |
| --- | --- | --- | --- | --- | --- | --- | --- | --- | --- |
| **Accession ID** | **Description** | **Gene name** | **# Unique Peptides** | **# Peptides** | **MW [kDa]** | **log2FC T17vCtrl** | **p-value T17vCtrl** | **log2FC T2vCtrl** | **p-value T2vCtrl** |
| Q9NRX4 | 14 kDa phosphohistidine phosphatase | PHPT1 | 2 | 2 | 13,8 | 0,02 | 0,807 | 0,07 | 0,703 |
| P31946 | 14-3-3 protein beta/alpha | YWHAB | 3 | 7 | 28,1 | -0,12 | 0,206 | -0,17 | 0,395 |
| P62258 | 14-3-3 protein epsilon | YWHAE | 7 | 8 | 29,2 | -0,03 | 0,978 | -0,35 | 0,291 |
| Q04917 | 14-3-3 protein eta | YWHAH | 8 | 10 | 28,2 | -0,04 | 0,991 | -0,38 | 0,287 |
| P61981 | 14-3-3 protein gamma | YWHAG | 6 | 9 | 28,3 | -0,13 | 0,663 | -0,29 | 0,489 |
| P31947 | 14-3-3 protein sigma | SFN | 6 | 8 | 27,8 | -0,12 | 0,242 | -0,03 | 0,902 |
| P27348 | 14-3-3 protein theta | YWHAQ | 3 | 6 | 27,7 | 0,15 | 0,516 | -0,24 | 0,643 |
| P63104 | 14-3-3 protein zeta/delta | YWHAZ | 5 | 7 | 27,7 | -0,17 | 0,340 | -0,17 | 0,350 |
| Q9C0C2 | 182 kDa tankyrase-1-binding protein | TNKS1BP1 | 3 | 3 | 181,7 | -0,44 | 0,347 | -0,51 | 0,072 |
| Q01970 | 1-phosphatidylinositol 4,5-bisphosphate phosphodiesterase beta-3 | PLCB3 | 8 | 8 | 138,7 | -0,37 | 0,107 | 0,28 | 0,006 |
| Q15147 | 1-phosphatidylinositol 4,5-bisphosphate phosphodiesterase beta-4 | PLCB4 | 5 | 5 | 134,4 | -0,54 | 0,123 | 0,06 | 0,657 |
| P51178 | 1-phosphatidylinositol 4,5-bisphosphate phosphodiesterase delta-1 | PLCD1 | 1 | 1 | 85,6 | -0,53 | 0,030 | -0,02 | 0,733 |
| Q8N3E9 | 1-phosphatidylinositol 4,5-bisphosphate phosphodiesterase delta-3 | PLCD3 | 1 | 1 | 89,2 | -0,18 | 0,796 | 0,79 | 0,036 |
| P09543 | 2',3'-cyclic-nucleotide 3'-phosphodiesterase | CNP | 5 | 5 | 47,5 | -0,26 | 0,235 | -0,10 | 0,162 |
| O43242 | 26S proteasome non-ATPase regulatory subunit 3 | PSMD3 | 1 | 1 | 60,9 | -0,92 | 0,794 | -0,76 | 0,672 |
| O43598 | 2'-deoxynucleoside 5'-phosphate N-hydrolase 1 | DNPH1 | 1 | 1 | 19,1 | 0,33 | 0,027 | 0,04 | 0,727 |
| O95861 | 3'(2'),5'-bisphosphate nucleotidase 1 | BPNT1 | 4 | 4 | 33,4 | 0,00 | 0,902 | 0,17 | 0,535 |
| Q9BUT1 | 3-hydroxybutyrate dehydrogenase type 2 | BDH2 | 1 | 1 | 26,7 | 0,10 | 0,564 | 0,29 | 0,249 |
| P62269 | 40S ribosomal protein S18 | RPS18 | 2 | 2 | 17,7 | -0,19 | 0,814 | 0,38 | 0,453 |
| P62847 | 40S ribosomal protein S24 | RPS24 | 1 | 1 | 15,4 | -0,73 | 0,757 | 0,81 | 0,344 |
| P62851 | 40S ribosomal protein S25 | RPS25 | 1 | 1 | 13,7 | -0,14 | 0,794 | 0,80 | 0,305 |
| P62854 | 40S ribosomal protein S26 | RPS26 | 1 | 1 | 13,0 | -0,44 | 0,643 | 0,89 | 0,220 |
| P62857 | 40S ribosomal protein S28 | RPS28 | 1 | 1 | 7,8 | -2,52 | 0,319 | -1,11 | 0,958 |
| P23396 | 40S ribosomal protein S3 | RPS3 | 3 | 3 | 26,7 | 0,23 | 0,735 | 0,49 | 0,364 |
| P61247 | 40S ribosomal protein S3a | RPS3A | 3 | 3 | 29,9 | -0,96 | 0,340 | 0,57 | 0,368 |
| P46782 | 40S ribosomal protein S5 | RPS5 | 1 | 1 | 22,9 | 0,73 | 0,003 | 0,05 | 0,880 |
| P08865 | 40S ribosomal protein SA | RPSA | 1 | 1 | 32,8 | 1,14 | 0,067 | 0,73 | 0,909 |
| P49189 | 4-trimethylaminobutyraldehyde dehydrogenase | ALDH9A1 | 3 | 3 | 53,8 | 0,29 | 0,194 | -0,02 | 0,868 |
| Q13131 | 5'-AMP-activated protein kinase catalytic subunit alpha-1 | PRKAA1 | 3 | 3 | 64,0 | 0,65 | 0,066 | 0,61 | 0,113 |
| P54619 | 5'-AMP-activated protein kinase subunit gamma-1 | PRKAG1 | 3 | 3 | 37,6 | 0,45 | 0,054 | 0,36 | 0,296 |
| P21589 | 5'-nucleotidase | NT5E | 4 | 4 | 63,3 | -0,99 | 0,045 | -0,23 | 0,205 |
| O14841 | 5-oxoprolinase | OPLAH | 1 | 1 | 137,4 | 0,23 | 0,353 | 0,59 | 0,031 |
| P62906 | 60S ribosomal protein L10a | RPL10A | 1 | 1 | 24,8 | -0,35 | 0,753 | 0,12 | 0,641 |
| P26373 | 60S ribosomal protein L13 | RPL13 | 1 | 1 | 24,2 | -0,19 | 0,725 | 0,82 | 0,064 |
| P61313 | 60S ribosomal protein L15 | RPL15 | 1 | 1 | 24,1 | -0,61 | 0,765 | 0,97 | 0,232 |
| P18621 | 60S ribosomal protein L17 | RPL17 | 3 | 3 | 21,4 | -0,37 | 0,458 | 0,86 | 0,316 |
| Q02543 | 60S ribosomal protein L18a | RPL18A | 1 | 1 | 20,7 | -0,27 | 0,469 | 0,83 | 0,265 |
| P83731 | 60S ribosomal protein L24 | RPL24 | 1 | 1 | 17,8 | -0,67 | 0,484 | 1,03 | 0,321 |
| P61254 | 60S ribosomal protein L26 | RPL26 | 1 | 1 | 17,2 | -0,51 | 0,268 | 0,05 | 0,787 |
| P46776 | 60S ribosomal protein L27a | RPL27A | 1 | 1 | 16,6 | -0,68 | 0,918 | 0,31 | 0,521 |
| P46779 | 60S ribosomal protein L28 | RPL28 | 2 | 2 | 15,7 | -0,09 | 0,937 | 0,71 | 0,332 |
| P39023 | 60S ribosomal protein L3 | RPL3 | 1 | 1 | 46,1 | -0,14 | 0,758 | 1,30 | 0,212 |
| P62899 | 60S ribosomal protein L31 | RPL31 | 2 | 2 | 14,5 | 0,01 | 0,692 | 0,54 | 0,406 |
| Q02878 | 60S ribosomal protein L6 | RPL6 | 1 | 1 | 32,7 | -0,73 | 0,603 | 0,59 | 0,488 |
| O60825 | 6-phosphofructo-2-kinase/fructose-2,6-bisphosphatase 2 | PFKFB2 | 1 | 1 | 58,4 | 0,46 | 0,304 | -0,30 | 0,425 |
| P52209 | 6-phosphogluconate dehydrogenase, decarboxylating | PGD | 5 | 5 | 53,1 | 0,13 | 0,265 | 0,14 | 0,485 |
| O95336 | 6-phosphogluconolactonase | PGLS | 1 | 1 | 27,5 | 0,10 | 0,599 | -0,09 | 0,844 |
| P11021 | 78 kDa glucose-regulated protein | HSPA5 | 2 | 4 | 72,3 | 0,63 | 0,347 | 0,45 | 0,170 |
| Q7L2J0 | 7SK snRNA methylphosphate capping enzyme | MEPCE | 1 | 1 | 74,3 | -0,44 | 0,243 | -0,45 | 0,270 |
| Q8IZP0 | Abl interactor 1 | ABI1 | 1 | 1 | 55,0 | -1,49 | 0,333 | -1,22 | 0,161 |
| Q86V21 | Acetoacetyl-CoA synthetase | AACS | 1 | 1 | 75,1 | 0,37 | 0,201 | 0,37 | 0,820 |
| Q9BWD1 | Acetyl-CoA acetyltransferase, cytosolic | ACAT2 | 1 | 1 | 41,3 | -0,75 | 0,373 | -0,79 | 0,342 |
| Q9NR19 | Acetyl-coenzyme A synthetase, cytoplasmic | ACSS2 | 1 | 1 | 78,5 | -0,27 | 0,869 | -0,29 | 0,752 |
| Q92485 | Acid sphingomyelinase-like phosphodiesterase 3b | SMPDL3B | 3 | 3 | 50,8 | -0,39 | 0,067 | 0,01 | 0,801 |
| P60709 | Actin, cytoplasmic 1 | ACTB | 10 | 10 | 41,7 | -0,04 | 0,921 | -0,13 | 0,873 |
| P61160 | Actin-related protein 2 | ACTR2 | 1 | 1 | 44,7 | 0,04 | 0,645 | -0,27 | 0,949 |
| Q92747 | Actin-related protein 2/3 complex subunit 1A | ARPC1A | 1 | 1 | 41,5 | -0,09 | 0,786 | 0,10 | 0,971 |
| O15143 | Actin-related protein 2/3 complex subunit 1B | ARPC1B | 2 | 2 | 40,9 | -0,08 | 0,892 | 0,12 | 0,468 |
| O15144 | Actin-related protein 2/3 complex subunit 2 | ARPC2 | 1 | 1 | 34,3 | -0,10 | 0,978 | -0,22 | 0,582 |
| O15145 | Actin-related protein 2/3 complex subunit 3 | ARPC3 | 1 | 1 | 20,5 | 0,09 | 0,712 | -0,17 | 0,768 |
| P59998 | Actin-related protein 2/3 complex subunit 4 | ARPC4 | 1 | 1 | 19,7 | -0,04 | 0,905 | -0,23 | 0,628 |
| O15511 | Actin-related protein 2/3 complex subunit 5 | ARPC5 | 1 | 1 | 16,3 | -0,09 | 0,718 | -0,43 | 0,257 |
| Q9BPX5 | Actin-related protein 2/3 complex subunit 5-like protein | ARPC5L | 2 | 2 | 16,9 | 0,06 | 0,700 | -0,34 | 0,378 |
| P61158 | Actin-related protein 3 | ACTR3 | 2 | 2 | 47,3 | 0,12 | 0,650 | -0,08 | 0,985 |
| O95433 | Activator of 90 kDa heat shock protein ATPase homolog 1 | AHSA1 | 1 | 1 | 38,3 | -0,28 | 0,836 | -0,23 | 0,538 |
| Q12979 | Active breakpoint cluster region-related protein | ABR | 4 | 4 | 97,5 | -0,02 | 0,876 | 0,31 | 0,065 |
| P13798 | Acylamino-acid-releasing enzyme | APEH | 2 | 2 | 81,2 | 0,57 | 0,416 | -0,35 | 0,735 |
| P07311 | Acylphosphatase-1 | ACYP1 | 1 | 1 | 11,3 | 0,58 | 0,355 | -0,39 | 0,700 |
| O75608 | Acyl-protein thioesterase 1 | LYPLA1 | 2 | 2 | 24,7 | -0,05 | 0,982 | -0,10 | 0,771 |
| P46108 | Adapter molecule crk | CRK | 4 | 4 | 33,8 | 0,35 | 0,272 | 0,10 | 0,485 |
| Q9NVZ3 | Adaptin ear-binding coat-associated protein 2 | NECAP2 | 1 | 1 | 28,3 | -0,11 | 0,571 | -0,44 | 0,146 |
| P07741 | Adenine phosphoribosyltransferase | APRT | 2 | 2 | 19,6 | 0,40 | 0,177 | 0,01 | 0,888 |
| P23526 | Adenosylhomocysteinase | AHCY | 3 | 3 | 47,7 | 0,02 | 0,895 | 0,20 | 0,201 |
| Q96MA6 | Adenylate kinase 8 | AK8 | 2 | 2 | 54,9 | -0,35 | 0,463 | -0,12 | 0,701 |
| P00568 | Adenylate kinase isoenzyme 1 | AK1 | 4 | 4 | 21,6 | -0,08 | 0,621 | -0,01 | 0,859 |
| P30566 | Adenylosuccinate lyase | ADSL | 1 | 1 | 54,9 | 0,54 | 0,119 | 0,45 | 0,482 |
| P30520 | Adenylosuccinate synthetase isozyme 2 | ADSS | 1 | 1 | 50,1 | 0,46 | 0,422 | 0,86 | 0,147 |
| Q01518 | Adenylyl cyclase-associated protein 1 | CAP1 | 6 | 6 | 51,9 | 0,16 | 0,035 | 0,57 | 0,129 |
| P40123 | Adenylyl cyclase-associated protein 2 | CAP2 | 1 | 1 | 52,8 | -0,10 | 0,947 | 0,35 | 0,749 |
| Q5T601 | Adhesion G-protein coupled receptor F1 | ADGRF1 | 2 | 2 | 101,3 | 0,27 | 0,211 | -0,73 | 0,082 |
| Q86SQ4 | Adhesion G-protein coupled receptor G6 | ADGRG6 | 5 | 5 | 136,6 | -0,44 | 0,317 | -0,27 | 0,057 |
| Q10588 | ADP-ribosyl cyclase/cyclic ADP-ribose hydrolase 2 | BST1 | 1 | 1 | 35,7 | -0,36 | 0,087 | -0,48 | 0,017 |
| P61204 | ADP-ribosylation factor 3 | ARF3 | 1 | 1 | 20,6 | 0,13 | 0,499 | -0,08 | 0,895 |
| P18085 | ADP-ribosylation factor 4 | ARF4 | 1 | 1 | 20,5 | 0,03 | 0,891 | -0,13 | 0,521 |
| P84085 | ADP-ribosylation factor 5 | ARF5 | 1 | 1 | 20,5 | -0,04 | 0,775 | -0,19 | 0,309 |
| Q9NP61 | ADP-ribosylation factor GTPase-activating protein 3 | ARFGAP3 | 1 | 1 | 56,9 | 0,14 | 0,482 | 0,33 | 0,276 |
| P36405 | ADP-ribosylation factor-like protein 3 | ARL3 | 1 | 1 | 20,4 | -0,12 | 0,864 | -0,37 | 0,059 |
| Q9H0F7 | ADP-ribosylation factor-like protein 6 | ARL6 | 1 | 1 | 21,1 | -0,06 | 0,931 | -0,35 | 0,034 |
| Q9NVJ2 | ADP-ribosylation factor-like protein 8B | ARL8B | 1 | 1 | 21,5 | 1,15 | 0,032 | 0,13 | 0,707 |
| Q9UKK9 | ADP-sugar pyrophosphatase | NUDT5 | 1 | 1 | 24,3 | 0,54 | 0,524 | 0,60 | 0,346 |
| P55196 | Afadin | AFDN | 5 | 5 | 206,7 | -0,12 | 0,640 | -0,56 | 0,025 |
| O00468 | Agrin | AGRN | 7 | 7 | 217,1 | 0,01 | 0,973 | 0,57 | 0,103 |
| P49588 | Alanine--tRNA ligase, cytoplasmic | AARS | 2 | 2 | 106,7 | 0,55 | 0,300 | 0,92 | 0,098 |
| P14550 | Alcohol dehydrogenase [NADP(+)] | AKR1A1 | 1 | 1 | 36,5 | 0,25 | 0,017 | 0,05 | 0,910 |
| P00326 | Alcohol dehydrogenase 1C | ADH1C | 6 | 6 | 39,8 | 0,27 | 0,238 | 1,12 | 0,021 |
| P40394 | Alcohol dehydrogenase class 4 mu/sigma chain | ADH7 | 2 | 2 | 41,5 | 0,09 | 0,374 | 0,27 | 0,212 |
| P11766 | Alcohol dehydrogenase class-3 | ADH5 | 2 | 2 | 39,7 | 0,05 | 0,799 | 0,11 | 0,618 |
| P47895 | Aldehyde dehydrogenase family 1 member A3 | ALDH1A3 | 2 | 3 | 56,1 | 0,12 | 0,491 | 0,61 | 0,260 |
| P43353 | Aldehyde dehydrogenase family 3 member B1 | ALDH3B1 | 4 | 4 | 51,8 | -0,40 | 0,633 | -0,49 | 0,092 |
| P30838 | Aldehyde dehydrogenase, dimeric NADP-preferring | ALDH3A1 | 4 | 4 | 50,4 | 0,03 | 0,475 | -0,02 | 0,643 |
| O60218 | Aldo-keto reductase family 1 member B10 | AKR1B10 | 1 | 1 | 36,0 | 1,58 | 0,112 | 0,98 | 0,114 |
| Q04828 | Aldo-keto reductase family 1 member C1 | AKR1C1 | 1 | 3 | 36,8 | 0,13 | 0,437 | 0,07 | 0,114 |
| P42330 | Aldo-keto reductase family 1 member C3 | AKR1C3 | 1 | 3 | 36,8 | 0,16 | 0,504 | 0,19 | 0,072 |
| P05186 | Alkaline phosphatase, tissue-nonspecific isozyme | ALPL | 7 | 7 | 57,3 | 2,90 | 0,006 | 1,39 | 0,009 |
| P51993 | Alpha-(1,3)-fucosyltransferase 6 | FUT6 | 1 | 1 | 41,8 | -0,44 | 0,667 | -1,35 | 0,148 |
| P01009 | Alpha-1-antitrypsin | SERPINA1 | 2 | 2 | 46,7 | -0,17 | 0,935 | 0,02 | 0,709 |
| A8K2U0 | Alpha-2-macroglobulin-like protein 1 | A2ML1 | 1 | 1 | 161,0 | 0,16 | 0,986 | -0,29 | 0,525 |
| P12814 | Alpha-actinin-1 | ACTN1 | 2 | 5 | 103,0 | 0,85 | 0,154 | 0,15 | 0,616 |
| O43707 | Alpha-actinin-4 | ACTN4 | 7 | 10 | 104,8 | 0,32 | 0,385 | -0,01 | 0,762 |
| P49419 | Alpha-aminoadipic semialdehyde dehydrogenase | ALDH7A1 | 1 | 1 | 58,5 | 0,13 | 0,228 | 0,08 | 0,919 |
| P06733 | Alpha-enolase | ENO1 | 8 | 8 | 47,1 | -0,04 | 0,869 | 0,16 | 0,130 |
| P54920 | Alpha-soluble NSF attachment protein | NAPA | 2 | 2 | 33,2 | -0,04 | 0,919 | -0,14 | 0,716 |
| Q9H4A4 | Aminopeptidase B | RNPEP | 3 | 3 | 72,5 | 0,31 | 0,438 | 0,08 | 0,830 |
| P15144 | Aminopeptidase N | ANPEP | 8 | 8 | 109,5 | -0,78 | 0,186 | -0,39 | 0,810 |
| P12821 | Angiotensin-converting enzyme | ACE | 2 | 2 | 149,6 | -1,06 | 0,096 | -0,47 | 0,350 |
| A6QL63 | Ankyrin repeat and BTB/POZ domain-containing protein BTBD11 | BTBD11 | 1 | 1 | 120,8 | -0,08 | 0,805 | -0,32 | 0,477 |
| Q92625 | Ankyrin repeat and SAM domain-containing protein 1A | ANKS1A | 2 | 2 | 123,0 | 0,83 | 0,009 | 0,35 | 0,141 |
| Q9H8Y5 | Ankyrin repeat and zinc finger domain-containing protein 1 | ANKZF1 | 1 | 1 | 80,9 | 2,13 | 0,349 | 1,43 | 0,179 |
| P04083 | Annexin A1 | ANXA1 | 6 | 6 | 38,7 | 0,15 | 0,288 | 0,22 | 0,412 |
| P50995 | Annexin A11 | ANXA11 | 7 | 7 | 54,4 | -0,05 | 0,543 | 0,24 | 0,097 |
| P07355 | Annexin A2 | ANXA2 | 9 | 9 | 38,6 | 0,07 | 0,506 | 0,05 | 0,573 |
| P12429 | Annexin A3 | ANXA3 | 6 | 6 | 36,4 | -0,01 | 0,932 | 0,24 | 0,260 |
| P09525 | Annexin A4 | ANXA4 | 10 | 11 | 35,9 | 0,11 | 0,320 | -0,01 | 0,953 |
| P08758 | Annexin A5 | ANXA5 | 3 | 4 | 35,9 | -0,06 | 0,725 | 0,14 | 0,768 |
| P08133 | Annexin A6 | ANXA6 | 1 | 1 | 75,8 | -0,41 | 0,202 | -0,27 | 0,176 |
| P20073 | Annexin A7 | ANXA7 | 7 | 7 | 52,7 | -0,01 | 0,758 | -0,01 | 0,713 |
| Q4KMQ2 | Anoctamin-6 | ANO6 | 2 | 2 | 106,1 | -0,06 | 0,564 | 0,02 | 0,819 |
| P03973 | Antileukoproteinase | SLPI | 1 | 1 | 14,3 | 0,51 | 0,011 | 1,64 | 0,024 |
| O95782 | AP-2 complex subunit alpha-1 | AP2A1 | 2 | 2 | 107,5 | 0,22 | 0,463 | -0,09 | 0,835 |
| P63010 | AP-2 complex subunit beta | AP2B1 | 2 | 2 | 104,5 | 0,23 | 0,018 | 0,08 | 0,363 |
| Q96CW1 | AP-2 complex subunit mu | AP2M1 | 2 | 2 | 49,6 | -0,33 | 0,661 | -0,26 | 0,741 |
| P53680 | AP-2 complex subunit sigma | AP2S1 | 1 | 1 | 17,0 | 0,66 | 0,111 | -0,51 | 0,196 |
| Q2M2I8 | AP2-associated protein kinase 1 | AAK1 | 1 | 1 | 103,8 | -0,32 | 0,425 | -0,11 | 0,602 |
| P05090 | Apolipoprotein D | APOD | 2 | 2 | 21,3 | 0,54 | 0,079 | 0,03 | 0,919 |
| P02649 | Apolipoprotein E | APOE | 2 | 2 | 36,1 | -0,31 | 0,110 | 0,54 | 0,211 |
| Q07812 | Apoptosis regulator BAX | BAX | 2 | 2 | 21,2 | 0,04 | 0,977 | -0,22 | 0,150 |
| Q9ULZ3 | Apoptosis-associated speck-like protein containing a CARD | PYCARD | 2 | 2 | 21,6 | -0,07 | 0,460 | -0,27 | 0,456 |
| P55064 | Aquaporin-5 | AQP5 | 1 | 1 | 28,3 | -0,69 | 0,001 | -0,54 | 0,008 |
| P16050 | Arachidonate 15-lipoxygenase | ALOX15 | 7 | 7 | 74,8 | -0,18 | 0,123 | 1,92 | 0,069 |
| Q15057 | Arf-GAP with coiled-coil, ANK repeat and PH domain-containing protein 2 | ACAP2 | 1 | 1 | 88,0 | 0,34 | 0,446 | -0,06 | 0,984 |
| P05089 | Arginase-1 | ARG1 | 2 | 2 | 34,7 | 0,69 | 0,908 | 0,51 | 0,556 |
| P54136 | Arginine--tRNA ligase, cytoplasmic | RARS | 1 | 1 | 75,3 | 0,25 | 0,326 | -0,08 | 0,579 |
| P00966 | Argininosuccinate synthase | ASS1 | 2 | 2 | 46,5 | 0,41 | 0,023 | 0,42 | 0,163 |
| Q5W041 | Armadillo repeat-containing protein 3 | ARMC3 | 1 | 1 | 96,3 | 0,03 | 0,610 | 0,00 | 0,808 |
| Q7Z6K5 | Arpin | ARPIN | 1 | 1 | 24,9 | -0,11 | 0,808 | -0,02 | 0,904 |
| Q8N5I2 | Arrestin domain-containing protein 1 | ARRDC1 | 3 | 3 | 46,0 | -0,17 | 0,014 | -0,04 | 0,687 |
| O43776 | Asparagine--tRNA ligase, cytoplasmic | NARS | 2 | 2 | 62,9 | -0,13 | 0,640 | -0,06 | 0,854 |
| P17174 | Aspartate aminotransferase, cytoplasmic | GOT1 | 1 | 1 | 46,2 | 0,03 | 0,369 | 0,20 | 0,072 |
| P14868 | Aspartate--tRNA ligase, cytoplasmic | DARS | 1 | 1 | 57,1 | 0,71 | 0,012 | 0,46 | 0,664 |
| Q9ULA0 | Aspartyl aminopeptidase | DNPEP | 1 | 1 | 52,4 | -1,84 | 0,285 | -1,66 | 0,227 |
| Q15121 | Astrocytic phosphoprotein PEA-15 | PEA15 | 1 | 1 | 15,0 | -1,22 | 0,235 | -0,96 | 0,411 |
| P05496 | ATP synthase F(0) complex subunit C1, mitochondrial | ATP5G1 | 1 | 1 | 14,3 | -0,40 | 0,415 | 0,23 | 0,657 |
| P36542 | ATP synthase subunit gamma, mitochondrial | ATP5C1 | 1 | 1 | 33,0 | -0,43 | 0,114 | 0,66 | 0,187 |
| Q86UK0 | ATP-binding cassette sub-family A member 12 | ABCA12 | 5 | 5 | 293,0 | 1,06 | 0,068 | -0,44 | 0,064 |
| Q86UQ4 | ATP-binding cassette sub-family A member 13 | ABCA13 | 17 | 17 | 575,8 | 1,42 | 0,037 | 0,48 | 0,027 |
| Q9NUQ8 | ATP-binding cassette sub-family F member 3 | ABCF3 | 1 | 1 | 79,7 | 0,36 | 0,267 | -0,41 | 0,311 |
| P53396 | ATP-citrate synthase | ACLY | 1 | 1 | 120,8 | 0,83 | 0,238 | 1,21 | 0,002 |
| P08237 | ATP-dependent 6-phosphofructokinase, muscle type | PFKM | 2 | 2 | 85,1 | 0,79 | 0,006 | 0,93 | 0,184 |
| Q08211 | ATP-dependent RNA helicase A | DHX9 | 1 | 1 | 140,9 | -0,58 | 0,371 | 0,57 | 0,317 |
| Q92499 | ATP-dependent RNA helicase DDX1 | DDX1 | 3 | 3 | 82,4 | -0,30 | 0,291 | 0,20 | 0,261 |
| Q9NUU7 | ATP-dependent RNA helicase DDX19A | DDX19A | 1 | 1 | 53,9 | -0,64 | 0,158 | -0,06 | 0,908 |
| O00148 | ATP-dependent RNA helicase DDX39A | DDX39A | 1 | 1 | 49,1 | 0,54 | 0,683 | 0,77 | 0,156 |
| O00571 | ATP-dependent RNA helicase DDX3X | DDX3X | 3 | 3 | 73,2 | 0,08 | 0,843 | 0,37 | 0,050 |
| O75882 | Attractin | ATRN | 3 | 3 | 158,4 | -0,02 | 0,937 | 0,07 | 0,142 |
| O14645 | Axonemal dynein light intermediate polypeptide 1 | DNALI1 | 5 | 5 | 29,6 | 0,09 | 0,544 | -0,46 | 0,001 |
| O95817 | BAG family molecular chaperone regulator 3 | BAG3 | 1 | 1 | 61,6 | 0,13 | 0,489 | 0,74 | 0,418 |
| Q9HCS5 | Band 4.1-like protein 4A | EPB41L4A | 1 | 1 | 79,0 | -0,90 | 0,218 | -0,61 | 0,383 |
| Q8NFJ9 | Bardet-Biedl syndrome 1 protein | BBS1 | 2 | 2 | 65,0 | 0,33 | 0,248 | -0,48 | 0,913 |
| Q9BXC9 | Bardet-Biedl syndrome 2 protein | BBS2 | 3 | 3 | 79,8 | 0,21 | 0,249 | -0,01 | 0,732 |
| Q96RK4 | Bardet-Biedl syndrome 4 protein | BBS4 | 1 | 1 | 58,2 | 0,30 | 0,127 | -0,26 | 0,007 |
| Q8IWZ6 | Bardet-Biedl syndrome 7 protein | BBS7 | 3 | 3 | 80,3 | 0,12 | 0,368 | -0,69 | 0,555 |
| P98160 | Basement membrane-specific heparan sulfate proteoglycan core protein | HSPG2 | 24 | 24 | 468,5 | 0,76 | 0,100 | 0,53 | 0,003 |
| Q7L1Q6 | Basic leucine zipper and W2 domain-containing protein 1 | BZW1 | 2 | 2 | 48,0 | 0,48 | 0,152 | -0,26 | 0,536 |
| P35613 | Basigin | BSG | 1 | 1 | 42,2 | 0,01 | 0,810 | -0,11 | 0,909 |
| O95395 | Beta-1,3-galactosyl-O-glycosyl-glycoprotein beta-1,6-N-acetylglucosaminyltransferase 3 | GCNT3 | 1 | 1 | 50,8 | 0,00 | 0,719 | 0,57 | 0,153 |
| O60513 | Beta-1,4-galactosyltransferase 4 | B4GALT4 | 1 | 1 | 40,0 | 1,13 | 0,198 | 0,20 | 0,572 |
| O00462 | Beta-mannosidase | MANBA | 2 | 2 | 100,8 | 0,46 | 0,414 | -0,29 | 0,849 |
| P31939 | Bifunctional purine biosynthesis protein PURH | ATIC | 1 | 1 | 64,6 | -0,03 | 0,671 | 0,10 | 0,438 |
| P53004 | Biliverdin reductase A | BLVRA | 2 | 2 | 33,4 | 0,05 | 0,631 | -0,09 | 0,610 |
| Q10589 | Bone marrow stromal antigen 2 | BST2 | 2 | 2 | 19,8 | -0,38 | 0,424 | -0,25 | 0,723 |
| P12645 | Bone morphogenetic protein 3 | BMP3 | 2 | 2 | 53,3 | 0,64 | 0,097 | -0,02 | 0,791 |
| Q8TDL5 | BPI fold-containing family B member 1 | BPIFB1 | 4 | 4 | 52,4 | 0,24 | 0,480 | -0,01 | 0,967 |
| P80723 | Brain acid soluble protein 1 | BASP1 | 8 | 8 | 22,7 | -0,23 | 0,535 | -0,39 | 0,130 |
| Q9UQB8 | Brain-specific angiogenesis inhibitor 1-associated protein 2 | BAIAP2 | 12 | 12 | 60,8 | -0,66 | 0,038 | -0,39 | 0,004 |
| Q9UHR4 | Brain-specific angiogenesis inhibitor 1-associated protein 2-like protein 1 | BAIAP2L1 | 10 | 10 | 56,8 | -0,37 | 0,392 | -0,81 | 0,031 |
| Q9GZN4 | Brain-specific serine protease 4 | PRSS22 | 2 | 2 | 33,7 | 1,16 | 0,035 | 0,15 | 0,490 |
| P11274 | Breakpoint cluster region protein | BCR | 1 | 1 | 142,7 | -0,15 | 0,252 | -0,20 | 0,220 |
| O75363 | Breast carcinoma-amplified sequence 1 | BCAS1 | 5 | 5 | 61,7 | -0,59 | 0,169 | -0,70 | 0,063 |
| Q9NQY0 | Bridging integrator 3 | BIN3 | 1 | 1 | 29,6 | -0,20 | 0,517 | 0,29 | 0,163 |
| Q5VW32 | BRO1 domain-containing protein BROX | BROX | 3 | 3 | 46,4 | 0,54 | 0,174 | 0,64 | 0,132 |
| Q96CX2 | BTB/POZ domain-containing protein KCTD12 | KCTD12 | 1 | 1 | 35,7 | -0,20 | 0,825 | -0,20 | 0,807 |
| P11586 | C-1-tetrahydrofolate synthase, cytoplasmic | MTHFD1 | 2 | 2 | 101,5 | 0,17 | 0,534 | -0,24 | 0,606 |
| P12830 | Cadherin-1 | CDH1 | 1 | 1 | 97,4 | 0,40 | 0,304 | -0,10 | 0,815 |
| P55290 | Cadherin-13 | CDH13 | 1 | 1 | 78,2 | -0,42 | 0,413 | 0,12 | 0,729 |
| Q6ZTQ4 | Cadherin-related family member 3 | CDHR3 | 4 | 4 | 97,9 | -0,23 | 0,987 | -0,36 | 0,180 |
| A6H8M9 | Cadherin-related family member 4 | CDHR4 | 4 | 4 | 85,8 | -0,17 | 0,598 | -0,22 | 0,850 |
| Q99828 | Calcium and integrin-binding protein 1 | CIB1 | 5 | 5 | 21,7 | 0,06 | 0,693 | -0,69 | 0,006 |
| Q13557 | Calcium/calmodulin-dependent protein kinase type II subunit delta | CAMK2D | 2 | 2 | 56,3 | 0,62 | 0,156 | 0,65 | 0,046 |
| Q14CN2 | Calcium-activated chloride channel regulator 4 | CLCA4 | 2 | 2 | 101,2 | 0,42 | 0,224 | -0,04 | 0,801 |
| Q9Y376 | Calcium-binding protein 39 | CAB39 | 2 | 2 | 39,8 | 0,06 | 0,689 | 0,06 | 0,642 |
| Q86UW7 | Calcium-dependent secretion activator 2 | CADPS2 | 1 | 1 | 147,6 | -1,50 | 0,062 | -1,42 | 0,045 |
| Q9Y2V2 | Calcium-regulated heat-stable protein 1 | CARHSP1 | 1 | 1 | 15,9 | 0,06 | 0,559 | -0,16 | 0,573 |
| Q13938 | Calcyphosin | CAPS | 4 | 4 | 21,0 | 0,16 | 0,347 | -0,31 | 0,484 |
| P62158 | Calmodulin | CALM1 | 2 | 2 | 16,8 | -0,11 | 0,107 | -0,39 | 0,311 |
| Q9NZT1 | Calmodulin-like protein 5 | CALML5 | 1 | 1 | 15,9 | 0,30 | 0,913 | 1,25 | 0,359 |
| P04632 | Calpain small subunit 1 | CAPNS1 | 2 | 2 | 28,3 | 0,17 | 0,377 | 0,21 | 0,826 |
| P07384 | Calpain-1 catalytic subunit | CAPN1 | 5 | 6 | 81,8 | 0,11 | 0,295 | 0,05 | 0,838 |
| Q6MZZ7 | Calpain-13 | CAPN13 | 1 | 1 | 76,6 | -0,49 | 0,394 | -0,07 | 0,522 |
| P17655 | Calpain-2 catalytic subunit | CAPN2 | 3 | 4 | 79,9 | 0,10 | 0,585 | -0,16 | 0,100 |
| O15484 | Calpain-5 | CAPN5 | 5 | 5 | 73,1 | -0,60 | 0,010 | -0,11 | 0,440 |
| Q9Y6W3 | Calpain-7 | CAPN7 | 2 | 2 | 92,6 | -0,15 | 0,811 | -0,39 | 0,504 |
| O14815 | Calpain-9 | CAPN9 | 1 | 2 | 79,0 | 0,00 | 0,524 | -0,62 | 0,401 |
| P20810 | Calpastatin | CAST | 3 | 3 | 76,5 | 0,02 | 0,682 | 0,24 | 0,312 |
| Q99439 | Calponin-2 | CNN2 | 3 | 3 | 33,7 | 0,06 | 0,792 | -0,22 | 0,395 |
| P27797 | Calreticulin | CALR | 1 | 1 | 48,1 | 0,59 | 0,131 | -0,18 | 0,217 |
| P13861 | cAMP-dependent protein kinase type II-alpha regulatory subunit | PRKAR2A | 4 | 4 | 45,5 | -0,10 | 0,613 | 0,03 | 0,930 |
| Q8NCG5 | Carbohydrate sulfotransferase 4 | CHST4 | 1 | 1 | 45,1 | 0,48 | 0,523 | -0,11 | 0,436 |
| P16152 | Carbonyl reductase [NADPH] 1 | CBR1 | 4 | 4 | 30,4 | 0,07 | 0,517 | -0,09 | 0,787 |
| O75976 | Carboxypeptidase D | CPD | 3 | 3 | 152,8 | 0,21 | 0,386 | -0,12 | 0,460 |
| P13688 | Carcinoembryonic antigen-related cell adhesion molecule 1 | CEACAM1 | 1 | 1 | 57,5 | -0,15 | 0,937 | -0,42 | 0,103 |
| Q7Z692 | Carcinoembryonic antigen-related cell adhesion molecule 19 | CEACAM19 | 1 | 1 | 32,6 | 2,09 | 0,100 | 0,77 | 0,051 |
| P06731 | Carcinoembryonic antigen-related cell adhesion molecule 5 | CEACAM5 | 2 | 2 | 76,7 | 0,44 | 0,302 | 0,94 | 0,023 |
| P40199 | Carcinoembryonic antigen-related cell adhesion molecule 6 | CEACAM6 | 1 | 1 | 37,2 | 0,51 | 0,676 | 0,21 | 0,795 |
| Q14002 | Carcinoembryonic antigen-related cell adhesion molecule 7 | CEACAM7 | 3 | 3 | 29,4 | 3,39 | 0,014 | 0,15 | 0,548 |
| P48729 | Casein kinase I isoform alpha | CSNK1A1 | 1 | 1 | 38,9 | -0,75 | 0,060 | -0,72 | 0,034 |
| Q9Y6M4 | Casein kinase I isoform gamma-3 | CSNK1G3 | 3 | 3 | 51,4 | -0,28 | 0,633 | -0,05 | 0,917 |
| P31944 | Caspase-14 | CASP14 | 2 | 2 | 27,7 | -0,65 | 0,372 | 0,23 | 0,654 |
| P42574 | Caspase-3 | CASP3 | 2 | 2 | 31,6 | -0,10 | 0,147 | -0,09 | 0,658 |
| P55210 | Caspase-7 | CASP7 | 1 | 1 | 34,3 | -0,05 | 0,966 | -0,88 | 0,327 |
| P04040 | Catalase | CAT | 3 | 3 | 59,7 | 0,63 | 0,575 | 0,37 | 0,595 |
| P21964 | Catechol O-methyltransferase | COMT | 2 | 2 | 30,0 | -0,24 | 0,403 | -0,18 | 0,534 |
| P35221 | Catenin alpha-1 | CTNNA1 | 4 | 4 | 100,0 | -0,33 | 0,235 | 0,22 | 0,544 |
| P07339 | Cathepsin D | CTSD | 3 | 3 | 44,5 | 0,53 | 0,019 | 0,56 | 0,063 |
| Q6YHK3 | CD109 antigen | CD109 | 2 | 2 | 161,6 | 0,16 | 0,310 | 0,02 | 0,782 |
| P48509 | CD151 antigen | CD151 | 1 | 1 | 28,3 | -0,75 | 0,252 | -0,61 | 0,164 |
| Q9Y5K6 | CD2-associated protein | CD2AP | 4 | 4 | 71,4 | 0,65 | 0,007 | 0,06 | 0,884 |
| P16070 | CD44 antigen | CD44 | 4 | 4 | 81,5 | -0,19 | 0,983 | 1,54 | 0,020 |
| P13987 | CD59 glycoprotein | CD59 | 1 | 1 | 14,2 | -0,29 | 0,619 | -0,90 | 0,130 |
| P08962 | CD63 antigen | CD63 | 2 | 2 | 25,6 | -0,04 | 0,777 | -0,38 | 0,185 |
| P60033 | CD81 antigen | CD81 | 2 | 2 | 25,8 | -0,42 | 0,986 | -0,36 | 0,952 |
| P27701 | CD82 antigen | CD82 | 2 | 2 | 29,6 | -0,20 | 0,456 | -0,30 | 0,490 |
| P21926 | CD9 antigen | CD9 | 2 | 2 | 25,4 | 0,04 | 0,681 | 0,37 | 0,136 |
| Q15642 | Cdc42-interacting protein 4 | TRIP10 | 3 | 3 | 68,3 | -0,16 | 0,120 | -0,03 | 0,913 |
| Q9NV96 | Cell cycle control protein 50A | TMEM30A | 1 | 1 | 40,7 | 0,07 | 0,577 | -0,29 | 0,479 |
| Q3MIR4 | Cell cycle control protein 50B | TMEM30B | 2 | 2 | 38,9 | -0,11 | 0,528 | 0,04 | 0,982 |
| P60953 | Cell division control protein 42 homolog | CDC42 | 2 | 2 | 21,2 | -0,25 | 0,177 | -0,17 | 0,168 |
| P29373 | Cellular retinoic acid-binding protein 2 | CRABP2 | 2 | 2 | 15,7 | -0,43 | 0,003 | -0,25 | 0,652 |
| Q9BYV8 | Centrosomal protein of 41 kDa | CEP41 | 1 | 1 | 41,3 | -0,02 | 0,969 | 0,00 | 0,764 |
| Q96ST8 | Centrosomal protein of 89 kDa | CEP89 | 2 | 2 | 89,5 | -0,42 | 0,762 | -0,28 | 0,926 |
| Q49MI3 | Ceramide kinase-like protein | CERKL | 2 | 2 | 62,6 | 0,44 | 0,402 | -0,30 | 0,332 |
| Q9BSQ5 | Cerebral cavernous malformations 2 protein | CCM2 | 3 | 3 | 48,8 | -0,47 | 0,049 | -0,20 | 0,486 |
| P00450 | Ceruloplasmin | CP | 9 | 9 | 122,1 | 0,09 | 0,495 | -0,26 | 0,643 |
| Q9HD42 | Charged multivesicular body protein 1a | CHMP1A | 3 | 3 | 21,7 | 0,06 | 0,650 | 0,26 | 0,110 |
| Q7LBR1 | Charged multivesicular body protein 1b | CHMP1B | 6 | 6 | 22,1 | -0,03 | 0,924 | 0,39 | 0,158 |
| O43633 | Charged multivesicular body protein 2a | CHMP2A | 4 | 4 | 25,1 | -0,04 | 0,858 | 0,58 | 0,047 |
| Q9UQN3 | Charged multivesicular body protein 2b | CHMP2B | 4 | 5 | 23,9 | -0,09 | 0,621 | 0,65 | 0,058 |
| Q9BY43 | Charged multivesicular body protein 4a | CHMP4A | 2 | 2 | 25,1 | 0,10 | 0,540 | 0,29 | 0,023 |
| Q9H444 | Charged multivesicular body protein 4b | CHMP4B | 4 | 4 | 24,9 | 0,05 | 0,580 | 0,39 | 0,122 |
| Q9NZZ3 | Charged multivesicular body protein 5 | CHMP5 | 4 | 4 | 24,6 | -0,07 | 0,877 | 0,28 | 0,307 |
| Q96FZ7 | Charged multivesicular body protein 6 | CHMP6 | 1 | 1 | 23,5 | -0,96 | 0,085 | 0,29 | 0,807 |
| O00299 | Chloride intracellular channel protein 1 | CLIC1 | 4 | 4 | 26,9 | -0,24 | 0,646 | -0,24 | 0,645 |
| O95833 | Chloride intracellular channel protein 3 | CLIC3 | 2 | 2 | 26,6 | -0,43 | 0,705 | -0,18 | 0,947 |
| Q9Y696 | Chloride intracellular channel protein 4 | CLIC4 | 5 | 5 | 28,8 | 0,23 | 0,511 | 0,18 | 0,555 |
| Q9NZA1 | Chloride intracellular channel protein 5 | CLIC5 | 2 | 2 | 46,5 | -0,53 | 0,263 | 0,01 | 0,438 |
| Q96NY7 | Chloride intracellular channel protein 6 | CLIC6 | 11 | 11 | 73,0 | -0,22 | 0,949 | -0,23 | 0,785 |
| Q8IWA5 | Choline transporter-like protein 2 | SLC44A2 | 3 | 3 | 80,1 | -0,73 | 0,211 | 0,06 | 0,556 |
| Q53GD3 | Choline transporter-like protein 4 | SLC44A4 | 3 | 3 | 79,2 | -0,21 | 0,092 | -0,06 | 0,079 |
| Q8IYR0 | Cilia- and flagella-associated protein 206 | CFAP206 | 2 | 2 | 71,1 | 0,02 | 0,435 | -0,20 | 0,862 |
| Q9UL16 | Cilia- and flagella-associated protein 45 | CFAP45 | 1 | 1 | 65,7 | 0,39 | 0,532 | 0,53 | 0,447 |
| Q5T655 | Cilia- and flagella-associated protein 58 | CFAP58 | 1 | 1 | 103,4 | -0,75 | 0,104 | -0,23 | 0,184 |
| Q9P2B7 | Cilia- and flagella-associated protein 97 | CFAP97 | 1 | 1 | 59,4 | -1,18 | 0,009 | 0,24 | 0,782 |
| Q00610 | Clathrin heavy chain 1 | CLTC | 4 | 4 | 191,5 | 0,34 | 0,197 | 0,02 | 0,857 |
| Q9NY35 | Claudin domain-containing protein 1 | CLDND1 | 1 | 1 | 28,6 | -0,11 | 0,948 | 0,02 | 0,979 |
| O95832 | Claudin-1 | CLDN1 | 1 | 1 | 22,7 | 0,16 | 0,534 | -0,77 | 0,189 |
| O15551 | Claudin-3 | CLDN3 | 3 | 3 | 23,3 | 0,07 | 0,706 | -0,54 | 0,024 |
| O14493 | Claudin-4 | CLDN4 | 2 | 2 | 22,1 | -0,20 | 0,906 | -0,42 | 0,136 |
| P10909 | Clusterin | CLU | 7 | 7 | 52,5 | 0,36 | 0,200 | 0,10 | 0,365 |
| Q96AJ1 | Clusterin-associated protein 1 | CLUAP1 | 1 | 1 | 48,1 | 0,28 | 0,209 | -0,11 | 0,894 |
| Q14019 | Coactosin-like protein | COTL1 | 2 | 2 | 15,9 | 0,02 | 0,695 | -0,23 | 0,773 |
| P53621 | Coatomer subunit alpha | COPA | 2 | 2 | 138,3 | 0,94 | 0,124 | 0,77 | 0,310 |
| P53618 | Coatomer subunit beta | COPB1 | 1 | 1 | 107,1 | 0,89 | 0,147 | 0,55 | 0,939 |
| P35606 | Coatomer subunit beta' | COPB2 | 2 | 2 | 102,4 | 0,63 | 0,045 | 0,63 | 0,481 |
| P23528 | Cofilin-1 | CFL1 | 1 | 2 | 18,5 | -0,08 | 0,994 | -0,05 | 0,947 |
| Q9Y281 | Cofilin-2 | CFL2 | 1 | 2 | 18,7 | 0,08 | 0,735 | -0,50 | 0,321 |
| Q6P1N0 | Coiled-coil and C2 domain-containing protein 1A | CC2D1A | 5 | 5 | 104,0 | -0,23 | 0,405 | -0,16 | 0,848 |
| Q9P2K1 | Coiled-coil and C2 domain-containing protein 2A | CC2D2A | 2 | 2 | 186,1 | -0,09 | 0,777 | 0,20 | 0,489 |
| Q96M63 | Coiled-coil domain-containing protein 114 | CCDC114 | 1 | 1 | 75,0 | -0,33 | 0,792 | -0,74 | 0,547 |
| A5D8V7 | Coiled-coil domain-containing protein 151 | CCDC151 | 1 | 1 | 69,1 | -0,70 | 0,720 | 0,29 | 0,854 |
| Q96LX7 | Coiled-coil domain-containing protein 17 | CCDC17 | 2 | 2 | 67,7 | -0,25 | 0,795 | -0,06 | 0,803 |
| Q5TID7 | Coiled-coil domain-containing protein 181 | CCDC181 | 1 | 1 | 60,1 | 0,02 | 0,942 | -0,05 | 0,725 |
| Q16204 | Coiled-coil domain-containing protein 6 | CCDC6 | 1 | 1 | 53,3 | -0,48 | 0,301 | -0,28 | 0,615 |
| A6NI79 | Coiled-coil domain-containing protein 69 | CCDC69 | 1 | 1 | 34,8 | -0,44 | 0,856 | -0,64 | 0,876 |
| A2IDD5 | Coiled-coil domain-containing protein 78 | CCDC78 | 3 | 3 | 48,5 | 0,22 | 0,392 | 0,04 | 0,774 |
| P02452 | Collagen alpha-1(I) chain | COL1A1 | 6 | 6 | 138,9 | 0,21 | 0,992 | 3,10 | 0,207 |
| P39060 | Collagen alpha-1(XVIII) chain | COL18A1 | 1 | 1 | 178,1 | -1,00 | 0,315 | -0,13 | 0,931 |
| P08123 | Collagen alpha-2(I) chain | COL1A2 | 3 | 3 | 129,2 | 0,09 | 0,877 | 4,52 | 0,239 |
| P06681 | Complement C2 | C2 | 1 | 1 | 83,2 | -0,20 | 0,477 | -0,31 | 0,136 |
| P01024 | Complement C3 | C3 | 22 | 22 | 187,0 | 0,05 | 0,579 | -0,60 | 0,674 |
| P0C0L4 | Complement C4-A | C4A | 6 | 6 | 192,7 | -0,05 | 0,599 | -0,17 | 0,978 |
| P08174 | Complement decay-accelerating factor | CD55 | 5 | 5 | 41,4 | -0,28 | 0,190 | 0,96 | 0,084 |
| P00751 | Complement factor B | CFB | 10 | 10 | 85,5 | 1,65 | 0,045 | -0,04 | 0,590 |
| P08603 | Complement factor H | CFH | 1 | 1 | 139,0 | 0,57 | 0,228 | -0,03 | 0,865 |
| Q13098 | COP9 signalosome complex subunit 1 | GPS1 | 1 | 1 | 55,5 | 0,45 | 0,693 | 0,70 | 0,352 |
| Q9UNS2 | COP9 signalosome complex subunit 3 | COPS3 | 1 | 1 | 47,8 | 0,91 | 0,028 | 0,99 | 0,186 |
| Q9BT78 | COP9 signalosome complex subunit 4 | COPS4 | 1 | 1 | 46,2 | 0,43 | 0,288 | -0,05 | 0,861 |
| Q99829 | Copine-1 | CPNE1 | 3 | 3 | 59,0 | 0,12 | 0,142 | 0,09 | 0,864 |
| Q96FN4 | Copine-2 | CPNE2 | 1 | 1 | 61,2 | 0,07 | 0,586 | 0,30 | 0,304 |
| O75131 | Copine-3 | CPNE3 | 3 | 3 | 60,1 | -0,02 | 0,815 | 0,59 | 0,168 |
| Q86YQ8 | Copine-8 | CPNE8 | 2 | 2 | 63,1 | 0,01 | 0,783 | 0,41 | 0,050 |
| O75367 | Core histone macro-H2A.1 | H2AFY | 1 | 1 | 39,6 | 0,16 | 0,644 | 0,95 | 0,042 |
| P35321 | Cornifin-A | SPRR1A | 3 | 3 | 9,9 | 2,07 | 0,663 | 0,70 | 0,402 |
| Q9UBG3 | Cornulin | CRNN | 1 | 1 | 53,5 | 1,04 | 0,278 | 0,92 | 0,293 |
| Q9BR76 | Coronin-1B | CORO1B | 1 | 1 | 54,2 | -0,16 | 0,603 | -0,09 | 0,877 |
| Q92828 | Coronin-2A | CORO2A | 1 | 2 | 59,7 | 0,13 | 0,907 | -0,16 | 0,437 |
| P57737 | Coronin-7 | CORO7 | 1 | 1 | 100,5 | -0,05 | 0,745 | -0,22 | 0,533 |
| P12277 | Creatine kinase B-type | CKB | 3 | 3 | 42,6 | 0,21 | 0,200 | -0,02 | 0,468 |
| P46109 | Crk-like protein | CRKL | 2 | 2 | 33,8 | 0,12 | 0,389 | -0,44 | 0,035 |
| O94886 | CSC1-like protein 1 | TMEM63A | 1 | 1 | 92,1 | 0,15 | 0,697 | -0,10 | 0,704 |
| Q13618 | Cullin-3 | CUL3 | 2 | 2 | 88,9 | -0,15 | 0,645 | 0,04 | 0,668 |
| Q86VP6 | Cullin-associated NEDD8-dissociated protein 1 | CAND1 | 3 | 3 | 136,3 | 0,42 | 0,411 | 0,17 | 0,823 |
| Q00536 | Cyclin-dependent kinase 16 | CDK16 | 1 | 1 | 55,7 | -0,26 | 0,544 | -0,18 | 0,573 |
| Q8ND76 | Cyclin-Y | CCNY | 1 | 1 | 39,3 | 0,41 | 0,011 | 0,51 | 0,186 |
| Q8N7R7 | Cyclin-Y-like protein 1 | CCNYL1 | 1 | 1 | 40,7 | -0,27 | 0,125 | -0,21 | 0,395 |
| P01040 | Cystatin-A | CSTA | 1 | 1 | 11,0 | -0,02 | 0,967 | 0,43 | 0,384 |
| Q9H1C7 | Cysteine-rich and transmembrane domain-containing protein 1 | CYSTM1 | 1 | 1 | 10,6 | 0,43 | 0,098 | -0,04 | 0,921 |
| P50238 | Cysteine-rich protein 1 | CRIP1 | 1 | 1 | 8,5 | 0,49 | 0,691 | 0,15 | 0,689 |
| P52943 | Cysteine-rich protein 2 | CRIP2 | 1 | 1 | 22,5 | -0,39 | 0,468 | -0,21 | 0,442 |
| A8MQ03 | Cysteine-rich tail protein 1 | CYSRT1 | 1 | 1 | 15,3 | -0,10 | 0,969 | 0,07 | 0,658 |
| P13569 | Cystic fibrosis transmembrane conductance regulator | CFTR | 2 | 2 | 168,0 | -0,11 | 0,919 | 0,66 | 0,082 |
| Q53TN4 | Cytochrome b reductase 1 | CYBRD1 | 2 | 2 | 31,6 | -0,45 | 0,265 | -0,59 | 0,285 |
| P49447 | Cytochrome b561 | CYB561 | 1 | 1 | 27,5 | -0,24 | 0,141 | -0,34 | 0,006 |
| Q14204 | Cytoplasmic dynein 1 heavy chain 1 | DYNC1H1 | 11 | 11 | 532,1 | 0,06 | 0,662 | -0,10 | 0,857 |
| Q13409 | Cytoplasmic dynein 1 intermediate chain 2 | DYNC1I2 | 1 | 1 | 71,4 | 0,12 | 0,769 | -0,05 | 0,884 |
| Q8NCM8 | Cytoplasmic dynein 2 heavy chain 1 | DYNC2H1 | 15 | 15 | 492,3 | -0,25 | 0,817 | -0,10 | 0,438 |
| Q7L576 | Cytoplasmic FMR1-interacting protein 1 | CYFIP1 | 8 | 8 | 145,1 | -0,02 | 0,855 | -0,36 | 0,361 |
| P16333 | Cytoplasmic protein NCK1 | NCK1 | 2 | 2 | 42,8 | -0,54 | 0,018 | -0,25 | 0,410 |
| O43639 | Cytoplasmic protein NCK2 | NCK2 | 1 | 1 | 42,9 | -0,58 | 0,148 | -0,50 | 0,403 |
| P28838 | Cytosol aminopeptidase | LAP3 | 5 | 5 | 56,1 | 0,53 | 0,220 | 0,53 | 0,493 |
| Q96KP4 | Cytosolic non-specific dipeptidase | CNDP2 | 6 | 6 | 52,8 | 0,22 | 0,262 | 0,11 | 0,418 |
| Q5M775 | Cytospin-B | SPECC1 | 1 | 1 | 118,5 | 0,30 | 0,625 | -0,86 | 0,284 |
| O43175 | D-3-phosphoglycerate dehydrogenase | PHGDH | 3 | 3 | 56,6 | 0,15 | 0,495 | 0,08 | 0,139 |
| Q9UKG1 | DCC-interacting protein 13-alpha | APPL1 | 2 | 2 | 79,6 | 0,48 | 0,229 | 0,08 | 0,824 |
| Q8NEU8 | DCC-interacting protein 13-beta | APPL2 | 2 | 2 | 74,4 | -0,08 | 0,914 | 0,32 | 0,536 |
| P78560 | Death domain-containing protein CRADD | CRADD | 1 | 1 | 22,7 | -0,04 | 0,813 | -0,35 | 0,071 |
| Q14185 | Dedicator of cytokinesis protein 1 | DOCK1 | 3 | 3 | 215,2 | 0,24 | 0,365 | -0,16 | 0,209 |
| Q9H7D0 | Dedicator of cytokinesis protein 5 | DOCK5 | 3 | 3 | 215,2 | 0,40 | 0,362 | 0,43 | 0,088 |
| Q9BZ29 | Dedicator of cytokinesis protein 9 | DOCK9 | 8 | 8 | 236,3 | -0,19 | 0,405 | -0,12 | 0,040 |
| Q9UGM3 | Deleted in malignant brain tumors 1 protein | DMBT1 | 2 | 2 | 260,6 | -1,02 | 0,346 | -1,12 | 0,604 |
| Q9H6A0 | DENN domain-containing protein 2D | DENND2D | 1 | 1 | 53,6 | 0,09 | 0,815 | 0,17 | 0,548 |
| P49184 | Deoxyribonuclease-1-like 1 | DNASE1L1 | 1 | 1 | 33,9 | -0,40 | 0,111 | -0,56 | 0,192 |
| P81605 | Dermcidin | DCD | 2 | 2 | 11,3 | -1,08 | 0,004 | 0,43 | 0,563 |
| Q08554 | Desmocollin-1 | DSC1 | 3 | 3 | 99,9 | 0,29 | 0,745 | 0,13 | 0,791 |
| Q02413 | Desmoglein-1 | DSG1 | 9 | 9 | 113,7 | 0,82 | 0,793 | 0,49 | 0,664 |
| P15924 | Desmoplakin | DSP | 49 | 49 | 331,6 | 1,68 | 0,575 | 0,85 | 0,455 |
| P60981 | Destrin | DSTN | 2 | 2 | 18,5 | -0,02 | 0,700 | -0,47 | 0,739 |
| Q9NY33 | Dipeptidyl peptidase 3 | DPP3 | 3 | 3 | 82,5 | 0,22 | 0,169 | 0,19 | 0,421 |
| P27487 | Dipeptidyl peptidase 4 | DPP4 | 8 | 8 | 88,2 | -1,00 | 0,295 | 2,71 | 0,039 |
| Q9NZJ9 | Diphosphoinositol polyphosphate phosphohydrolase 2 | NUDT4 | 2 | 2 | 20,3 | -0,78 | 0,080 | -0,51 | 0,420 |
| Q9P265 | Disco-interacting protein 2 homolog B | DIP2B | 8 | 8 | 171,4 | 0,07 | 0,618 | 0,10 | 0,570 |
| Q9Y4D1 | Disheveled-associated activator of morphogenesis 1 | DAAM1 | 3 | 3 | 123,4 | -0,01 | 0,962 | 0,25 | 0,517 |
| O14672 | Disintegrin and metalloproteinase domain-containing protein 10 | ADAM10 | 2 | 2 | 84,1 | 0,34 | 0,228 | -0,37 | 0,313 |
| Q13444 | Disintegrin and metalloproteinase domain-containing protein 15 | ADAM15 | 1 | 1 | 92,9 | -0,55 | 0,388 | -0,72 | 0,216 |
| Q13443 | Disintegrin and metalloproteinase domain-containing protein 9 | ADAM9 | 5 | 5 | 90,5 | -0,10 | 0,547 | 0,20 | 0,466 |
| Q9Y2H0 | Disks large-associated protein 4 | DLGAP4 | 2 | 2 | 107,9 | 0,08 | 0,596 | 0,06 | 0,795 |
| Q16531 | DNA damage-binding protein 1 | DDB1 | 2 | 2 | 126,9 | -0,09 | 0,346 | 0,10 | 0,580 |
| Q8WW22 | DnaJ homolog subfamily A member 4 | DNAJA4 | 2 | 2 | 44,8 | 0,19 | 0,112 | -0,04 | 0,926 |
| P25685 | DnaJ homolog subfamily B member 1 | DNAJB1 | 2 | 2 | 38,0 | -0,03 | 0,942 | 0,09 | 0,125 |
| P59910 | DnaJ homolog subfamily B member 13 | DNAJB13 | 2 | 2 | 36,1 | -0,15 | 0,919 | -0,04 | 0,835 |
| P25686 | DnaJ homolog subfamily B member 2 | DNAJB2 | 2 | 2 | 35,6 | 0,11 | 0,809 | 0,10 | 0,936 |
| O75190 | DnaJ homolog subfamily B member 6 | DNAJB6 | 1 | 1 | 36,1 | -0,17 | 0,837 | 0,02 | 0,564 |
| Q13217 | DnaJ homolog subfamily C member 3 | DNAJC3 | 1 | 1 | 57,5 | -0,40 | 0,778 | 0,05 | 0,529 |
| Q9H3Z4 | DnaJ homolog subfamily C member 5 | DNAJC5 | 1 | 1 | 22,1 | -0,14 | 0,710 | -0,02 | 0,971 |
| Q99615 | DnaJ homolog subfamily C member 7 | DNAJC7 | 1 | 1 | 56,4 | 1,11 | 0,035 | -0,09 | 0,795 |
| A2VCK2 | Doublecortin domain-containing protein 2B | DCDC2B | 2 | 2 | 37,6 | -0,76 | 0,294 | 0,60 | 0,442 |
| Q9UJU6 | Drebrin-like protein | DBNL | 2 | 2 | 48,2 | -0,15 | 0,984 | -0,49 | 0,157 |
| Q9UN19 | Dual adapter for phosphotyrosine and 3-phosphotyrosine and 3-phosphoinositide | DAPP1 | 5 | 5 | 32,2 | 2,63 | 0,002 | 0,42 | 0,668 |
| Q9NRD9 | Dual oxidase 1 | DUOX1 | 5 | 8 | 177,1 | 0,43 | 0,156 | -0,21 | 0,600 |
| Q9NRD8 | Dual oxidase 2 | DUOX2 | 6 | 9 | 175,3 | 2,86 | 0,000 | -0,24 | 0,475 |
| Q1HG43 | Dual oxidase maturation factor 1 | DUOXA1 | 3 | 3 | 37,8 | -0,22 | 0,839 | -0,15 | 0,652 |
| Q1HG44 | Dual oxidase maturation factor 2 | DUOXA2 | 1 | 1 | 34,8 | 3,51 | 0,008 | -0,01 | 0,869 |
| P36507 | Dual specificity mitogen-activated protein kinase kinase 2 | MAP2K2 | 1 | 1 | 44,4 | -0,19 | 0,736 | -0,22 | 0,733 |
| P45985 | Dual specificity mitogen-activated protein kinase kinase 4 | MAP2K4 | 1 | 1 | 44,3 | 0,21 | 0,022 | 0,18 | 0,466 |
| P52564 | Dual specificity mitogen-activated protein kinase kinase 6 | MAP2K6 | 2 | 2 | 37,5 | 0,01 | 0,851 | -0,25 | 0,640 |
| Q14203 | Dynactin subunit 1 | DCTN1 | 1 | 1 | 141,6 | 0,60 | 0,398 | -0,22 | 0,963 |
| P50570 | Dynamin-2 | DNM2 | 1 | 1 | 98,0 | 0,26 | 0,427 | 0,06 | 0,688 |
| Q96DT5 | Dynein heavy chain 11, axonemal | DNAH11 | 1 | 2 | 520,0 | 0,26 | 0,433 | 0,34 | 0,070 |
| Q8TE73 | Dynein heavy chain 5, axonemal | DNAH5 | 10 | 10 | 528,7 | -0,49 | 0,839 | -0,06 | 0,993 |
| Q9NYC9 | Dynein heavy chain 9, axonemal | DNAH9 | 4 | 5 | 511,6 | -0,39 | 0,695 | -0,31 | 0,125 |
| O96015 | Dynein light chain 4, axonemal | DNAL4 | 1 | 1 | 12,0 | -0,03 | 0,720 | -0,82 | 0,324 |
| Q14258 | E3 ubiquitin/ISG15 ligase TRIM25 | TRIM25 | 2 | 2 | 70,9 | -0,27 | 0,048 | -0,08 | 0,692 |
| Q9UNE7 | E3 ubiquitin-protein ligase CHIP | STUB1 | 2 | 2 | 34,8 | 0,00 | 0,997 | 0,19 | 0,547 |
| Q7Z6Z7 | E3 ubiquitin-protein ligase HUWE1 | HUWE1 | 1 | 1 | 481,6 | 0,50 | 0,127 | -0,39 | 0,460 |
| Q96J02 | E3 ubiquitin-protein ligase Itchy homolog | ITCH | 1 | 1 | 102,7 | -0,09 | 0,634 | -0,01 | 0,984 |
| Q9Y508 | E3 ubiquitin-protein ligase RNF114 | RNF114 | 1 | 1 | 25,7 | 0,03 | 0,921 | -0,42 | 0,200 |
| Q7Z6J0 | E3 ubiquitin-protein ligase SH3RF1 | SH3RF1 | 1 | 1 | 93,1 | -0,61 | 0,215 | 0,11 | 0,582 |
| Q9HCE7 | E3 ubiquitin-protein ligase SMURF1 | SMURF1 | 2 | 2 | 86,1 | -0,21 | 0,760 | 0,05 | 0,599 |
| Q9BZY9 | E3 ubiquitin-protein ligase TRIM31 | TRIM31 | 1 | 1 | 48,2 | 1,85 | 0,138 | 0,07 | 0,674 |
| O95834 | Echinoderm microtubule-associated protein-like 2 | EML2 | 1 | 1 | 70,6 | 0,01 | 0,702 | 0,12 | 0,681 |
| O75355 | Ectonucleoside triphosphate diphosphohydrolase 3 | ENTPD3 | 1 | 1 | 59,1 | -0,49 | 0,382 | 0,28 | 0,256 |
| A8K855 | EF-hand calcium-binding domain-containing protein 7 | EFCAB7 | 1 | 1 | 71,9 | -0,28 | 0,578 | -0,27 | 0,460 |
| Q5JST6 | EF-hand domain-containing family member C2 | EFHC2 | 1 | 1 | 87,3 | -0,47 | 0,977 | 0,13 | 0,605 |
| Q5JVL4 | EF-hand domain-containing protein 1 | EFHC1 | 1 | 1 | 73,9 | 0,05 | 0,460 | 0,13 | 0,205 |
| Q96C19 | EF-hand domain-containing protein D2 | EFHD2 | 2 | 2 | 26,7 | 0,13 | 0,647 | -0,25 | 0,692 |
| O43854 | EGF-like repeat and discoidin I-like domain-containing protein 3 | EDIL3 | 1 | 1 | 53,7 | -0,43 | 0,837 | -0,39 | 0,800 |
| Q9H4M9 | EH domain-containing protein 1 | EHD1 | 6 | 6 | 60,6 | 0,23 | 0,299 | -0,51 | 0,019 |
| Q9NZN4 | EH domain-containing protein 2 | EHD2 | 3 | 3 | 61,1 | -0,49 | 0,142 | -0,10 | 0,935 |
| Q9H223 | EH domain-containing protein 4 | EHD4 | 9 | 9 | 61,1 | -0,02 | 0,876 | -0,42 | 0,059 |
| P68104 | Elongation factor 1-alpha 1 | EEF1A1 | 3 | 3 | 50,1 | 0,16 | 0,197 | 0,21 | 0,078 |
| P26641 | Elongation factor 1-gamma | EEF1G | 1 | 1 | 50,1 | 1,27 | 0,012 | 0,33 | 0,708 |
| P13639 | Elongation factor 2 | EEF2 | 6 | 6 | 95,3 | 0,10 | 0,462 | 0,22 | 0,071 |
| Q99961 | Endophilin-A2 | SH3GL1 | 3 | 3 | 41,5 | 0,33 | 0,031 | 0,12 | 0,405 |
| Q9Y371 | Endophilin-B1 | SH3GLB1 | 2 | 2 | 40,8 | -0,33 | 0,009 | -0,38 | 0,288 |
| P14625 | Endoplasmin | HSP90B1 | 2 | 3 | 92,4 | 0,74 | 0,097 | 0,22 | 0,442 |
| Q96JJ3 | Engulfment and cell motility protein 2 | ELMO2 | 3 | 3 | 82,6 | 0,43 | 0,075 | -0,08 | 0,364 |
| Q92817 | Envoplakin | EVPL | 27 | 27 | 231,5 | 0,06 | 0,697 | -0,08 | 0,552 |
| Q12929 | Epidermal growth factor receptor kinase substrate 8 | EPS8 | 13 | 13 | 91,8 | -0,33 | 0,478 | -0,54 | 0,087 |
| Q8TE68 | Epidermal growth factor receptor kinase substrate 8-like protein 1 | EPS8L1 | 14 | 14 | 80,2 | -0,76 | 0,065 | -0,21 | 0,108 |
| Q9H6S3 | Epidermal growth factor receptor kinase substrate 8-like protein 2 | EPS8L2 | 10 | 10 | 80,6 | -0,59 | 0,254 | -0,19 | 0,044 |
| Q8TE67 | Epidermal growth factor receptor kinase substrate 8-like protein 3 | EPS8L3 | 2 | 2 | 66,8 | 0,75 | 0,028 | 0,61 | 0,292 |
| P58107 | Epiplakin | EPPK1 | 2 | 3 | 555,3 | 0,72 | 0,039 | 0,76 | 0,111 |
| Q9Y6I3 | Epsin-1 | EPN1 | 2 | 2 | 60,3 | -0,36 | 0,058 | -0,27 | 0,540 |
| P27105 | Erythrocyte band 7 integral membrane protein | STOM | 5 | 5 | 31,7 | 0,14 | 0,510 | 0,35 | 0,289 |
| Q99447 | Ethanolamine-phosphate cytidylyltransferase | PCYT2 | 2 | 2 | 43,8 | -0,18 | 0,746 | -0,60 | 0,182 |
| P60842 | Eukaryotic initiation factor 4A-I | EIF4A1 | 2 | 2 | 46,1 | 0,41 | 0,417 | 0,24 | 0,486 |
| Q9Y262 | Eukaryotic translation initiation factor 3 subunit L | EIF3L | 1 | 1 | 66,7 | 0,15 | 0,701 | 0,38 | 0,207 |
| P78344 | Eukaryotic translation initiation factor 4 gamma 2 | EIF4G2 | 1 | 1 | 102,3 | 0,95 | 0,053 | -0,17 | 0,818 |
| P23588 | Eukaryotic translation initiation factor 4B | EIF4B | 2 | 2 | 69,1 | -0,22 | 0,504 | 0,47 | 0,243 |
| O60573 | Eukaryotic translation initiation factor 4E type 2 | EIF4E2 | 1 | 1 | 28,3 | -0,43 | 0,372 | -0,10 | 0,876 |
| Q15056 | Eukaryotic translation initiation factor 4H | EIF4H | 1 | 1 | 27,4 | -0,25 | 0,456 | -0,63 | 0,124 |
| Q9GZV4 | Eukaryotic translation initiation factor 5A-2 | EIF5A2 | 1 | 1 | 16,8 | -0,05 | 0,848 | -0,18 | 0,420 |
| P56537 | Eukaryotic translation initiation factor 6 | EIF6 | 1 | 1 | 26,6 | 0,36 | 0,388 | 0,39 | 0,598 |
| P43005 | Excitatory amino acid transporter 3 | SLC1A1 | 1 | 1 | 57,1 | -1,00 | 0,033 | -1,65 | 0,006 |
| Q9NV70 | Exocyst complex component 1 | EXOC1 | 3 | 3 | 101,9 | 0,08 | 0,487 | 0,03 | 0,941 |
| Q96A65 | Exocyst complex component 4 | EXOC4 | 3 | 3 | 110,4 | -0,45 | 0,505 | -0,25 | 0,603 |
| O00471 | Exocyst complex component 5 | EXOC5 | 1 | 1 | 81,8 | -0,49 | 0,361 | -0,29 | 0,310 |
| Q9UPT5 | Exocyst complex component 7 | EXOC7 | 2 | 2 | 83,3 | -0,15 | 0,210 | -0,25 | 0,438 |
| Q8IYI6 | Exocyst complex component 8 | EXOC8 | 1 | 1 | 81,7 | 0,01 | 0,930 | 0,19 | 0,111 |
| P15311 | Ezrin | EZR | 13 | 17 | 69,4 | -0,73 | 0,071 | -0,34 | 0,330 |
| P52907 | F-actin-capping protein subunit alpha-1 | CAPZA1 | 3 | 3 | 32,9 | 0,27 | 0,376 | -0,08 | 0,970 |
| P47755 | F-actin-capping protein subunit alpha-2 | CAPZA2 | 2 | 2 | 32,9 | 0,19 | 0,478 | -0,09 | 0,796 |
| P47756 | F-actin-capping protein subunit beta | CAPZB | 3 | 3 | 31,3 | 0,30 | 0,058 | -0,08 | 0,776 |
| Q5VZK9 | F-actin-uncapping protein LRRC16A | CARMIL1 | 1 | 1 | 151,5 | -0,34 | 0,901 | -0,19 | 0,970 |
| P14324 | Farnesyl pyrophosphate synthase | FDPS | 1 | 1 | 48,2 | 0,67 | 0,211 | 0,34 | 0,493 |
| Q9NVQ4 | Fas apoptotic inhibitory molecule 1 | FAIM | 1 | 1 | 20,2 | -0,32 | 0,659 | -0,53 | 0,295 |
| P49327 | Fatty acid synthase | FASN | 10 | 10 | 273,3 | 0,20 | 0,430 | 0,70 | 0,176 |
| Q01469 | Fatty acid-binding protein, epidermal | FABP5 | 2 | 2 | 15,2 | 0,46 | 0,864 | -0,22 | 0,370 |
| Q9UK22 | F-box only protein 2 | FBXO2 | 1 | 1 | 33,3 | -0,95 | 0,434 | -0,77 | 0,127 |
| Q5XUX1 | F-box/WD repeat-containing protein 9 | FBXW9 | 1 | 1 | 54,1 | -0,89 | 0,165 | 0,35 | 0,749 |
| Q2WGJ9 | Fer-1-like protein 6 | FER1L6 | 12 | 12 | 209,2 | -0,66 | 0,067 | 0,07 | 0,524 |
| P02751 | Fibronectin | FN1 | 13 | 13 | 262,5 | -0,83 | 0,035 | 0,05 | 0,450 |
| Q8TC84 | Fibronectin type 3 and ankyrin repeat domains protein 1 | FANK1 | 1 | 1 | 38,3 | -0,18 | 0,037 | -0,34 | 0,897 |
| P98095 | Fibulin-2 | FBLN2 | 1 | 1 | 126,5 | -1,65 | 0,138 | -0,17 | 0,235 |
| P20930 | Filaggrin | FLG | 3 | 3 | 434,9 | -0,07 | 0,439 | -0,28 | 0,407 |
| Q5D862 | Filaggrin-2 | FLG2 | 5 | 5 | 247,9 | 0,08 | 0,892 | 0,54 | 0,684 |
| P21333 | Filamin-A | FLNA | 2 | 4 | 280,6 | 0,38 | 0,675 | 0,33 | 0,792 |
| O75369 | Filamin-B | FLNB | 20 | 22 | 278,0 | 0,33 | 0,329 | 0,20 | 0,113 |
| P30043 | Flavin reductase (NADPH) | BLVRB | 2 | 2 | 22,1 | 0,23 | 0,507 | 0,03 | 0,906 |
| O75955 | Flotillin-1 | FLOT1 | 8 | 8 | 47,3 | -0,26 | 0,256 | -0,38 | 0,130 |
| Q14254 | Flotillin-2 | FLOT2 | 7 | 7 | 47,0 | -0,41 | 0,188 | -0,56 | 0,003 |
| P15328 | Folate receptor alpha | FOLR1 | 2 | 2 | 29,8 | -0,63 | 0,112 | 0,00 | 0,587 |
| Q5T0N5 | Formin-binding protein 1-like | FNBP1L | 2 | 2 | 70,0 | -0,40 | 0,074 | -0,52 | 0,020 |
| Q9H479 | Fructosamine-3-kinase | FN3K | 1 | 1 | 35,1 | -0,65 | 0,116 | 0,08 | 0,688 |
| P04075 | Fructose-bisphosphate aldolase A | ALDOA | 5 | 6 | 39,4 | 0,20 | 0,026 | 0,42 | 0,078 |
| P09972 | Fructose-bisphosphate aldolase C | ALDOC | 1 | 2 | 39,4 | 0,33 | 0,007 | 0,50 | 0,185 |
| Q96M96 | FYVE, RhoGEF and PH domain-containing protein 4 | FGD4 | 1 | 1 | 86,6 | 1,01 | 0,007 | 0,11 | 0,914 |
| P43250 | G protein-coupled receptor kinase 6 | GRK6 | 2 | 2 | 65,9 | -0,11 | 0,762 | 0,32 | 0,100 |
| P07902 | Galactose-1-phosphate uridylyltransferase | GALT | 1 | 1 | 43,3 | 1,85 | 0,322 | 0,55 | 0,109 |
| P21217 | Galactoside 3(4)-L-fucosyltransferase | FUT3 | 1 | 1 | 42,1 | -1,19 | 0,268 | -1,00 | 0,484 |
| P17931 | Galectin-3 | LGALS3 | 3 | 3 | 26,1 | -0,03 | 0,820 | 0,20 | 0,251 |
| Q08380 | Galectin-3-binding protein | LGALS3BP | 1 | 1 | 65,3 | 0,03 | 0,881 | 0,49 | 0,272 |
| P47929 | Galectin-7 | LGALS7 | 1 | 1 | 15,1 | 0,68 | 0,240 | 0,91 | 0,158 |
| O00182 | Galectin-9 | LGALS9 | 2 | 2 | 39,5 | -0,81 | 0,016 | 0,16 | 0,807 |
| Q92820 | Gamma-glutamyl hydrolase | GGH | 1 | 1 | 35,9 | 0,20 | 0,320 | 0,06 | 0,983 |
| O75223 | Gamma-glutamylcyclotransferase | GGCT | 3 | 3 | 21,0 | -0,17 | 0,481 | 0,13 | 0,647 |
| Q99747 | Gamma-soluble NSF attachment protein | NAPG | 5 | 5 | 34,7 | -0,24 | 0,063 | -0,25 | 0,094 |
| Q96QA5 | Gasdermin-A | GSDMA | 2 | 2 | 49,3 | 0,54 | 0,937 | 0,16 | 0,847 |
| Q13630 | GDP-L-fucose synthase | TSTA3 | 1 | 1 | 35,9 | 0,28 | 0,141 | 0,50 | 0,185 |
| O60547 | GDP-mannose 4,6 dehydratase | GMDS | 2 | 2 | 41,9 | 0,21 | 0,370 | 0,36 | 0,723 |
| P06396 | Gelsolin | GSN | 12 | 12 | 85,6 | 0,13 | 0,127 | 1,15 | 0,022 |
| Q92696 | Geranylgeranyl transferase type-2 subunit alpha | RABGGTA | 1 | 1 | 65,0 | 0,89 | 0,144 | 0,98 | 0,223 |
| P60983 | Glia maturation factor beta | GMFB | 2 | 2 | 16,7 | 0,16 | 0,453 | 0,26 | 0,160 |
| Q6PCE3 | Glucose 1,6-bisphosphate synthase | PGM2L1 | 2 | 2 | 70,4 | 0,51 | 0,028 | 0,36 | 0,256 |
| P11413 | Glucose-6-phosphate 1-dehydrogenase | G6PD | 2 | 2 | 59,2 | 0,01 | 0,982 | 0,13 | 0,013 |
| P06744 | Glucose-6-phosphate isomerase | GPI | 3 | 3 | 63,1 | -0,14 | 0,370 | 0,05 | 0,814 |
| P48506 | Glutamate--cysteine ligase catalytic subunit | GCLC | 2 | 2 | 72,7 | -0,20 | 0,152 | 0,01 | 0,987 |
| Q5RHP9 | Glutamate-rich protein 3 | ERICH3 | 5 | 5 | 168,4 | -0,57 | 0,381 | -0,03 | 0,350 |
| Q6P6B1 | Glutamate-rich protein 5 | ERICH5 | 3 | 3 | 39,9 | -0,97 | 0,211 | -1,50 | 0,019 |
| P15104 | Glutamine synthetase | GLUL | 1 | 1 | 42,0 | 0,33 | 0,055 | -0,01 | 0,715 |
| P07203 | Glutathione peroxidase 1 | GPX1 | 3 | 3 | 22,1 | 0,02 | 0,686 | -0,27 | 0,649 |
| P00390 | Glutathione reductase, mitochondrial | GSR | 2 | 2 | 56,2 | 0,41 | 0,285 | 0,25 | 0,232 |
| P08263 | Glutathione S-transferase A1 | GSTA1 | 2 | 2 | 25,6 | -0,14 | 0,648 | -0,19 | 0,515 |
| P78417 | Glutathione S-transferase omega-1 | GSTO1 | 1 | 1 | 27,5 | 0,51 | 0,537 | 0,13 | 0,375 |
| P09211 | Glutathione S-transferase P | GSTP1 | 2 | 2 | 23,3 | -0,25 | 0,760 | 0,41 | 0,402 |
| P48637 | Glutathione synthetase | GSS | 3 | 3 | 52,4 | 0,07 | 0,741 | 0,30 | 0,251 |
| P04406 | Glyceraldehyde-3-phosphate dehydrogenase | GAPDH | 3 | 3 | 36,0 | 0,06 | 0,702 | 0,41 | 0,023 |
| Q14409 | Glycerol kinase 3 | GK3P | 1 | 1 | 60,6 | 0,27 | 0,437 | 0,25 | 0,767 |
| Q8N335 | Glycerol-3-phosphate dehydrogenase 1-like protein | GPD1L | 4 | 4 | 38,4 | 0,38 | 0,233 | 0,14 | 0,751 |
| P41250 | Glycine--tRNA ligase | GARS | 1 | 1 | 83,1 | 0,30 | 0,586 | 0,23 | 0,593 |
| P11216 | Glycogen phosphorylase, brain form | PYGB | 3 | 3 | 96,6 | 0,33 | 0,197 | 0,61 | 0,015 |
| P49840 | Glycogen synthase kinase-3 alpha | GSK3A | 1 | 1 | 50,9 | -0,34 | 0,038 | -0,11 | 0,575 |
| Q9NZD2 | Glycolipid transfer protein | GLTP | 2 | 2 | 23,8 | 0,03 | 0,421 | -0,04 | 0,826 |
| Q9HC38 | Glyoxalase domain-containing protein 4 | GLOD4 | 1 | 1 | 34,8 | -0,01 | 0,957 | -0,03 | 0,847 |
| Q9P2T1 | GMP reductase 2 | GMPR2 | 1 | 1 | 37,9 | 0,48 | 0,122 | 0,21 | 0,951 |
| Q9H4A6 | Golgi phosphoprotein 3 | GOLPH3 | 1 | 1 | 33,8 | 0,27 | 0,591 | -0,17 | 0,953 |
| Q9H4G4 | Golgi-associated plant pathogenesis-related protein 1 | GLIPR2 | 3 | 3 | 17,2 | -0,22 | 0,052 | -0,15 | 0,413 |
| Q7Z5G4 | Golgin subfamily A member 7 | GOLGA7 | 2 | 2 | 15,8 | -0,15 | 0,057 | 0,16 | 0,767 |
| Q9NZH0 | G-protein coupled receptor family C group 5 member B | GPRC5B | 1 | 1 | 44,8 | -0,74 | 0,355 | -0,71 | 0,618 |
| Q9NQ84 | G-protein coupled receptor family C group 5 member C | GPRC5C | 6 | 6 | 48,2 | -0,67 | 0,102 | -0,61 | 0,082 |
| P09919 | Granulocyte colony-stimulating factor | CSF3 | 1 | 1 | 22,3 | 5,04 | 0,021 | -0,24 | 0,624 |
| P62993 | Growth factor receptor-bound protein 2 | GRB2 | 3 | 3 | 25,2 | 0,40 | 0,236 | 0,20 | 0,255 |
| Q5TC63 | Growth hormone-regulated TBC protein 1 | GRTP1 | 1 | 1 | 38,5 | 0,07 | 0,757 | 0,30 | 0,677 |
| Q99988 | Growth/differentiation factor 15 | GDF15 | 1 | 1 | 34,1 | 0,27 | 0,558 | -0,41 | 0,444 |
| P01112 | GTPase Hras | HRAS | 1 | 2 | 21,3 | -0,07 | 0,678 | -0,21 | 0,493 |
| P01116 | GTPase Kras | KRAS | 1 | 2 | 21,6 | -0,34 | 0,122 | 0,15 | 0,663 |
| P55042 | GTP-binding protein RAD | RRAD | 1 | 1 | 33,2 | -0,14 | 0,820 | -0,20 | 0,107 |
| Q15382 | GTP-binding protein Rheb | RHEB | 3 | 3 | 20,5 | 0,03 | 0,586 | -0,07 | 0,732 |
| Q92963 | GTP-binding protein Rit1 | RIT1 | 1 | 1 | 25,1 | -0,03 | 0,283 | -0,44 | 0,070 |
| Q9Y2T3 | Guanine deaminase | GDA | 1 | 1 | 51,0 | 0,42 | 0,095 | -0,15 | 0,467 |
| P63096 | Guanine nucleotide-binding protein G(i) subunit alpha-1 | GNAI1 | 3 | 4 | 40,3 | -0,13 | 0,066 | 0,21 | 0,400 |
| P04899 | Guanine nucleotide-binding protein G(i) subunit alpha-2 | GNAI2 | 4 | 5 | 40,4 | -0,34 | 0,043 | -0,09 | 0,578 |
| Q9UBI6 | Guanine nucleotide-binding protein G(I)/G(S)/G(O) subunit gamma-12 | GNG12 | 1 | 1 | 8,0 | 0,21 | 0,558 | 0,14 | 0,997 |
| P63218 | Guanine nucleotide-binding protein G(I)/G(S)/G(O) subunit gamma-5 | GNG5 | 2 | 2 | 7,3 | -0,07 | 0,374 | 0,34 | 0,460 |
| P62873 | Guanine nucleotide-binding protein G(I)/G(S)/G(T) subunit beta-1 | GNB1 | 2 | 4 | 37,4 | -0,26 | 0,163 | 0,04 | 0,764 |
| P08754 | Guanine nucleotide-binding protein G(k) subunit alpha | GNAI3 | 3 | 4 | 40,5 | 0,08 | 0,663 | 0,09 | 0,985 |
| P50148 | Guanine nucleotide-binding protein G(q) subunit alpha | GNAQ | 5 | 7 | 42,1 | -0,23 | 0,258 | -0,04 | 0,849 |
| P63092 | Guanine nucleotide-binding protein G(s) subunit alpha isoforms short | GNAS | 5 | 5 | 45,6 | -0,28 | 0,388 | -0,24 | 0,455 |
| P29992 | Guanine nucleotide-binding protein subunit alpha-11 | GNA11 | 2 | 5 | 42,1 | -0,09 | 0,826 | -0,12 | 0,407 |
| Q14344 | Guanine nucleotide-binding protein subunit alpha-13 | GNA13 | 4 | 6 | 44,0 | -0,23 | 0,055 | -0,17 | 0,247 |
| O95837 | Guanine nucleotide-binding protein subunit alpha-14 | GNA14 | 1 | 3 | 41,5 | -0,24 | 0,795 | -0,72 | 0,137 |
| P30679 | Guanine nucleotide-binding protein subunit alpha-15 | GNA15 | 2 | 3 | 43,5 | 0,17 | 0,420 | -0,20 | 0,585 |
| Q9HAV0 | Guanine nucleotide-binding protein subunit beta-4 | GNB4 | 1 | 4 | 37,5 | -0,19 | 0,816 | -0,09 | 0,953 |
| P32455 | Guanylate-binding protein 1 | GBP1 | 2 | 2 | 67,9 | 0,31 | 0,404 | 0,14 | 0,598 |
| Q6ZN66 | Guanylate-binding protein 6 | GBP6 | 2 | 2 | 72,4 | -0,11 | 0,907 | 0,01 | 0,865 |
| Q9Y450 | HBS1-like protein | HBS1L | 1 | 1 | 75,4 | 0,26 | 0,317 | 0,70 | 0,247 |
| P0DMV8 | Heat shock 70 kDa protein 1A | HSPA1A | 6 | 15 | 70,0 | 0,07 | 0,642 | 0,10 | 0,432 |
| P34931 | Heat shock 70 kDa protein 1-like | HSPA1L | 1 | 9 | 70,3 | 0,85 | 0,011 | 0,30 | 0,733 |
| P34932 | Heat shock 70 kDa protein 4 | HSPA4 | 3 | 3 | 94,3 | 0,30 | 0,018 | 0,46 | 0,220 |
| P11142 | Heat shock cognate 71 kDa protein | HSPA8 | 11 | 15 | 70,9 | 0,06 | 0,587 | 0,07 | 0,549 |
| Q92598 | Heat shock protein 105 kDa | HSPH1 | 2 | 2 | 96,8 | 0,43 | 0,169 | 0,30 | 0,508 |
| P04792 | Heat shock protein beta-1 | HSPB1 | 5 | 5 | 22,8 | 0,25 | 0,404 | 0,11 | 0,431 |
| P07900 | Heat shock protein HSP 90-alpha | HSP90AA1 | 4 | 9 | 84,6 | 0,67 | 0,001 | 0,40 | 0,066 |
| P08238 | Heat shock protein HSP 90-beta | HSP90AB1 | 4 | 10 | 83,2 | 0,31 | 0,251 | 0,36 | 0,226 |
| Q9Y5Z4 | Heme-binding protein 2 | HEBP2 | 1 | 1 | 22,9 | -0,24 | 0,440 | -0,44 | 0,033 |
| P68871 | Hemoglobin subunit beta | HBB | 1 | 1 | 16,0 | -1,17 | 0,005 | 0,35 | 0,695 |
| O14964 | Hepatocyte growth factor-regulated tyrosine kinase substrate | HGS | 3 | 3 | 86,1 | 0,00 | 0,849 | -0,17 | 0,439 |
| Q5SSJ5 | Heterochromatin protein 1-binding protein 3 | HP1BP3 | 1 | 1 | 61,2 | -0,47 | 0,877 | 0,02 | 0,616 |
| P61978 | Heterogeneous nuclear ribonucleoprotein K | HNRNPK | 2 | 2 | 50,9 | 0,34 | 0,481 | 0,26 | 0,244 |
| P14866 | Heterogeneous nuclear ribonucleoprotein L | HNRNPL | 1 | 1 | 64,1 | 0,59 | 0,329 | 0,79 | 0,128 |
| Q00839 | Heterogeneous nuclear ribonucleoprotein U | HNRNPU | 1 | 1 | 90,5 | 0,17 | 0,468 | 0,42 | 0,150 |
| P22626 | Heterogeneous nuclear ribonucleoproteins A2/B1 | HNRNPA2B1 | 1 | 1 | 37,4 | 0,67 | 0,170 | 1,18 | 0,018 |
| P50135 | Histamine N-methyltransferase | HNMT | 2 | 2 | 33,3 | -0,03 | 0,587 | -0,05 | 0,784 |
| P49773 | Histidine triad nucleotide-binding protein 1 | HINT1 | 1 | 1 | 13,8 | 0,07 | 0,600 | 0,16 | 0,860 |
| Q9NQE9 | Histidine triad nucleotide-binding protein 3 | HINT3 | 1 | 1 | 20,3 | -0,31 | 0,683 | -0,42 | 0,243 |
| P12081 | Histidine--tRNA ligase, cytoplasmic | HARS | 3 | 3 | 57,4 | -0,11 | 0,342 | -0,21 | 0,436 |
| P07305 | Histone H1.0 | H1F0 | 3 | 3 | 20,9 | -0,91 | 0,766 | 0,08 | 0,356 |
| P16403 | Histone H1.2 | HIST1H1C | 1 | 5 | 21,4 | -0,82 | 0,714 | 0,35 | 0,562 |
| P10412 | Histone H1.4 | HIST1H1E | 1 | 5 | 21,9 | -0,33 | 0,930 | 0,91 | 0,099 |
| P16401 | Histone H1.5 | HIST1H1B | 3 | 5 | 22,6 | -0,32 | 0,901 | 1,01 | 0,077 |
| Q96KK5 | Histone H2A type 1-H | HIST1H2AH | 1 | 1 | 13,9 | -0,19 | 0,923 | 0,43 | 0,211 |
| P06899 | Histone H2B type 1-J | HIST1H2BJ | 1 | 4 | 13,9 | -0,27 | 0,560 | -0,16 | 0,464 |
| O60814 | Histone H2B type 1-K | HIST1H2BK | 1 | 4 | 13,9 | -0,55 | 0,730 | -0,02 | 0,994 |
| P68431 | Histone H3.1 | HIST1H3A | 1 | 3 | 15,4 | -0,95 | 0,618 | 0,63 | 0,216 |
| P84243 | Histone H3.3 | H3F3A | 1 | 3 | 15,3 | -1,01 | 0,925 | 0,26 | 0,292 |
| P62805 | Histone H4 | HIST1H4A | 2 | 2 | 11,4 | 0,15 | 0,648 | 0,57 | 0,076 |
| P30461 | HLA class I histocompatibility antigen, B-13 alpha chain | HLA-B | 1 | 1 | 40,4 | 0,40 | 0,503 | -0,51 | 0,902 |
| P01903 | HLA class II histocompatibility antigen, DR alpha chain | HLA-DRA | 1 | 1 | 28,6 | 0,58 | 0,112 | -0,25 | 0,847 |
| Q30154 | HLA class II histocompatibility antigen, DR beta 5 chain | HLA-DRB5 | 2 | 3 | 30,0 | 0,19 | 0,394 | -0,02 | 0,854 |
| Q9TQE0 | HLA class II histocompatibility antigen, DRB1-9 beta chain | HLA-DRB1 | 2 | 3 | 29,8 | 0,62 | 0,101 | -0,25 | 0,809 |
| Q9NSC5 | Homer protein homolog 3 | HOMER3 | 1 | 1 | 39,8 | -0,45 | 0,227 | -0,28 | 0,458 |
| Q86YZ3 | Hornerin | HRNR | 7 | 7 | 282,2 | 0,31 | 0,938 | 0,31 | 0,757 |
| Q16543 | Hsp90 co-chaperone Cdc37 | CDC37 | 1 | 1 | 44,4 | 0,04 | 0,792 | 0,63 | 0,169 |
| Q16775 | Hydroxyacylglutathione hydrolase, mitochondrial | HAGH | 1 | 1 | 33,8 | -0,17 | 0,568 | -0,22 | 0,163 |
| Q8TDS4 | Hydroxycarboxylic acid receptor 2 | HCAR2 | 2 | 2 | 41,8 | -0,51 | 0,272 | -0,06 | 0,811 |
| P01857 | Ig gamma-1 chain C region | IGHG1 | 1 | 1 | 36,1 | 1,48 | 0,758 | 0,71 | 0,463 |
| P01859 | Ig gamma-2 chain C region | IGHG2 | 1 | 1 | 35,9 | 0,70 | 0,810 | 0,50 | 0,570 |
| P0CG05 | Ig lambda-2 chain C regions | IGLC2 | 3 | 3 | 11,3 | 0,19 | 0,900 | 0,67 | 0,409 |
| Q9Y6R7 | IgGFc-binding protein | FCGBP | 18 | 18 | 571,6 | 1,59 | 0,126 | 0,67 | 0,151 |
| Q9GZP8 | Immortalization up-regulated protein | IMUP | 1 | 1 | 10,9 | -0,44 | 0,071 | 0,36 | 0,547 |
| P01877 | Immunoglobulin heavy constant alpha 2 | IGHA2 | 2 | 2 | 36,6 | -0,54 | 0,201 | 0,38 | 0,624 |
| P01591 | Immunoglobulin J chain | JCHAIN | 1 | 1 | 18,1 | -0,88 | 0,048 | 1,18 | 0,298 |
| Q14974 | Importin subunit beta-1 | KPNB1 | 1 | 1 | 97,1 | 0,76 | 0,032 | 0,61 | 0,345 |
| O00410 | Importin-5 | IPO5 | 1 | 1 | 123,5 | 0,51 | 0,253 | 0,39 | 0,596 |
| Q8NI35 | InaD-like protein | PATJ | 3 | 3 | 196,2 | -0,12 | 0,635 | -0,25 | 0,265 |
| P29218 | Inositol monophosphatase 1 | IMPA1 | 1 | 1 | 30,2 | 0,20 | 0,447 | -0,07 | 0,383 |
| Q9NPH2 | Inositol-3-phosphate synthase 1 | ISYNA1 | 1 | 1 | 61,0 | -0,19 | 0,264 | 0,16 | 0,703 |
| P17301 | Integrin alpha-2 | ITGA2 | 3 | 3 | 129,2 | 0,45 | 0,061 | 0,17 | 0,060 |
| P26006 | Integrin alpha-3 | ITGA3 | 4 | 4 | 116,5 | 0,00 | 0,700 | -0,44 | 0,131 |
| P06756 | Integrin alpha-V | ITGAV | 2 | 2 | 116,0 | -0,30 | 0,880 | -0,23 | 0,979 |
| P05556 | Integrin beta-1 | ITGB1 | 3 | 3 | 88,4 | -0,73 | 0,801 | -1,43 | 0,405 |
| O14713 | Integrin beta-1-binding protein 1 | ITGB1BP1 | 2 | 2 | 21,8 | -0,54 | 0,134 | -0,28 | 0,510 |
| Q13418 | Integrin-linked protein kinase | ILK | 2 | 2 | 51,4 | -0,12 | 0,234 | 0,00 | 0,879 |
| P05362 | Intercellular adhesion molecule 1 | ICAM1 | 6 | 6 | 57,8 | 0,55 | 0,199 | 0,05 | 0,830 |
| O14896 | Interferon regulatory factor 6 | IRF6 | 1 | 1 | 53,1 | 0,25 | 0,044 | 0,45 | 0,199 |
| P13164 | Interferon-induced transmembrane protein 1 | IFITM1 | 1 | 1 | 14,0 | -0,48 | 0,167 | -0,83 | 0,053 |
| Q9NPH3 | Interleukin-1 receptor accessory protein | IL1RAP | 1 | 1 | 65,4 | -0,32 | 0,443 | 0,47 | 0,300 |
| P18510 | Interleukin-1 receptor antagonist protein | IL1RN | 1 | 1 | 20,0 | -0,27 | 0,136 | 0,03 | 0,954 |
| Q14116 | Interleukin-18 | IL18 | 1 | 1 | 22,3 | 0,22 | 0,305 | 1,04 | 0,192 |
| Q9HBG6 | Intraflagellar transport protein 122 homolog | IFT122 | 3 | 3 | 141,7 | -0,04 | 0,839 | 0,40 | 0,607 |
| Q96RY7 | Intraflagellar transport protein 140 homolog | IFT140 | 6 | 6 | 165,1 | 0,03 | 0,459 | 0,05 | 0,413 |
| Q9UG01 | Intraflagellar transport protein 172 homolog | IFT172 | 5 | 5 | 197,5 | 0,25 | 0,002 | 0,66 | 0,024 |
| Q9NQC8 | Intraflagellar transport protein 46 homolog | IFT46 | 1 | 1 | 34,3 | 0,47 | 0,065 | -0,11 | 0,495 |
| Q9Y366 | Intraflagellar transport protein 52 homolog | IFT52 | 1 | 1 | 49,7 | 0,57 | 0,239 | 0,20 | 0,444 |
| A0AVF1 | Intraflagellar transport protein 56 | TTC26 | 2 | 2 | 64,1 | 0,35 | 0,152 | 0,27 | 0,917 |
| Q9NWB7 | Intraflagellar transport protein 57 homolog | IFT57 | 1 | 1 | 49,1 | 0,75 | 0,882 | 0,21 | 0,828 |
| Q96LB3 | Intraflagellar transport protein 74 homolog | IFT74 | 3 | 3 | 69,2 | 0,05 | 0,714 | 0,28 | 0,375 |
| Q9P2H3 | Intraflagellar transport protein 80 homolog | IFT80 | 2 | 2 | 88,0 | -0,32 | 0,435 | 0,02 | 0,999 |
| Q8WYA0 | Intraflagellar transport protein 81 homolog | IFT81 | 3 | 3 | 79,7 | 0,57 | 0,117 | 0,36 | 0,260 |
| Q13099 | Intraflagellar transport protein 88 homolog | IFT88 | 1 | 1 | 94,2 | 0,18 | 0,044 | 0,13 | 0,618 |
| Q8NA54 | IQ and ubiquitin-like domain-containing protein | IQUB | 1 | 1 | 92,5 | -1,30 | 0,300 | -1,28 | 0,602 |
| Q6DN90 | IQ motif and SEC7 domain-containing protein 1 | IQSEC1 | 1 | 1 | 108,2 | -0,02 | 0,812 | -0,17 | 0,152 |
| Q96CN7 | Isochorismatase domain-containing protein 1 | ISOC1 | 1 | 1 | 32,2 | 0,37 | 0,340 | -0,10 | 0,851 |
| O75874 | Isocitrate dehydrogenase [NADP] cytoplasmic | IDH1 | 4 | 4 | 46,6 | -0,08 | 0,561 | 0,25 | 0,160 |
| P53990 | IST1 homolog | IST1 | 3 | 3 | 39,7 | -0,06 | 0,926 | 0,02 | 0,767 |
| P14923 | Junction plakoglobin | JUP | 8 | 8 | 81,7 | 1,47 | 0,579 | 0,70 | 0,475 |
| Q9Y624 | Junctional adhesion molecule A | F11R | 3 | 3 | 32,6 | 0,34 | 0,275 | -0,39 | 0,198 |
| O60229 | Kalirin | KALRN | 2 | 2 | 340,0 | -0,55 | 0,192 | -0,46 | 0,118 |
| Q14525 | Keratin, type I cuticular Ha3-II | KRT33B | 1 | 2 | 46,2 | -0,45 | 0,934 | 1,35 | 0,404 |
| P13645 | Keratin, type I cytoskeletal 10 | KRT10 | 11 | 16 | 58,8 | 0,20 | 0,975 | 0,33 | 0,746 |
| P13646 | Keratin, type I cytoskeletal 13 | KRT13 | 6 | 11 | 49,6 | 1,21 | 0,188 | 1,40 | 0,071 |
| P02533 | Keratin, type I cytoskeletal 14 | KRT14 | 6 | 18 | 51,5 | 1,98 | 0,630 | 0,20 | 0,809 |
| P08779 | Keratin, type I cytoskeletal 16 | KRT16 | 9 | 17 | 51,2 | 3,26 | 0,441 | 0,35 | 0,749 |
| Q04695 | Keratin, type I cytoskeletal 17 | KRT17 | 10 | 17 | 48,1 | 2,78 | 0,481 | 0,27 | 0,749 |
| P08727 | Keratin, type I cytoskeletal 19 | KRT19 | 7 | 12 | 44,1 | -0,22 | 0,594 | -0,47 | 0,023 |
| Q7Z3Y8 | Keratin, type I cytoskeletal 27 | KRT27 | 4 | 7 | 49,8 | -1,31 | 0,339 | 0,19 | 0,800 |
| P35527 | Keratin, type I cytoskeletal 9 | KRT9 | 15 | 15 | 62,0 | 0,93 | 0,817 | 0,29 | 0,745 |
| P78386 | Keratin, type II cuticular Hb5 | KRT85 | 1 | 3 | 55,8 | 1,29 | 0,035 | 1,87 | 0,182 |
| O43790 | Keratin, type II cuticular Hb6 | KRT86 | 2 | 3 | 53,5 | 0,14 | 0,575 | 1,73 | 0,333 |
| P04264 | Keratin, type II cytoskeletal 1 | KRT1 | 20 | 24 | 66,0 | 1,02 | 0,761 | 0,37 | 0,731 |
| Q7Z794 | Keratin, type II cytoskeletal 1b | KRT77 | 6 | 7 | 61,9 | -0,80 | 0,046 | -0,14 | 0,786 |
| P35908 | Keratin, type II cytoskeletal 2 epidermal | KRT2 | 15 | 21 | 65,4 | -0,70 | 0,030 | 0,45 | 0,695 |
| Q01546 | Keratin, type II cytoskeletal 2 oral | KRT76 | 1 | 5 | 65,8 | 0,19 | 0,553 | -0,27 | 0,027 |
| P19013 | Keratin, type II cytoskeletal 4 | KRT4 | 4 | 7 | 57,2 | 0,32 | 0,646 | 0,53 | 0,462 |
| P13647 | Keratin, type II cytoskeletal 5 | KRT5 | 12 | 16 | 62,3 | 1,23 | 0,622 | 0,30 | 0,711 |
| P02538 | Keratin, type II cytoskeletal 6A | KRT6A | 2 | 17 | 60,0 | 2,37 | 0,382 | -0,11 | 0,729 |
| P04259 | Keratin, type II cytoskeletal 6B | KRT6B | 1 | 15 | 60,0 | 2,55 | 0,351 | -0,05 | 0,881 |
| P48668 | Keratin, type II cytoskeletal 6C | KRT6C | 1 | 15 | 60,0 | 4,24 | 0,306 | 0,55 | 0,398 |
| P08729 | Keratin, type II cytoskeletal 7 | KRT7 | 3 | 4 | 51,4 | -0,19 | 0,930 | 0,20 | 0,313 |
| Q3SY84 | Keratin, type II cytoskeletal 71 | KRT71 | 6 | 7 | 57,3 | -1,00 | 0,225 | 0,37 | 0,725 |
| Q8N1N4 | Keratin, type II cytoskeletal 78 | KRT78 | 9 | 11 | 56,8 | 0,75 | 0,754 | 0,38 | 0,637 |
| P05787 | Keratin, type II cytoskeletal 8 | KRT8 | 2 | 4 | 53,7 | 0,02 | 0,673 | 0,14 | 0,521 |
| Q6KB66 | Keratin, type II cytoskeletal 80 | KRT80 | 4 | 5 | 50,5 | 1,25 | 0,650 | 0,23 | 0,857 |
| Q5T749 | Keratinocyte proline-rich protein | KPRP | 2 | 2 | 64,1 | 1,07 | 0,633 | 1,27 | 0,256 |
| Q14894 | Ketimine reductase mu-crystallin | CRYM | 2 | 2 | 33,8 | 0,77 | 0,077 | -0,11 | 0,659 |
| Q9HA64 | Ketosamine-3-kinase | FN3KRP | 1 | 1 | 34,4 | -0,04 | 0,715 | 0,18 | 0,075 |
| Q07866 | Kinesin light chain 1 | KLC1 | 1 | 1 | 65,3 | -0,12 | 0,680 | -0,14 | 0,702 |
| P33176 | Kinesin-1 heavy chain | KIF5B | 5 | 5 | 109,6 | -0,12 | 0,943 | -0,05 | 0,938 |
| Q92845 | Kinesin-associated protein 3 | KIFAP3 | 1 | 1 | 91,1 | -0,72 | 0,655 | -0,87 | 0,419 |
| Q2TAC6 | Kinesin-like protein KIF19 | KIF19 | 1 | 1 | 111,3 | -0,53 | 0,939 | 0,06 | 0,207 |
| Q7Z4S6 | Kinesin-like protein KIF21A | KIF21A | 1 | 1 | 187,1 | -0,03 | 0,807 | 0,49 | 0,302 |
| Q86VH2 | Kinesin-like protein KIF27 | KIF27 | 2 | 2 | 160,2 | -0,08 | 0,870 | -0,06 | 0,392 |
| O00139 | Kinesin-like protein KIF2A | KIF2A | 3 | 3 | 79,9 | -0,53 | 0,399 | 0,08 | 0,824 |
| O00522 | Krev interaction trapped protein 1 | KRIT1 | 4 | 4 | 84,3 | -0,51 | 0,029 | -0,12 | 0,182 |
| Q08431 | Lactadherin | MFGE8 | 3 | 3 | 43,1 | 0,07 | 0,481 | 0,16 | 0,514 |
| P02788 | Lactotransferrin | LTF | 2 | 2 | 78,1 | 0,06 | 0,574 | 0,65 | 0,319 |
| Q04760 | Lactoylglutathione lyase | GLO1 | 1 | 1 | 20,8 | 0,15 | 0,423 | 0,15 | 0,523 |
| Q16787 | Laminin subunit alpha-3 | LAMA3 | 3 | 3 | 366,4 | -0,19 | 0,926 | -0,05 | 0,674 |
| O15230 | Laminin subunit alpha-5 | LAMA5 | 1 | 1 | 399,5 | 0,20 | 0,968 | 0,81 | 0,171 |
| Q13751 | Laminin subunit beta-3 | LAMB3 | 4 | 4 | 129,5 | -0,17 | 0,569 | -0,32 | 0,197 |
| P11047 | Laminin subunit gamma-1 | LAMC1 | 1 | 1 | 177,5 | 0,55 | 0,197 | 0,86 | 0,043 |
| Q13753 | Laminin subunit gamma-2 | LAMC2 | 7 | 7 | 130,9 | 0,26 | 0,372 | -0,29 | 0,804 |
| Q86VQ0 | Lebercilin | LCA5 | 2 | 2 | 80,5 | -0,98 | 0,075 | -0,57 | 0,241 |
| O60299 | Leucine zipper putative tumor suppressor 3 | LZTS3 | 1 | 1 | 71,7 | 1,11 | 0,009 | 0,25 | 0,733 |
| Q9NQ48 | Leucine zipper transcription factor-like protein 1 | LZTFL1 | 5 | 5 | 34,6 | 0,11 | 0,443 | -0,64 | 0,385 |
| Q96FV0 | Leucine-rich repeat-containing protein 46 | LRRC46 | 2 | 2 | 35,3 | -0,52 | 0,464 | -0,28 | 0,652 |
| Q8N1G4 | Leucine-rich repeat-containing protein 47 | LRRC47 | 2 | 2 | 63,4 | 0,09 | 0,356 | 0,15 | 0,792 |
| Q8N9N7 | Leucine-rich repeat-containing protein 57 | LRRC57 | 2 | 2 | 26,7 | 0,09 | 0,624 | -0,10 | 0,951 |
| P30740 | Leukocyte elastase inhibitor | SERPINB1 | 4 | 4 | 42,7 | -0,13 | 0,263 | -0,23 | 0,057 |
| Q08722 | Leukocyte surface antigen CD47 | CD47 | 2 | 2 | 35,2 | 0,25 | 0,049 | -0,18 | 0,069 |
| P09960 | Leukotriene A-4 hydrolase | LTA4H | 2 | 2 | 69,2 | -0,15 | 0,388 | 0,00 | 0,854 |
| P48059 | LIM and senescent cell antigen-like-containing domain protein 1 | LIMS1 | 1 | 1 | 37,2 | 0,02 | 0,856 | -0,56 | 0,323 |
| Q14847 | LIM and SH3 domain protein 1 | LASP1 | 4 | 4 | 29,7 | -0,40 | 0,077 | -0,04 | 0,912 |
| Q9UHB6 | LIM domain and actin-binding protein 1 | LIMA1 | 5 | 5 | 85,2 | -0,05 | 0,844 | -0,32 | 0,513 |
| Q8WWI1 | LIM domain only protein 7 | LMO7 | 1 | 1 | 192,6 | 1,02 | 0,012 | 1,06 | 0,104 |
| Q86X29 | Lipolysis-stimulated lipoprotein receptor | LSR | 5 | 5 | 71,4 | -0,31 | 0,430 | -0,29 | 0,667 |
| Q93052 | Lipoma-preferred partner | LPP | 1 | 1 | 65,7 | -0,40 | 0,102 | -0,20 | 0,682 |
| P50851 | Lipopolysaccharide-responsive and beige-like anchor protein | LRBA | 4 | 4 | 318,9 | 1,34 | 0,045 | -0,05 | 0,997 |
| P00338 | L-lactate dehydrogenase A chain | LDHA | 4 | 6 | 36,7 | -0,34 | 0,250 | -0,04 | 0,522 |
| P07195 | L-lactate dehydrogenase B chain | LDHB | 3 | 5 | 36,6 | 0,11 | 0,528 | 0,01 | 0,993 |
| P23490 | Loricrin | LOR | 1 | 1 | 25,7 | 0,38 | 0,918 | 1,04 | 0,522 |
| Q6UXB3 | Ly6/PLAUR domain-containing protein 2 | LYPD2 | 1 | 1 | 13,1 | -0,70 | 0,356 | -0,56 | 0,333 |
| Q14210 | Lymphocyte antigen 6D | LY6D | 1 | 1 | 13,3 | -0,26 | 0,822 | -0,71 | 0,043 |
| P19256 | Lymphocyte function-associated antigen 3 | CD58 | 2 | 2 | 28,1 | -0,07 | 0,638 | 0,12 | 0,850 |
| Q14108 | Lysosome membrane protein 2 | SCARB2 | 1 | 1 | 54,3 | 0,53 | 0,181 | 0,27 | 0,153 |
| P11279 | Lysosome-associated membrane glycoprotein 1 | LAMP1 | 2 | 2 | 44,9 | 0,24 | 0,373 | -0,21 | 0,317 |
| P61626 | Lysozyme C | LYZ | 3 | 3 | 16,5 | -0,98 | 0,185 | 0,62 | 0,306 |
| P14174 | Macrophage migration inhibitory factor | MIF | 1 | 1 | 12,5 | -0,15 | 0,074 | 0,02 | 0,951 |
| P40121 | Macrophage-capping protein | CAPG | 2 | 2 | 38,5 | 0,06 | 0,551 | 0,22 | 0,150 |
| Q8N3R9 | MAGUK p55 subfamily member 5 | MPP5 | 6 | 6 | 77,2 | 0,00 | 0,938 | 0,15 | 0,241 |
| P04156 | Major prion protein | PRNP | 1 | 1 | 27,6 | -1,11 | 0,344 | -0,84 | 0,369 |
| Q14764 | Major vault protein | MVP | 6 | 6 | 99,3 | 0,48 | 0,067 | 0,51 | 0,359 |
| P40925 | Malate dehydrogenase, cytoplasmic | MDH1 | 4 | 4 | 36,4 | 0,05 | 0,528 | 0,12 | 0,185 |
| P40926 | Malate dehydrogenase, mitochondrial | MDH2 | 1 | 1 | 35,5 | 0,22 | 0,550 | -0,03 | 0,660 |
| Q9ULC4 | Malignant T-cell-amplified sequence 1 | MCTS1 | 1 | 1 | 20,5 | -0,14 | 0,237 | -0,08 | 0,043 |
| Q9Y5P6 | Mannose-1-phosphate guanyltransferase beta | GMPPB | 1 | 1 | 39,8 | 0,30 | 0,028 | 0,24 | 0,132 |
| P49006 | MARCKS-related protein | MARCKSL1 | 4 | 4 | 19,5 | -0,66 | 0,016 | -0,31 | 0,406 |
| Q5HYA8 | Meckelin | TMEM67 | 3 | 3 | 111,7 | -0,01 | 0,635 | 0,20 | 0,487 |
| Q9UNF1 | Melanoma-associated antigen D2 | MAGED2 | 1 | 1 | 64,9 | 0,19 | 0,140 | 0,20 | 0,692 |
| P35240 | Merlin | NF2 | 1 | 1 | 69,6 | -0,38 | 0,236 | -0,31 | 0,241 |
| Q9H1K6 | Mesoderm development candidate 1 | MESDC1 | 2 | 2 | 37,7 | -0,44 | 0,181 | 0,03 | 0,883 |
| Q13421 | Mesothelin | MSLN | 4 | 4 | 68,9 | 0,12 | 0,979 | -0,09 | 0,844 |
| Q687X5 | Metalloreductase STEAP4 | STEAP4 | 2 | 2 | 51,9 | -1,00 | 0,027 | -0,43 | 0,091 |
| P50579 | Methionine aminopeptidase 2 | METAP2 | 1 | 1 | 52,9 | -0,18 | 0,604 | 0,33 | 0,171 |
| P56192 | Methionine--tRNA ligase, cytoplasmic | MARS | 1 | 1 | 101,1 | 0,66 | 0,179 | 0,60 | 0,496 |
| Q8N3F8 | MICAL-like protein 1 | MICALL1 | 1 | 1 | 93,4 | -0,07 | 0,073 | -0,92 | 0,151 |
| Q8IY33 | MICAL-like protein 2 | MICALL2 | 3 | 3 | 97,4 | 0,19 | 0,565 | -0,16 | 0,404 |
| P27816 | Microtubule-associated protein 4 | MAP4 | 1 | 1 | 120,9 | -0,81 | 0,996 | 0,72 | 0,140 |
| P21741 | Midkine | MDK | 1 | 1 | 15,6 | -0,04 | 0,913 | 0,27 | 0,859 |
| Q8N4C8 | Misshapen-like kinase 1 | MINK1 | 3 | 3 | 149,7 | -0,21 | 0,500 | 0,00 | 0,992 |
| Q8TC71 | Mitochondria-eating protein | SPATA18 | 1 | 1 | 61,1 | 1,07 | 0,015 | 1,55 | 0,149 |
| P28482 | Mitogen-activated protein kinase 1 | MAPK1 | 1 | 1 | 41,4 | 0,15 | 0,620 | -0,70 | 0,405 |
| P27361 | Mitogen-activated protein kinase 3 | MAPK3 | 1 | 1 | 43,1 | 0,02 | 0,791 | -0,21 | 0,263 |
| Q8IVT2 | Mitotic interactor and substrate of PLK1 | MISP | 8 | 8 | 75,3 | 0,10 | 0,670 | -0,33 | 0,180 |
| P26038 | Moesin | MSN | 13 | 17 | 67,8 | -0,44 | 0,124 | -0,08 | 0,639 |
| O15427 | Monocarboxylate transporter 4 | SLC16A3 | 1 | 1 | 49,4 | 0,32 | 0,586 | -0,29 | 0,424 |
| P08571 | Monocyte differentiation antigen CD14 | CD14 | 3 | 3 | 40,1 | -0,68 | 0,165 | -0,47 | 0,228 |
| Q15797 | Mothers against decapentaplegic homolog 1 | SMAD1 | 2 | 2 | 52,2 | -0,19 | 0,679 | 0,23 | 0,672 |
| Q15796 | Mothers against decapentaplegic homolog 2 | SMAD2 | 2 | 2 | 52,3 | -0,45 | 0,326 | -0,09 | 0,881 |
| P84022 | Mothers against decapentaplegic homolog 3 | SMAD3 | 1 | 1 | 48,0 | -0,02 | 0,980 | -0,25 | 0,097 |
| Q9H7C9 | Mth938 domain-containing protein | AAMDC | 1 | 1 | 13,3 | -0,53 | 0,462 | -0,20 | 0,807 |
| P15941 | Mucin-1 | MUC1 | 4 | 4 | 122,0 | -0,40 | 0,360 | -0,30 | 0,040 |
| Q9H3R2 | Mucin-13 | MUC13 | 2 | 2 | 54,6 | 1,33 | 0,088 | 0,29 | 0,942 |
| Q8WXI7 | Mucin-16 | MUC16 | 22 | 22 | 1518,2 | -0,64 | 0,327 | -0,06 | 0,877 |
| Q8N307 | Mucin-20 | MUC20 | 1 | 1 | 71,9 | 0,27 | 0,318 | -0,13 | 0,348 |
| Q99102 | Mucin-4 | MUC4 | 4 | 4 | 231,4 | 0,16 | 0,475 | -0,37 | 0,114 |
| P98088 | Mucin-5AC | MUC5AC | 32 | 33 | 585,2 | -2,18 | 0,467 | -0,73 | 0,697 |
| Q9HC84 | Mucin-5B | MUC5B | 24 | 25 | 596,0 | -1,83 | 0,610 | -1,18 | 0,840 |
| Q6W4X9 | Mucin-6 | MUC6 | 2 | 2 | 256,9 | -2,38 | 0,544 | 0,45 | 0,223 |
| P22234 | Multifunctional protein ADE2 | PAICS | 1 | 1 | 47,0 | -0,40 | 0,370 | -0,35 | 0,255 |
| Q96EY5 | Multivesicular body subunit 12A | MVB12A | 1 | 1 | 28,8 | -0,10 | 0,336 | 0,45 | 0,560 |
| P02686 | Myelin basic protein | MBP | 2 | 2 | 33,1 | -0,59 | 0,287 | -0,83 | 0,008 |
| Q9NZM1 | Myoferlin | MYOF | 23 | 23 | 234,6 | 0,02 | 0,973 | 0,00 | 0,923 |
| P60660 | Myosin light polypeptide 6 | MYL6 | 3 | 3 | 16,9 | 0,26 | 0,080 | 0,10 | 0,857 |
| Q7Z406 | Myosin-14 | MYH14 | 6 | 6 | 227,7 | 0,79 | 0,038 | 0,15 | 0,492 |
| P35579 | Myosin-9 | MYH9 | 7 | 7 | 226,4 | -0,02 | 0,849 | 0,00 | 0,827 |
| P58546 | Myotrophin | MTPN | 1 | 1 | 12,9 | -0,14 | 0,750 | -0,24 | 0,813 |
| Q13496 | Myotubularin | MTM1 | 2 | 2 | 69,9 | 0,23 | 0,293 | 0,01 | 0,709 |
| Q13613 | Myotubularin-related protein 1 | MTMR1 | 3 | 3 | 74,6 | -0,18 | 0,616 | 0,19 | 0,570 |
| O95248 | Myotubularin-related protein 5 | SBF1 | 6 | 6 | 208,2 | -0,43 | 0,033 | -0,04 | 0,759 |
| P29966 | Myristoylated alanine-rich C-kinase substrate | MARCKS | 2 | 2 | 31,5 | -0,48 | 0,080 | -0,20 | 0,552 |
| O94760 | N(G),N(G)-dimethylarginine dimethylaminohydrolase 1 | DDAH1 | 1 | 1 | 31,1 | -0,07 | 0,702 | -0,19 | 0,003 |
| O95865 | N(G),N(G)-dimethylarginine dimethylaminohydrolase 2 | DDAH2 | 2 | 2 | 29,6 | -0,14 | 0,248 | -0,19 | 0,189 |
| O14745 | Na(+)/H(+) exchange regulatory cofactor NHE-RF1 | SLC9A3R1 | 15 | 15 | 38,8 | -0,44 | 0,103 | -0,49 | 0,172 |
| Q15599 | Na(+)/H(+) exchange regulatory cofactor NHE-RF2 | SLC9A3R2 | 7 | 7 | 37,4 | -0,25 | 0,288 | 0,17 | 0,071 |
| Q86UT5 | Na(+)/H(+) exchange regulatory cofactor NHE-RF4 | PDZD3 | 1 | 1 | 61,0 | 1,70 | 0,583 | 0,39 | 0,552 |
| Q9UJ70 | N-acetyl-D-glucosamine kinase | NAGK | 1 | 1 | 37,4 | -0,17 | 0,431 | -0,19 | 0,515 |
| Q86SF2 | N-acetylgalactosaminyltransferase 7 | GALNT7 | 1 | 1 | 75,3 | 0,18 | 0,362 | 0,73 | 0,015 |
| Q9Y2A9 | N-acetyllactosaminide beta-1,3-N-acetylglucosaminyltransferase 3 | B3GNT3 | 1 | 1 | 42,5 | 0,05 | 0,568 | -0,60 | 0,715 |
| P15559 | NAD(P)H dehydrogenase [quinone] 1 | NQO1 | 3 | 3 | 30,8 | -0,29 | 0,598 | -0,20 | 0,779 |
| P48163 | NADP-dependent malic enzyme | ME1 | 1 | 1 | 64,1 | 0,51 | 0,159 | 0,58 | 0,292 |
| Q8NFA2 | NADPH oxidase organizer 1 | NOXO1 | 1 | 1 | 41,2 | 0,73 | 0,403 | 0,37 | 0,710 |
| Q13765 | Nascent polypeptide-associated complex subunit alpha | NACA | 2 | 2 | 23,4 | 0,11 | 0,659 | 0,58 | 0,207 |
| P49281 | Natural resistance-associated macrophage protein 2 | SLC11A2 | 1 | 1 | 62,2 | 0,39 | 0,001 | -0,41 | 0,021 |
| Q9Y2A7 | Nck-associated protein 1 | NCKAP1 | 3 | 3 | 128,7 | 0,08 | 0,907 | -0,27 | 0,386 |
| O76041 | Nebulette | NEBL | 2 | 2 | 116,4 | -0,19 | 0,688 | -0,11 | 0,547 |
| Q9H0M0 | NEDD4-like E3 ubiquitin-protein ligase WWP1 | WWP1 | 1 | 1 | 105,1 | 0,02 | 0,897 | 0,22 | 0,224 |
| O00308 | NEDD4-like E3 ubiquitin-protein ligase WWP2 | WWP2 | 2 | 2 | 98,9 | -0,34 | 0,274 | -0,55 | 0,106 |
| P61081 | NEDD8-conjugating enzyme Ubc12 | UBE2M | 1 | 1 | 20,9 | -0,05 | 0,897 | 0,16 | 0,612 |
| O00401 | Neural Wiskott-Aldrich syndrome protein | WASL | 1 | 1 | 54,8 | -0,18 | 0,504 | -0,17 | 0,670 |
| Q8NFP9 | Neurobeachin | NBEA | 2 | 2 | 327,6 | 0,97 | 0,016 | 0,38 | 0,747 |
| Q09666 | Neuroblast differentiation-associated protein AHNAK | AHNAK | 25 | 25 | 628,7 | -0,26 | 0,327 | 0,03 | 0,950 |
| P62166 | Neuronal calcium sensor 1 | NCS1 | 1 | 1 | 21,9 | 0,20 | 0,786 | -0,89 | 0,234 |
| Q15758 | Neutral amino acid transporter B(0) | SLC1A5 | 2 | 2 | 56,6 | -0,25 | 0,379 | 0,39 | 0,361 |
| P80188 | Neutrophil gelatinase-associated lipocalin | LCN2 | 1 | 1 | 22,6 | 1,31 | 0,084 | -0,64 | 0,479 |
| Q8NBF2 | NHL repeat-containing protein 2 | NHLRC2 | 1 | 1 | 79,4 | 0,61 | 0,069 | 0,04 | 0,997 |
| Q96TA1 | Niban-like protein 1 | FAM129B | 4 | 4 | 84,1 | -0,02 | 0,968 | 0,04 | 0,977 |
| Q92542 | Nicastrin | NCSTN | 1 | 1 | 78,4 | 0,18 | 0,189 | 0,36 | 0,184 |
| P43490 | Nicotinamide phosphoribosyltransferase | NAMPT | 2 | 2 | 55,5 | 0,16 | 0,791 | 0,50 | 0,062 |
| Q6XQN6 | Nicotinate phosphoribosyltransferase | NAPRT | 1 | 1 | 57,5 | 0,38 | 0,399 | 0,02 | 0,994 |
| Q9GZT8 | NIF3-like protein 1 | NIF3L1 | 1 | 1 | 41,9 | 0,42 | 0,084 | -0,07 | 0,767 |
| P35228 | Nitric oxide synthase, inducible | NOS2 | 8 | 8 | 131,0 | 0,95 | 0,190 | 4,44 | 0,049 |
| Q15233 | Non-POU domain-containing octamer-binding protein | NONO | 1 | 1 | 54,2 | 0,23 | 0,390 | 0,61 | 0,291 |
| Q9UNZ2 | NSFL1 cofactor p47 | NSFL1C | 1 | 1 | 40,5 | -0,46 | 0,531 | -0,98 | 0,027 |
| P19838 | Nuclear factor NF-kappa-B p105 subunit | NFKB1 | 1 | 1 | 105,3 | 0,51 | 0,237 | 0,37 | 0,193 |
| Q9Y266 | Nuclear migration protein nudC | NUDC | 3 | 3 | 38,2 | 0,09 | 0,418 | -0,19 | 0,075 |
| Q8N1F7 | Nuclear pore complex protein Nup93 | NUP93 | 1 | 1 | 93,4 | -0,30 | 0,106 | 0,09 | 0,986 |
| Q02818 | Nucleobindin-1 | NUCB1 | 1 | 1 | 53,8 | 0,53 | 0,288 | 0,09 | 0,452 |
| Q9NTK5 | Obg-like ATPase 1 | OLA1 | 2 | 2 | 44,7 | -0,13 | 0,384 | 0,19 | 0,018 |
| Q9Y3B8 | Oligoribonuclease, mitochondrial | REXO2 | 1 | 1 | 26,8 | 0,26 | 0,440 | -0,23 | 0,715 |
| Q9NQR4 | Omega-amidase NIT2 | NIT2 | 3 | 3 | 30,6 | 0,00 | 0,877 | -0,05 | 0,762 |
| Q92882 | Osteoclast-stimulating factor 1 | OSTF1 | 1 | 1 | 23,8 | -0,07 | 0,838 | -0,40 | 0,405 |
| Q6GQQ9 | OTU domain-containing protein 7B | OTUD7B | 3 | 3 | 92,5 | -0,42 | 0,222 | -0,59 | 0,091 |
| A8MYP8 | Outer dense fiber protein 3B | ODF3B | 3 | 3 | 27,3 | -0,64 | 0,627 | 0,87 | 0,375 |
| P41231 | P2Y purinoceptor 2 | P2RY2 | 2 | 2 | 42,2 | -0,13 | 0,368 | -0,01 | 0,846 |
| Q9NVE7 | Pantothenate kinase 4 | PANK4 | 1 | 1 | 85,9 | 1,18 | 0,631 | 0,38 | 0,573 |
| O75781 | Paralemmin-1 | PALM | 4 | 4 | 42,1 | 0,02 | 0,723 | -0,13 | 0,905 |
| Q9Y365 | PCTP-like protein | STARD10 | 1 | 1 | 33,0 | -0,27 | 0,349 | -0,12 | 0,118 |
| O00151 | PDZ and LIM domain protein 1 | PDLIM1 | 2 | 2 | 36,0 | 0,07 | 0,797 | 0,05 | 0,860 |
| O14908 | PDZ domain-containing protein GIPC1 | GIPC1 | 3 | 3 | 36,0 | -0,10 | 0,558 | -0,17 | 0,420 |
| Q13113 | PDZK1-interacting protein 1 | PDZK1IP1 | 1 | 1 | 12,2 | 1,88 | 0,035 | 0,20 | 0,641 |
| Q9UBV8 | Peflin | PEF1 | 1 | 1 | 30,4 | 0,22 | 0,183 | 0,46 | 0,178 |
| O43511 | Pendrin | SLC26A4 | 4 | 4 | 85,7 | 4,07 | 0,021 | 2,79 | 0,038 |
| P62937 | Peptidyl-prolyl cis-trans isomerase A | PPIA | 5 | 5 | 18,0 | -0,10 | 0,961 | -0,10 | 0,978 |
| P23284 | Peptidyl-prolyl cis-trans isomerase B | PPIB | 2 | 2 | 23,7 | 0,70 | 0,085 | 0,07 | 0,399 |
| P45877 | Peptidyl-prolyl cis-trans isomerase C | PPIC | 1 | 1 | 22,7 | 0,38 | 0,082 | 0,13 | 0,961 |
| P62942 | Peptidyl-prolyl cis-trans isomerase FKBP1A | FKBP1A | 1 | 1 | 11,9 | -0,37 | 0,374 | -0,27 | 0,239 |
| Q02790 | Peptidyl-prolyl cis-trans isomerase FKBP4 | FKBP4 | 1 | 1 | 51,8 | 1,17 | 0,019 | 0,96 | 0,070 |
| Q9Y237 | Peptidyl-prolyl cis-trans isomerase NIMA-interacting 4 | PIN4 | 1 | 1 | 13,8 | -0,38 | 0,083 | 0,05 | 0,989 |
| O60664 | Perilipin-3 | PLIN3 | 3 | 3 | 47,0 | 0,13 | 0,391 | -0,23 | 0,275 |
| O60437 | Periplakin | PPL | 21 | 21 | 204,6 | 0,19 | 0,413 | 0,05 | 0,858 |
| Q06830 | Peroxiredoxin-1 | PRDX1 | 5 | 6 | 22,1 | 0,36 | 0,032 | 0,37 | 0,089 |
| P32119 | Peroxiredoxin-2 | PRDX2 | 3 | 4 | 21,9 | 0,06 | 0,616 | 0,13 | 0,060 |
| P30044 | Peroxiredoxin-5, mitochondrial | PRDX5 | 1 | 1 | 22,1 | 0,07 | 0,297 | -0,42 | 0,447 |
| P30041 | Peroxiredoxin-6 | PRDX6 | 4 | 4 | 25,0 | 0,18 | 0,004 | -0,31 | 0,100 |
| O60346 | PH domain leucine-rich repeat-containing protein phosphatase 1 | PHLPP1 | 1 | 1 | 184,6 | -0,74 | 0,094 | -0,60 | 0,143 |
| O75167 | Phosphatase and actin regulator 2 | PHACTR2 | 2 | 2 | 69,7 | -0,27 | 0,135 | -0,03 | 0,895 |
| Q8IZ21 | Phosphatase and actin regulator 4 | PHACTR4 | 2 | 2 | 78,2 | -0,51 | 0,139 | -0,07 | 0,705 |
| P30086 | Phosphatidylethanolamine-binding protein 1 | PEBP1 | 2 | 2 | 21,0 | 0,10 | 0,427 | -0,08 | 0,933 |
| P42356 | Phosphatidylinositol 4-kinase alpha | PI4KA | 6 | 6 | 236,7 | -0,28 | 0,146 | 0,34 | 0,013 |
| O60331 | Phosphatidylinositol 4-phosphate 5-kinase type-1 gamma | PIP5K1C | 2 | 2 | 73,2 | 0,05 | 0,837 | 0,40 | 0,193 |
| P48426 | Phosphatidylinositol 5-phosphate 4-kinase type-2 alpha | PIP4K2A | 1 | 1 | 46,2 | 0,74 | 0,092 | 0,23 | 0,784 |
| P78356 | Phosphatidylinositol 5-phosphate 4-kinase type-2 beta | PIP4K2B | 1 | 1 | 47,3 | -0,09 | 0,840 | 0,08 | 0,857 |
| Q00169 | Phosphatidylinositol transfer protein alpha isoform | PITPNA | 1 | 1 | 31,8 | -0,31 | 0,010 | -0,06 | 0,667 |
| Q13492 | Phosphatidylinositol-binding clathrin assembly protein | PICALM | 1 | 1 | 70,7 | 0,43 | 0,036 | -0,05 | 0,670 |
| O95394 | Phosphoacetylglucosamine mutase | PGM3 | 2 | 2 | 59,8 | 0,88 | 0,204 | 0,64 | 0,509 |
| Q6VY07 | Phosphofurin acidic cluster sorting protein 1 | PACS1 | 3 | 3 | 104,8 | -0,27 | 0,192 | -0,09 | 0,464 |
| P36871 | Phosphoglucomutase-1 | PGM1 | 3 | 3 | 61,4 | 0,22 | 0,481 | 0,21 | 0,861 |
| Q96G03 | Phosphoglucomutase-2 | PGM2 | 2 | 2 | 68,2 | -0,02 | 0,797 | 0,11 | 0,450 |
| P00558 | Phosphoglycerate kinase 1 | PGK1 | 5 | 5 | 44,6 | 0,00 | 0,948 | 0,24 | 0,230 |
| P18669 | Phosphoglycerate mutase 1 | PGAM1 | 2 | 2 | 28,8 | -0,26 | 0,269 | -0,07 | 0,614 |
| Q13393 | Phospholipase D1 | PLD1 | 6 | 6 | 124,1 | 0,01 | 0,917 | 0,12 | 0,045 |
| P36969 | Phospholipid hydroperoxide glutathione peroxidase, mitochondrial | GPX4 | 1 | 1 | 22,2 | 0,03 | 0,777 | 0,13 | 0,590 |
| O15162 | Phospholipid scramblase 1 | PLSCR1 | 3 | 3 | 35,0 | -0,04 | 0,857 | -0,33 | 0,121 |
| Q9NRQ2 | Phospholipid scramblase 4 | PLSCR4 | 1 | 1 | 37,0 | 0,01 | 0,788 | 0,25 | 0,418 |
| P55058 | Phospholipid transfer protein | PLTP | 1 | 1 | 54,7 | 0,77 | 0,032 | 0,40 | 0,016 |
| O43520 | Phospholipid-transporting ATPase IC | ATP8B1 | 5 | 5 | 143,6 | -0,32 | 0,170 | 0,14 | 0,213 |
| Q9HAB8 | Phosphopantothenate--cysteine ligase | PPCS | 2 | 2 | 34,0 | 0,44 | 0,241 | -0,18 | 0,829 |
| P36955 | Pigment epithelium-derived factor | SERPINF1 | 1 | 1 | 46,3 | -1,05 | 0,223 | -0,36 | 0,747 |
| O00625 | Pirin | PIR | 1 | 1 | 32,1 | -0,05 | 0,744 | 0,07 | 0,458 |
| Q13835 | Plakophilin-1 | PKP1 | 8 | 8 | 82,8 | 1,34 | 0,634 | 0,69 | 0,523 |
| Q99959 | Plakophilin-2 | PKP2 | 1 | 1 | 97,4 | -0,50 | 0,031 | -0,33 | 0,348 |
| Q9Y446 | Plakophilin-3 | PKP3 | 4 | 4 | 87,0 | 0,39 | 0,567 | 0,18 | 0,409 |
| P05120 | Plasminogen activator inhibitor 2 | SERPINB2 | 3 | 3 | 46,6 | 0,08 | 0,842 | 2,60 | 0,017 |
| Q9Y342 | Plasmolipin | PLLP | 1 | 1 | 20,0 | -0,54 | 0,260 | -0,14 | 0,641 |
| Q14651 | Plastin-1 | PLS1 | 3 | 3 | 70,2 | 0,42 | 0,340 | 0,23 | 0,396 |
| P13797 | Plastin-3 | PLS3 | 6 | 6 | 70,8 | 0,09 | 0,694 | -0,15 | 0,858 |
| P16671 | Platelet glycoprotein 4 | CD36 | 1 | 1 | 53,0 | -0,37 | 0,954 | 2,33 | 0,037 |
| Q15102 | Platelet-activating factor acetylhydrolase IB subunit gamma | PAFAH1B3 | 2 | 2 | 25,7 | 0,52 | 0,115 | -0,11 | 0,710 |
| Q9HB21 | Pleckstrin homology domain-containing family A member 1 | PLEKHA1 | 1 | 1 | 45,5 | 0,46 | 0,121 | -0,13 | 0,414 |
| Q9H4M7 | Pleckstrin homology domain-containing family A member 4 | PLEKHA4 | 2 | 2 | 85,3 | -0,54 | 0,412 | -0,56 | 0,505 |
| Q9HAU0 | Pleckstrin homology domain-containing family A member 5 | PLEKHA5 | 2 | 2 | 127,4 | -1,15 | 0,305 | -0,03 | 0,984 |
| Q9H8W4 | Pleckstrin homology domain-containing family F member 2 | PLEKHF2 | 1 | 1 | 27,8 | 0,13 | 0,579 | -0,14 | 0,639 |
| Q5SXH7 | Pleckstrin homology domain-containing family S member 1 | PLEKHS1 | 1 | 1 | 51,8 | 1,57 | 0,008 | 0,72 | 0,341 |
| Q9Y5J5 | Pleckstrin homology-like domain family A member 3 | PHLDA3 | 2 | 2 | 13,9 | -0,13 | 0,805 | 0,24 | 0,457 |
| Q15149 | Plectin | PLEC | 8 | 9 | 531,5 | 0,17 | 0,281 | 0,01 | 0,813 |
| O15031 | Plexin-B2 | PLXNB2 | 3 | 3 | 205,0 | 1,05 | 0,001 | 0,07 | 0,914 |
| O00592 | Podocalyxin | PODXL | 1 | 1 | 58,6 | 0,60 | 0,351 | 1,30 | 0,232 |
| Q460N5 | Poly [ADP-ribose] polymerase 14 | PARP14 | 1 | 1 | 202,7 | 0,22 | 0,458 | 0,78 | 0,064 |
| Q9UKK3 | Poly [ADP-ribose] polymerase 4 | PARP4 | 1 | 1 | 192,5 | 0,44 | 0,191 | 0,10 | 0,265 |
| Q8IXQ6 | Poly [ADP-ribose] polymerase 9 | PARP9 | 1 | 1 | 96,3 | -0,07 | 0,883 | 0,15 | 0,723 |
| Q15365 | Poly(rC)-binding protein 1 | PCBP1 | 2 | 5 | 37,5 | -0,14 | 0,093 | -0,16 | 0,086 |
| Q15366 | Poly(rC)-binding protein 2 | PCBP2 | 2 | 5 | 38,6 | -0,16 | 0,703 | -0,37 | 0,336 |
| P01833 | Polymeric immunoglobulin receptor | PIGR | 8 | 8 | 83,2 | 1,29 | 0,021 | 1,05 | 0,218 |
| Q10471 | Polypeptide N-acetylgalactosaminyltransferase 2 | GALNT2 | 2 | 2 | 64,7 | 0,01 | 0,690 | -0,05 | 0,715 |
| Q8N4A0 | Polypeptide N-acetylgalactosaminyltransferase 4 | GALNT4 | 1 | 1 | 66,6 | -0,06 | 0,688 | 0,04 | 0,925 |
| P0CG48 | Polyubiquitin-C | UBC | 3 | 3 | 77,0 | 0,39 | 0,123 | 0,24 | 0,345 |
| P54707 | Potassium-transporting ATPase alpha chain 2 | ATP12A | 19 | 21 | 115,4 | 1,62 | 0,006 | 1,70 | 0,104 |
| Q9UHV9 | Prefoldin subunit 2 | PFDN2 | 1 | 1 | 16,6 | 0,26 | 0,541 | -0,41 | 0,604 |
| P02545 | Prelamin-A/C | LMNA | 3 | 3 | 74,1 | 0,50 | 0,151 | 0,36 | 0,238 |
| P49768 | Presenilin-1 | PSEN1 | 2 | 2 | 52,6 | -1,15 | 0,223 | 0,03 | 0,635 |
| Q9H7F0 | Probable cation-transporting ATPase 13A3 | ATP13A3 | 2 | 2 | 138,0 | -0,56 | 0,268 | -0,49 | 0,521 |
| Q4VNC1 | Probable cation-transporting ATPase 13A4 | ATP13A4 | 2 | 2 | 133,9 | 0,35 | 0,095 | 0,48 | 0,679 |
| Q86UW9 | Probable E3 ubiquitin-protein ligase DTX2 | DTX2 | 1 | 1 | 67,2 | -0,18 | 0,773 | -0,25 | 0,466 |
| Q9Y2G3 | Probable phospholipid-transporting ATPase IF | ATP11B | 1 | 1 | 134,1 | -0,47 | 0,170 | -0,79 | 0,103 |
| O75110 | Probable phospholipid-transporting ATPase IIA | ATP9A | 5 | 5 | 118,5 | -0,25 | 0,646 | -0,39 | 0,413 |
| O94823 | Probable phospholipid-transporting ATPase VB | ATP10B | 2 | 2 | 165,3 | 0,53 | 0,239 | 0,49 | 0,345 |
| Q4G0A6 | Probable ubiquitin carboxyl-terminal hydrolase FAM188B | FAM188B | 1 | 1 | 84,3 | -0,06 | 0,949 | 0,18 | 0,465 |
| Q02809 | Procollagen-lysine,2-oxoglutarate 5-dioxygenase 1 | PLOD1 | 2 | 2 | 83,5 | 0,56 | 0,191 | -0,34 | 0,953 |
| P07737 | Profilin-1 | PFN1 | 1 | 1 | 15,0 | -0,11 | 0,512 | 0,03 | 0,686 |
| P35080 | Profilin-2 | PFN2 | 1 | 1 | 15,0 | 0,14 | 0,434 | -0,50 | 0,274 |
| Q8WUM4 | Programmed cell death 6-interacting protein | PDCD6IP | 18 | 18 | 96,0 | 0,59 | 0,030 | 0,37 | 0,110 |
| Q9BUL8 | Programmed cell death protein 10 | PDCD10 | 4 | 4 | 24,7 | 0,05 | 0,751 | -0,08 | 0,788 |
| Q14005 | Pro-interleukin-16 | IL16 | 1 | 1 | 141,7 | -0,60 | 0,327 | -0,33 | 0,627 |
| Q9UQ80 | Proliferation-associated protein 2G4 | PA2G4 | 1 | 1 | 43,8 | 0,19 | 0,335 | 1,75 | 0,082 |
| Q86WR7 | Proline and serine-rich protein 2 | PROSER2 | 1 | 1 | 45,8 | -0,06 | 0,964 | -0,10 | 0,865 |
| O94903 | Proline synthase co-transcribed bacterial homolog protein | PROSC | 1 | 1 | 30,3 | -0,06 | 0,697 | 0,10 | 0,514 |
| Q5FWE3 | Proline-rich transmembrane protein 3 | PRRT3 | 2 | 2 | 102,1 | -1,21 | 0,257 | -1,49 | 0,304 |
| P48147 | Prolyl endopeptidase | PREP | 1 | 1 | 80,6 | -0,20 | 0,500 | 0,09 | 0,698 |
| O43490 | Prominin-1 | PROM1 | 11 | 11 | 97,1 | 0,49 | 0,003 | -0,92 | 0,004 |
| Q8N271 | Prominin-2 | PROM2 | 7 | 7 | 91,8 | -0,92 | 0,126 | -0,62 | 0,413 |
| Q9P2B2 | Prostaglandin F2 receptor negative regulator | PTGFRN | 5 | 5 | 98,5 | -0,13 | 0,873 | 0,03 | 0,591 |
| Q14914 | Prostaglandin reductase 1 | PTGR1 | 2 | 2 | 35,8 | -0,07 | 0,814 | -0,29 | 0,097 |
| Q16651 | Prostasin | PRSS8 | 1 | 1 | 36,4 | -0,07 | 0,431 | -0,21 | 0,312 |
| Q06323 | Proteasome activator complex subunit 1 | PSME1 | 1 | 1 | 28,7 | -0,15 | 0,757 | -0,03 | 0,998 |
| P25788 | Proteasome subunit alpha type-3 | PSMA3 | 1 | 1 | 28,4 | 0,28 | 0,501 | -0,24 | 0,497 |
| P25789 | Proteasome subunit alpha type-4 | PSMA4 | 1 | 1 | 29,5 | -0,15 | 0,799 | -0,02 | 0,924 |
| P28066 | Proteasome subunit alpha type-5 | PSMA5 | 2 | 2 | 26,4 | 0,19 | 0,699 | -0,08 | 0,888 |
| P20618 | Proteasome subunit beta type-1 | PSMB1 | 1 | 1 | 26,5 | 0,54 | 0,313 | 0,43 | 0,437 |
| P28070 | Proteasome subunit beta type-4 | PSMB4 | 1 | 1 | 29,2 | 0,00 | 0,950 | -0,47 | 0,289 |
| P28072 | Proteasome subunit beta type-6 | PSMB6 | 1 | 1 | 25,3 | -0,03 | 0,806 | -0,08 | 0,950 |
| Q96IU4 | Protein ABHD14B | ABHD14B | 1 | 1 | 22,3 | -0,09 | 0,428 | -0,06 | 0,793 |
| Q99873 | Protein arginine N-methyltransferase 1 | PRMT1 | 1 | 1 | 41,5 | 0,60 | 0,105 | 0,21 | 0,401 |
| Q96SW2 | Protein cereblon | CRBN | 1 | 1 | 50,5 | -0,87 | 0,247 | -0,49 | 0,670 |
| Q9Y3M2 | Protein chibby homolog 1 | CBY1 | 1 | 1 | 14,5 | -0,54 | 0,488 | 0,45 | 0,557 |
| Q9BUF7 | Protein crumbs homolog 3 | CRB3 | 1 | 1 | 12,8 | 0,02 | 0,848 | -0,51 | 0,423 |
| Q99497 | Protein deglycase DJ-1 | PARK7 | 2 | 2 | 19,9 | -0,07 | 0,726 | 0,04 | 0,478 |
| O60610 | Protein diaphanous homolog 1 | DIAPH1 | 2 | 2 | 141,3 | -0,17 | 0,989 | -0,11 | 0,891 |
| P07237 | Protein disulfide-isomerase | P4HB | 4 | 4 | 57,1 | 0,36 | 0,341 | 0,25 | 0,206 |
| Q9Y3R5 | Protein dopey-2 | DOPEY2 | 6 | 6 | 258,1 | -0,05 | 0,830 | -0,32 | 0,117 |
| Q9BVM2 | Protein DPCD | DPCD | 2 | 2 | 23,2 | 0,09 | 0,537 | 0,04 | 0,647 |
| Q14156 | Protein EFR3 homolog A | EFR3A | 1 | 1 | 92,9 | 0,79 | 0,337 | 0,61 | 0,163 |
| Q8N8S7 | Protein enabled homolog | ENAH | 2 | 2 | 66,5 | -0,26 | 0,918 | -0,28 | 0,238 |
| Q9NUQ9 | Protein FAM49B | FAM49B | 1 | 1 | 36,7 | 0,07 | 0,291 | -0,20 | 0,192 |
| Q6ZS17 | Protein FAM65A | FAM65A | 3 | 3 | 132,2 | -0,56 | 0,089 | -0,24 | 0,364 |
| Q6ZTR7 | Protein FAM92B | FAM92B | 3 | 3 | 34,8 | -0,78 | 0,133 | -0,25 | 0,784 |
| O94915 | Protein furry homolog-like | FRYL | 1 | 1 | 339,4 | -0,19 | 0,678 | 0,39 | 0,218 |
| O15037 | Protein KHNYN | KHNYN | 1 | 1 | 74,5 | 0,04 | 0,797 | -0,01 | 0,975 |
| Q9UNF0 | Protein kinase C and casein kinase substrate in neurons protein 2 | PACSIN2 | 7 | 7 | 55,7 | -0,36 | 0,146 | -0,42 | 0,054 |
| Q9UKS6 | Protein kinase C and casein kinase substrate in neurons protein 3 | PACSIN3 | 6 | 6 | 48,5 | -0,50 | 0,043 | 0,00 | 0,853 |
| Q05655 | Protein kinase C delta type | PRKCD | 3 | 3 | 77,5 | 1,05 | 0,043 | 0,35 | 0,289 |
| Q969X1 | Protein lifeguard 3 | TMBIM1 | 1 | 1 | 34,6 | 0,63 | 0,231 | 0,33 | 0,769 |
| Q9NUP9 | Protein lin-7 homolog C | LIN7C | 2 | 2 | 21,8 | 0,05 | 0,214 | 0,09 | 0,851 |
| Q9BZQ8 | Protein Niban | FAM129A | 5 | 5 | 103,1 | 0,03 | 0,613 | 0,34 | 0,244 |
| Q8WVF1 | Protein OSCP1 | OSCP1 | 1 | 1 | 44,6 | 0,27 | 0,249 | -0,22 | 0,357 |
| Q8TAE6 | Protein phosphatase 1 regulatory subunit 14C | PPP1R14C | 1 | 1 | 17,8 | -0,03 | 0,683 | -1,27 | 0,281 |
| Q96I34 | Protein phosphatase 1 regulatory subunit 16A | PPP1R16A | 4 | 4 | 57,8 | -0,25 | 0,652 | -0,08 | 0,731 |
| Q6ZMI0 | Protein phosphatase 1 regulatory subunit 21 | PPP1R21 | 1 | 1 | 88,3 | -0,34 | 0,395 | -0,11 | 0,693 |
| Q5R3F8 | Protein phosphatase 1 regulatory subunit 29 | ELFN2 | 1 | 1 | 89,6 | -1,99 | 0,158 | -1,63 | 0,350 |
| P35813 | Protein phosphatase 1A | PPM1A | 1 | 1 | 42,4 | -0,79 | 0,380 | -0,35 | 0,867 |
| Q9ULR3 | Protein phosphatase 1H | PPM1H | 2 | 2 | 56,4 | -0,58 | 0,223 | -0,69 | 0,029 |
| Q8WVV4 | Protein POF1B | POF1B | 4 | 4 | 68,0 | 1,47 | 0,453 | 0,96 | 0,425 |
| P31949 | Protein S100-A11 | S100A11 | 2 | 2 | 11,7 | 0,23 | 0,132 | -0,12 | 0,731 |
| Q99584 | Protein S100-A13 | S100A13 | 1 | 1 | 11,5 | -0,27 | 0,581 | -0,33 | 0,484 |
| Q9HCY8 | Protein S100-A14 | S100A14 | 2 | 2 | 11,7 | -0,38 | 0,502 | -0,62 | 0,406 |
| Q96FQ6 | Protein S100-A16 | S100A16 | 2 | 2 | 11,8 | -0,25 | 0,540 | -0,18 | 0,238 |
| P29034 | Protein S100-A2 | S100A2 | 1 | 1 | 11,1 | 0,14 | 0,545 | -0,39 | 0,627 |
| P06703 | Protein S100-A6 | S100A6 | 3 | 3 | 10,2 | 0,10 | 0,161 | -0,04 | 0,760 |
| P31151 | Protein S100-A7 | S100A7 | 1 | 1 | 11,5 | 0,55 | 0,807 | -1,10 | 0,011 |
| P05109 | Protein S100-A8 | S100A8 | 1 | 1 | 10,8 | 0,21 | 0,409 | -0,55 | 0,361 |
| P25815 | Protein S100-P | S100P | 1 | 1 | 10,4 | -0,13 | 0,726 | 0,04 | 0,959 |
| Q92734 | Protein TFG | TFG | 2 | 2 | 43,4 | 0,43 | 0,070 | -0,34 | 0,418 |
| Q9C0H2 | Protein tweety homolog 3 | TTYH3 | 1 | 1 | 57,5 | -0,52 | 0,082 | -0,41 | 0,035 |
| O14795 | Protein unc-13 homolog B | UNC13B | 7 | 7 | 180,6 | -1,04 | 0,132 | -0,67 | 0,373 |
| Q9H3U1 | Protein unc-45 homolog A | UNC45A | 1 | 1 | 103,0 | -1,07 | 0,852 | -0,50 | 0,717 |
| Q76NI1 | Protein very KIND | KNDC1 | 1 | 1 | 191,3 | -0,61 | 0,195 | -0,46 | 0,279 |
| O75695 | Protein XRP2 | RP2 | 1 | 1 | 39,6 | -0,13 | 0,467 | -0,35 | 0,173 |
| P21980 | Protein-glutamine gamma-glutamyltransferase 2 | TGM2 | 6 | 6 | 77,3 | -0,32 | 0,151 | 0,09 | 0,544 |
| O43548 | Protein-glutamine gamma-glutamyltransferase 5 | TGM5 | 1 | 1 | 80,7 | 1,04 | 0,534 | 0,59 | 0,570 |
| Q08188 | Protein-glutamine gamma-glutamyltransferase E | TGM3 | 5 | 5 | 76,6 | 0,53 | 0,806 | 0,33 | 0,637 |
| P22735 | Protein-glutamine gamma-glutamyltransferase K | TGM1 | 4 | 4 | 89,7 | 0,29 | 0,942 | 0,32 | 0,747 |
| P22061 | Protein-L-isoaspartate(D-aspartate) O-methyltransferase | PCMT1 | 1 | 1 | 24,6 | -0,02 | 0,949 | 0,13 | 0,331 |
| Q04941 | Proteolipid protein 2 | PLP2 | 1 | 1 | 16,7 | -0,11 | 0,914 | -0,53 | 0,140 |
| Q9NYQ8 | Protocadherin Fat 2 | FAT2 | 4 | 4 | 479,0 | -0,44 | 0,697 | -0,17 | 0,983 |
| Q08174 | Protocadherin-1 | PCDH1 | 1 | 1 | 114,7 | -1,01 | 0,999 | -0,96 | 0,978 |
| P12931 | Proto-oncogene tyrosine-protein kinase Src | SRC | 3 | 6 | 59,8 | -0,07 | 0,693 | 0,10 | 0,351 |
| P00491 | Purine nucleoside phosphorylase | PNP | 1 | 1 | 32,1 | 0,54 | 0,065 | -0,37 | 0,611 |
| P55786 | Puromycin-sensitive aminopeptidase | NPEPPS | 5 | 5 | 103,2 | 0,07 | 0,340 | -0,06 | 0,667 |
| Q6NVV1 | Putative 60S ribosomal protein L13a protein RPL13AP3 | RPL13AP3 | 1 | 1 | 12,1 | -0,58 | 0,582 | 0,84 | 0,219 |
| A6NGU5 | Putative gamma-glutamyltranspeptidase 3 | GGT3P | 4 | 4 | 61,5 | 0,03 | 0,877 | -0,56 | 0,064 |
| Q6GMV3 | Putative peptidyl-tRNA hydrolase PTRHD1 | PTRHD1 | 1 | 1 | 15,8 | -0,57 | 0,079 | -0,20 | 0,588 |
| Q13670 | Putative postmeiotic segregation increased 2-like protein 11 | PMS2P11 | 1 | 1 | 28,5 | 0,20 | 0,225 | 0,10 | 0,543 |
| Q8IZP2 | Putative protein FAM10A4 | ST13P4 | 2 | 2 | 27,4 | 0,09 | 0,397 | -0,16 | 0,206 |
| P14618 | Pyruvate kinase PKM | PKM | 8 | 8 | 57,9 | 0,13 | 0,242 | 0,41 | 0,012 |
| P31150 | Rab GDP dissociation inhibitor alpha | GDI1 | 1 | 3 | 50,6 | -0,58 | 0,311 | 0,14 | 0,811 |
| P50395 | Rab GDP dissociation inhibitor beta | GDI2 | 2 | 4 | 50,6 | -0,56 | 0,230 | -0,53 | 0,243 |
| Q9H5N1 | Rab GTPase-binding effector protein 2 | RABEP2 | 1 | 1 | 63,5 | -0,82 | 0,069 | -1,26 | 0,024 |
| Q6WKZ4 | Rab11 family-interacting protein 1 | RAB11FIP1 | 3 | 3 | 137,1 | -0,42 | 0,467 | -0,27 | 0,729 |
| Q9P2R3 | Rabankyrin-5 | ANKFY1 | 2 | 2 | 128,3 | 1,11 | 0,068 | 0,59 | 0,144 |
| P31751 | RAC-beta serine/threonine-protein kinase | AKT2 | 1 | 1 | 55,7 | 1,07 | 0,015 | -0,27 | 0,463 |
| Q5TD94 | Radial spoke head protein 4 homolog A | RSPH4A | 5 | 5 | 80,7 | -0,46 | 0,107 | -0,27 | 0,281 |
| Q9H1X1 | Radial spoke head protein 9 homolog | RSPH9 | 2 | 2 | 31,3 | -0,19 | 0,495 | -0,25 | 0,494 |
| P35241 | Radixin | RDX | 10 | 14 | 68,5 | -0,38 | 0,091 | 0,07 | 0,957 |
| Q2PPJ7 | Ral GTPase-activating protein subunit alpha-2 | RALGAPA2 | 3 | 3 | 210,6 | -0,04 | 0,669 | -0,28 | 0,929 |
| P46940 | Ras GTPase-activating-like protein IQGAP1 | IQGAP1 | 14 | 14 | 189,1 | 0,01 | 0,777 | 0,47 | 0,007 |
| Q15404 | Ras suppressor protein 1 | RSU1 | 1 | 1 | 31,5 | 0,03 | 0,652 | 0,08 | 0,604 |
| P15153 | Ras-related C3 botulinum toxin substrate 2 | RAC2 | 1 | 1 | 21,4 | -0,01 | 0,983 | -0,07 | 0,748 |
| P61026 | Ras-related protein Rab-10 | RAB10 | 4 | 5 | 22,5 | -0,07 | 0,806 | -0,23 | 0,292 |
| P62491 | Ras-related protein Rab-11A | RAB11A | 5 | 6 | 24,4 | 0,15 | 0,157 | -0,08 | 0,729 |
| P51153 | Ras-related protein Rab-13 | RAB13 | 3 | 4 | 22,8 | -0,32 | 0,138 | 0,01 | 0,967 |
| P61106 | Ras-related protein Rab-14 | RAB14 | 3 | 4 | 23,9 | 0,27 | 0,230 | -0,02 | 0,373 |
| Q9NP72 | Ras-related protein Rab-18 | RAB18 | 2 | 2 | 23,0 | 0,24 | 0,297 | 0,20 | 0,467 |
| P62820 | Ras-related protein Rab-1A | RAB1A | 2 | 6 | 22,7 | -0,06 | 0,090 | -0,13 | 0,503 |
| Q9H0U4 | Ras-related protein Rab-1B | RAB1B | 1 | 5 | 22,2 | 0,11 | 0,279 | 0,38 | 0,567 |
| Q9NX57 | Ras-related protein Rab-20 | RAB20 | 1 | 1 | 26,3 | -0,10 | 0,940 | -0,70 | 0,411 |
| Q9UL25 | Ras-related protein Rab-21 | RAB21 | 1 | 1 | 24,3 | 0,28 | 0,305 | 0,15 | 0,757 |
| Q9ULC3 | Ras-related protein Rab-23 | RAB23 | 2 | 2 | 26,6 | -0,08 | 0,567 | -0,21 | 0,022 |
| P57735 | Ras-related protein Rab-25 | RAB25 | 5 | 6 | 23,5 | 0,19 | 0,424 | -0,38 | 0,298 |
| P51159 | Ras-related protein Rab-27A | RAB27A | 1 | 1 | 24,9 | 0,04 | 0,681 | -0,78 | 0,137 |
| O00194 | Ras-related protein Rab-27B | RAB27B | 2 | 2 | 24,6 | -0,22 | 0,502 | -0,48 | 0,091 |
| P61019 | Ras-related protein Rab-2A | RAB2A | 1 | 1 | 23,5 | -0,04 | 0,915 | -0,02 | 0,844 |
| Q9BZG1 | Ras-related protein Rab-34 | RAB34 | 2 | 2 | 29,0 | 0,14 | 0,538 | -0,72 | 0,259 |
| Q15286 | Ras-related protein Rab-35 | RAB35 | 3 | 4 | 23,0 | -0,18 | 0,291 | -0,26 | 0,253 |
| O95716 | Ras-related protein Rab-3D | RAB3D | 1 | 4 | 24,3 | -1,02 | 0,175 | -0,83 | 0,231 |
| P20338 | Ras-related protein Rab-4A | RAB4A | 1 | 2 | 24,4 | 0,33 | 0,116 | 0,22 | 0,053 |
| P20339 | Ras-related protein Rab-5A | RAB5A | 1 | 2 | 23,6 | -0,35 | 0,654 | -0,85 | 0,104 |
| P61020 | Ras-related protein Rab-5B | RAB5B | 1 | 2 | 23,7 | 0,12 | 0,139 | -0,12 | 0,676 |
| P51148 | Ras-related protein Rab-5C | RAB5C | 3 | 4 | 23,5 | 0,29 | 0,073 | -0,10 | 0,623 |
| P20340 | Ras-related protein Rab-6A | RAB6A | 2 | 3 | 23,6 | 0,12 | 0,343 | -0,44 | 0,245 |
| P51149 | Ras-related protein Rab-7a | RAB7A | 3 | 3 | 23,5 | 0,70 | 0,179 | -0,04 | 0,973 |
| P61006 | Ras-related protein Rab-8A | RAB8A | 1 | 4 | 23,7 | 0,05 | 0,709 | -0,17 | 0,917 |
| P11233 | Ras-related protein Ral-A | RALA | 2 | 2 | 23,6 | -0,24 | 0,257 | 0,13 | 0,386 |
| P11234 | Ras-related protein Ral-B | RALB | 1 | 1 | 23,4 | -0,22 | 0,475 | -0,26 | 0,051 |
| A6NIZ1 | Ras-related protein Rap-1b-like protein |  | 3 | 4 | 20,9 | -0,18 | 0,086 | -0,08 | 0,353 |
| P61225 | Ras-related protein Rap-2b | RAP2B | 1 | 1 | 20,5 | -0,19 | 0,720 | 0,00 | 0,979 |
| P10301 | Ras-related protein R-Ras | RRAS | 3 | 4 | 23,5 | -0,16 | 0,641 | -0,08 | 0,920 |
| P62070 | Ras-related protein R-Ras2 | RRAS2 | 4 | 5 | 23,4 | -0,12 | 0,676 | -0,02 | 0,955 |
| P63244 | Receptor of activated protein C kinase 1 | RACK1 | 3 | 3 | 35,1 | 0,69 | 0,348 | 0,29 | 0,291 |
| Q12913 | Receptor-type tyrosine-protein phosphatase eta | PTPRJ | 6 | 6 | 145,9 | 0,19 | 0,201 | -0,28 | 0,189 |
| Q9HD43 | Receptor-type tyrosine-protein phosphatase H | PTPRH | 1 | 1 | 122,3 | 0,80 | 0,006 | 0,74 | 0,393 |
| Q9UGC6 | Regulator of G-protein signaling 17 | RGS17 | 1 | 1 | 24,3 | 0,16 | 0,244 | -0,23 | 0,181 |
| P49795 | Regulator of G-protein signaling 19 | RGS19 | 1 | 1 | 24,6 | -0,24 | 0,823 | -0,19 | 0,832 |
| O75787 | Renin receptor | ATP6AP2 | 1 | 1 | 39,0 | 0,63 | 0,301 | 0,32 | 0,442 |
| P00352 | Retinal dehydrogenase 1 | ALDH1A1 | 3 | 4 | 54,8 | 0,16 | 0,175 | 0,30 | 0,122 |
| Q5VY80 | Retinoic acid early transcript 1L protein | RAET1L | 1 | 1 | 27,5 | 0,32 | 0,290 | 1,02 | 0,061 |
| P49788 | Retinoic acid receptor responder protein 1 | RARRES1 | 3 | 3 | 33,3 | -0,01 | 0,422 | -0,44 | 0,792 |
| Q8NFJ5 | Retinoic acid-induced protein 3 | GPRC5A | 2 | 2 | 40,2 | -0,84 | 0,356 | -0,06 | 0,986 |
| P52565 | Rho GDP-dissociation inhibitor 1 | ARHGDIA | 1 | 1 | 23,2 | -0,18 | 0,212 | -0,21 | 0,316 |
| P52566 | Rho GDP-dissociation inhibitor 2 | ARHGDIB | 1 | 1 | 23,0 | -0,23 | 0,221 | -0,23 | 0,224 |
| Q07960 | Rho GTPase-activating protein 1 | ARHGAP1 | 1 | 1 | 50,4 | 0,11 | 0,655 | -0,15 | 0,677 |
| Q8IWW6 | Rho GTPase-activating protein 12 | ARHGAP12 | 1 | 1 | 96,2 | 0,41 | 0,106 | -0,23 | 0,785 |
| Q68EM7 | Rho GTPase-activating protein 17 | ARHGAP17 | 2 | 2 | 95,4 | -0,26 | 0,333 | -0,27 | 0,148 |
| Q8N392 | Rho GTPase-activating protein 18 | ARHGAP18 | 8 | 8 | 74,9 | -0,11 | 0,943 | -0,05 | 0,798 |
| Q6ZUM4 | Rho GTPase-activating protein 27 | ARHGAP27 | 3 | 3 | 98,3 | -0,60 | 0,023 | -0,14 | 0,697 |
| Q9C0H5 | Rho GTPase-activating protein 39 | ARHGAP39 | 6 | 6 | 121,2 | 0,13 | 0,509 | -0,11 | 0,389 |
| Q5VV41 | Rho guanine nucleotide exchange factor 16 | ARHGEF16 | 1 | 1 | 80,1 | -0,66 | 0,305 | -0,74 | 0,037 |
| A1IGU5 | Rho guanine nucleotide exchange factor 37 | ARHGEF37 | 1 | 1 | 76,2 | 0,00 | 0,788 | -0,59 | 0,446 |
| Q13464 | Rho-associated protein kinase 1 | ROCK1 | 1 | 1 | 158,1 | -0,09 | 0,668 | -0,02 | 0,850 |
| O75116 | Rho-associated protein kinase 2 | ROCK2 | 2 | 2 | 160,8 | -0,67 | 0,339 | 0,07 | 0,842 |
| Q8TCX5 | Rhophilin-1 | RHPN1 | 1 | 1 | 76,2 | -0,49 | 0,272 | -0,42 | 0,080 |
| Q8IUC4 | Rhophilin-2 | RHPN2 | 5 | 5 | 76,9 | -0,17 | 0,525 | -0,42 | 0,051 |
| P62745 | Rho-related GTP-binding protein RhoB | RHOB | 1 | 1 | 22,1 | -0,30 | 0,117 | -0,53 | 0,100 |
| P08134 | Rho-related GTP-binding protein RhoC | RHOC | 1 | 1 | 22,0 | -0,16 | 0,161 | -0,19 | 0,331 |
| P84095 | Rho-related GTP-binding protein RhoG | RHOG | 4 | 4 | 21,3 | -0,21 | 0,421 | -0,19 | 0,243 |
| Q8N443 | RIB43A-like with coiled-coils protein 1 | RIBC1 | 1 | 1 | 44,0 | 0,03 | 0,633 | 0,71 | 0,355 |
| P13489 | Ribonuclease inhibitor | RNH1 | 1 | 1 | 49,9 | -0,47 | 0,542 | 0,14 | 0,586 |
| Q9P2E9 | Ribosome-binding protein 1 | RRBP1 | 2 | 2 | 152,4 | -0,65 | 0,696 | 0,71 | 0,157 |
| Q96AT9 | Ribulose-phosphate 3-epimerase | RPE | 1 | 1 | 24,9 | 0,12 | 0,500 | -0,28 | 0,686 |
| Q969X0 | RILP-like protein 2 | RILPL2 | 2 | 2 | 24,0 | -0,16 | 0,936 | -0,53 | 0,009 |
| Q96C74 | Ropporin-1-like protein | ROPN1L | 3 | 3 | 26,1 | -0,09 | 0,880 | 0,09 | 0,415 |
| Q9Y265 | RuvB-like 1 | RUVBL1 | 3 | 3 | 50,2 | 0,43 | 0,097 | -0,38 | 0,335 |
| Q9Y230 | RuvB-like 2 | RUVBL2 | 5 | 5 | 51,1 | 0,40 | 0,023 | -0,32 | 0,083 |
| O95171 | Sciellin | SCEL | 2 | 2 | 77,5 | -0,82 | 0,020 | 0,09 | 0,846 |
| Q86VW0 | SEC14 domain and spectrin repeat-containing protein 1 | SESTD1 | 2 | 2 | 79,3 | -0,01 | 0,915 | 0,31 | 0,175 |
| Q12765 | Secernin-1 | SCRN1 | 1 | 1 | 46,4 | 0,16 | 0,493 | -0,32 | 0,342 |
| O15126 | Secretory carrier-associated membrane protein 1 | SCAMP1 | 1 | 1 | 37,9 | 0,24 | 0,153 | -0,14 | 0,538 |
| Q13228 | Selenium-binding protein 1 | SELENBP1 | 4 | 4 | 52,4 | 0,02 | 0,526 | 0,18 | 0,378 |
| Q13275 | Semaphorin-3F | SEMA3F | 1 | 1 | 88,3 | -0,83 | 0,507 | -0,39 | 0,960 |
| P04279 | Semenogelin-1 | SEMG1 | 1 | 1 | 52,1 | 1,49 | 0,762 | 0,83 | 0,369 |
| Q15019 | Septin-2 | SEP2 | 2 | 2 | 41,5 | 0,43 | 0,163 | 1,03 | 0,069 |
| Q16181 | Septin-7 | SEP7 | 2 | 2 | 50,6 | 0,71 | 0,289 | 1,01 | 0,133 |
| Q9UHD8 | Septin-9 | SEP9 | 7 | 7 | 65,4 | 0,17 | 0,711 | 0,48 | 0,099 |
| Q9NRX5 | Serine incorporator 1 | SERINC1 | 1 | 1 | 50,5 | 0,94 | 0,623 | -0,01 | 0,867 |
| Q13530 | Serine incorporator 3 | SERINC3 | 1 | 1 | 52,5 | 0,55 | 0,105 | -0,03 | 0,965 |
| Q86VE9 | Serine incorporator 5 | SERINC5 | 3 | 3 | 47,0 | -0,23 | 0,613 | 0,22 | 0,093 |
| Q9GZT4 | Serine racemase | SRR | 1 | 1 | 36,5 | -0,38 | 0,245 | -0,44 | 0,017 |
| O94804 | Serine/threonine-protein kinase 10 | STK10 | 6 | 6 | 112,1 | -0,21 | 0,601 | 0,21 | 0,424 |
| Q9Y6E0 | Serine/threonine-protein kinase 24 | STK24 | 5 | 5 | 49,3 | 0,22 | 0,311 | 0,05 | 0,704 |
| O00506 | Serine/threonine-protein kinase 25 | STK25 | 2 | 3 | 48,1 | 0,19 | 0,332 | 0,05 | 0,677 |
| Q9P289 | Serine/threonine-protein kinase 26 | STK26 | 3 | 4 | 46,5 | 0,43 | 0,331 | -0,16 | 0,997 |
| Q15208 | Serine/threonine-protein kinase 38 | STK38 | 1 | 1 | 54,2 | -0,15 | 0,133 | 0,15 | 0,318 |
| Q8IWU2 | Serine/threonine-protein kinase LMTK2 | LMTK2 | 2 | 2 | 164,8 | -0,34 | 0,160 | -0,58 | 0,144 |
| P42345 | Serine/threonine-protein kinase mTOR | MTOR | 1 | 1 | 288,7 | 0,10 | 0,575 | 0,25 | 0,878 |
| O95747 | Serine/threonine-protein kinase OSR1 | OXSR1 | 1 | 1 | 58,0 | -0,20 | 0,089 | -0,19 | 0,595 |
| Q13153 | Serine/threonine-protein kinase PAK 1 | PAK1 | 2 | 2 | 60,6 | 0,76 | 0,057 | -0,03 | 0,975 |
| O96013 | Serine/threonine-protein kinase PAK 4 | PAK4 | 3 | 3 | 64,0 | -0,25 | 0,236 | 0,00 | 0,943 |
| Q7L7X3 | Serine/threonine-protein kinase TAO1 | TAOK1 | 3 | 3 | 116,0 | -0,34 | 0,416 | 0,08 | 0,940 |
| P63151 | Serine/threonine-protein phosphatase 2A 55 kDa regulatory subunit B alpha isoform | PPP2R2A | 2 | 2 | 51,7 | -0,17 | 0,558 | 0,10 | 0,494 |
| Q15172 | Serine/threonine-protein phosphatase 2A 56 kDa regulatory subunit alpha isoform | PPP2R5A | 1 | 1 | 56,2 | -0,26 | 0,794 | 0,19 | 0,715 |
| P30153 | Serine/threonine-protein phosphatase 2A 65 kDa regulatory subunit A alpha isoform | PPP2R1A | 4 | 4 | 65,3 | 0,20 | 0,326 | 0,07 | 0,848 |
| Q15257 | Serine/threonine-protein phosphatase 2A activator | PPP2R4 | 3 | 3 | 40,6 | 0,25 | 0,416 | -0,03 | 0,980 |
| P62714 | Serine/threonine-protein phosphatase 2A catalytic subunit beta isoform | PPP2CB | 1 | 1 | 35,6 | 0,15 | 0,244 | 0,31 | 0,252 |
| P48454 | Serine/threonine-protein phosphatase 2B catalytic subunit gamma isoform | PPP3CC | 1 | 1 | 58,1 | -1,76 | 0,584 | -1,10 | 0,900 |
| P62140 | Serine/threonine-protein phosphatase PP1-beta catalytic subunit | PPP1CB | 1 | 1 | 37,2 | 0,05 | 0,770 | -0,34 | 0,003 |
| P36873 | Serine/threonine-protein phosphatase PP1-gamma catalytic subunit | PPP1CC | 1 | 1 | 37,0 | -0,37 | 0,033 | -0,08 | 0,650 |
| P49591 | Serine--tRNA ligase, cytoplasmic | SARS | 2 | 2 | 58,7 | -0,05 | 0,986 | 0,07 | 0,597 |
| Q96P63 | Serpin B12 | SERPINB12 | 2 | 2 | 46,2 | 0,96 | 0,734 | 0,35 | 0,755 |
| P29508 | Serpin B3 | SERPINB3 | 4 | 7 | 44,5 | -0,33 | 0,262 | 0,30 | 0,279 |
| P48594 | Serpin B4 | SERPINB4 | 1 | 4 | 44,8 | 0,39 | 0,183 | 0,69 | 0,211 |
| P36952 | Serpin B5 | SERPINB5 | 1 | 1 | 42,1 | -0,31 | 0,790 | -0,52 | 0,453 |
| P50454 | Serpin H1 | SERPINH1 | 1 | 1 | 46,4 | -1,11 | 0,027 | 0,45 | 0,318 |
| P02768 | Serum albumin | ALB | 7 | 7 | 69,3 | 1,93 | 0,502 | 0,03 | 0,856 |
| P0DJI8 | Serum amyloid A-1 protein | SAA1 | 2 | 2 | 13,5 | 2,43 | 0,042 | -0,32 | 0,687 |
| Q9H788 | SH2 domain-containing protein 4A | SH2D4A | 1 | 1 | 52,7 | 0,72 | 0,210 | -0,02 | 0,733 |
| Q9UPX8 | SH3 and multiple ankyrin repeat domains protein 2 | SHANK2 | 7 | 7 | 158,7 | -0,69 | 0,184 | -0,66 | 0,071 |
| A1X283 | SH3 and PX domain-containing protein 2B | SH3PXD2B | 2 | 2 | 101,5 | 0,10 | 0,283 | 0,41 | 0,230 |
| Q9H299 | SH3 domain-binding glutamic acid-rich-like protein 3 | SH3BGRL3 | 2 | 2 | 10,4 | -0,31 | 0,359 | -0,23 | 0,923 |
| Q9P0V3 | SH3 domain-binding protein 4 | SH3BP4 | 3 | 3 | 107,4 | -0,53 | 0,008 | -0,18 | 0,338 |
| P29353 | SHC-transforming protein 1 | SHC1 | 3 | 3 | 62,8 | 0,55 | 0,136 | 0,09 | 0,089 |
| Q9NR45 | Sialic acid synthase | NANS | 1 | 1 | 40,3 | 0,14 | 0,242 | 0,31 | 0,376 |
| Q9UQ49 | Sialidase-3 | NEU3 | 2 | 2 | 48,2 | -0,55 | 0,472 | -0,08 | 0,916 |
| Q9NRA2 | Sialin | SLC17A5 | 1 | 1 | 54,6 | 0,06 | 0,706 | 0,31 | 0,222 |
| P49458 | Signal recognition particle 9 kDa protein | SRP9 | 1 | 1 | 10,1 | 0,14 | 0,304 | 0,51 | 0,318 |
| P40763 | Signal transducer and activator of transcription 3 | STAT3 | 1 | 1 | 88,0 | 0,03 | 0,845 | 0,16 | 0,477 |
| Q7Z6B7 | SLIT-ROBO Rho GTPase-activating protein 1 | SRGAP1 | 1 | 2 | 124,2 | 0,48 | 0,168 | 0,08 | 0,914 |
| O75044 | SLIT-ROBO Rho GTPase-activating protein 2 | SRGAP2 | 3 | 4 | 120,8 | -0,67 | 0,398 | -0,51 | 0,422 |
| O43295 | SLIT-ROBO Rho GTPase-activating protein 3 | SRGAP3 | 1 | 1 | 124,4 | 0,63 | 0,784 | 0,28 | 0,371 |
| K7EJ46 | Small integral membrane protein 22 | SMIM22 | 1 | 1 | 14,6 | -0,27 | 0,119 | -0,47 | 0,062 |
| Q71RC9 | Small integral membrane protein 5 | SMIM5 | 1 | 1 | 8,5 | -0,21 | 0,091 | 0,14 | 0,674 |
| Q8NHG7 | Small VCP/p97-interacting protein | SVIP | 3 | 3 | 8,4 | -0,38 | 0,018 | -0,31 | 0,008 |
| Q9UN76 | Sodium- and chloride-dependent neutral and basic amino acid transporter B(0+) | SLC6A14 | 5 | 5 | 72,1 | 0,24 | 0,385 | 1,48 | 0,045 |
| Q9NP91 | Sodium- and chloride-dependent transporter XTRP3 | SLC6A20 | 1 | 1 | 65,9 | 1,91 | 0,073 | -0,86 | 0,518 |
| P13866 | Sodium/glucose cotransporter 1 | SLC5A1 | 2 | 2 | 73,4 | 3,68 | 0,013 | -0,18 | 0,638 |
| O43868 | Sodium/nucleoside cotransporter 2 | SLC28A2 | 1 | 1 | 71,9 | 0,43 | 0,085 | -0,25 | 0,927 |
| P05023 | Sodium/potassium-transporting ATPase subunit alpha-1 | ATP1A1 | 9 | 11 | 112,8 | 0,02 | 0,839 | 0,27 | 0,123 |
| P05026 | Sodium/potassium-transporting ATPase subunit beta-1 | ATP1B1 | 3 | 3 | 35,0 | 1,29 | 0,026 | 1,47 | 0,056 |
| O95436 | Sodium-dependent phosphate transport protein 2B | SLC34A2 | 5 | 5 | 75,7 | 0,22 | 0,472 | -0,59 | 0,044 |
| P55011 | Solute carrier family 12 member 2 | SLC12A2 | 3 | 3 | 131,4 | 0,28 | 0,501 | 0,47 | 0,024 |
| Q13183 | Solute carrier family 13 member 2 | SLC13A2 | 1 | 1 | 64,4 | 1,09 | 0,064 | -0,64 | 0,499 |
| Q16348 | Solute carrier family 15 member 2 | SLC15A2 | 4 | 4 | 81,7 | -0,72 | 0,112 | -0,66 | 0,032 |
| Q9H015 | Solute carrier family 22 member 4 | SLC22A4 | 2 | 2 | 62,1 | 0,24 | 0,645 | -0,21 | 0,551 |
| O76082 | Solute carrier family 22 member 5 | SLC22A5 | 1 | 1 | 62,7 | 0,14 | 0,708 | -0,13 | 0,968 |
| Q9UHI7 | Solute carrier family 23 member 1 | SLC23A1 | 1 | 1 | 64,8 | -1,13 | 0,385 | -0,37 | 0,586 |
| Q9NQ40 | Solute carrier family 52, riboflavin transporter, member 3 | SLC52A3 | 1 | 1 | 50,8 | -0,89 | 0,165 | 0,35 | 0,749 |
| O94875 | Sorbin and SH3 domain-containing protein 2 | SORBS2 | 1 | 1 | 124,0 | -0,18 | 0,905 | 0,35 | 0,749 |
| Q00796 | Sorbitol dehydrogenase | SORD | 4 | 4 | 38,3 | 0,04 | 0,352 | -0,01 | 0,920 |
| P30626 | Sorcin | SRI | 2 | 2 | 21,7 | 0,17 | 0,111 | -0,23 | 0,487 |
| Q13596 | Sorting nexin-1 | SNX1 | 1 | 1 | 59,0 | -0,27 | 0,340 | -0,07 | 0,755 |
| Q15036 | Sorting nexin-17 | SNX17 | 1 | 1 | 52,9 | -0,38 | 0,504 | 0,00 | 0,939 |
| Q96RF0 | Sorting nexin-18 | SNX18 | 4 | 4 | 68,9 | 0,12 | 0,673 | -0,44 | 0,190 |
| O60749 | Sorting nexin-2 | SNX2 | 2 | 2 | 58,4 | 0,00 | 0,815 | -0,66 | 0,250 |
| Q96L92 | Sorting nexin-27 | SNX27 | 2 | 2 | 61,2 | 0,39 | 0,230 | -1,12 | 0,079 |
| Q8WV41 | Sorting nexin-33 | SNX33 | 1 | 1 | 65,2 | 0,11 | 0,553 | -0,03 | 0,873 |
| Q9UNH7 | Sorting nexin-6 | SNX6 | 2 | 2 | 46,6 | 0,37 | 0,129 | 0,01 | 0,903 |
| Q9Y5X1 | Sorting nexin-9 | SNX9 | 1 | 1 | 66,5 | 0,47 | 0,258 | 0,08 | 0,907 |
| Q8N0X7 | Spartin | SPG20 | 1 | 1 | 72,8 | 0,12 | 0,336 | 0,36 | 0,852 |
| Q9UBP0 | Spastin | SPAST | 2 | 2 | 67,2 | 0,75 | 0,182 | 0,17 | 0,605 |
| Q9BW04 | Specifically androgen-regulated gene protein | SARG | 9 | 9 | 63,9 | -0,59 | 0,065 | -0,23 | 0,303 |
| Q13813 | Spectrin alpha chain, non-erythrocytic 1 | SPTAN1 | 1 | 1 | 284,4 | 1,02 | 0,171 | 0,31 | 0,786 |
| Q01082 | Spectrin beta chain, non-erythrocytic 1 | SPTBN1 | 4 | 4 | 274,4 | 0,14 | 0,556 | -0,48 | 0,044 |
| Q07617 | Sperm-associated antigen 1 | SPAG1 | 4 | 4 | 103,6 | 0,16 | 0,018 | -0,09 | 0,657 |
| Q6Q759 | Sperm-associated antigen 17 | SPAG17 | 1 | 1 | 251,6 | -0,48 | 0,364 | 0,16 | 0,923 |
| Q96N06 | Spermatogenesis-associated protein 33 | SPATA33 | 1 | 1 | 15,5 | -0,28 | 0,231 | -0,10 | 0,521 |
| P63208 | S-phase kinase-associated protein 1 | SKP1 | 1 | 1 | 18,6 | 0,12 | 0,202 | -0,58 | 0,402 |
| Q9NY59 | Sphingomyelin phosphodiesterase 3 | SMPD3 | 2 | 2 | 71,0 | -0,28 | 0,147 | -0,52 | 0,090 |
| Q14247 | Src substrate cortactin | CTTN | 10 | 10 | 61,5 | -0,23 | 0,473 | -0,38 | 0,072 |
| O95630 | STAM-binding protein | STAMBP | 1 | 1 | 48,0 | -1,19 | 0,119 | -0,38 | 0,514 |
| H3BQB6 | Stathmin domain-containing protein 1 | STMND1 | 1 | 1 | 31,0 | -0,63 | 0,345 | -0,76 | 0,123 |
| Q9H2G2 | STE20-like serine/threonine-protein kinase | SLK | 15 | 15 | 142,6 | -0,50 | 0,190 | 0,12 | 0,389 |
| Q5K651 | Sterile alpha motif domain-containing protein 9 | SAMD9 | 1 | 1 | 184,2 | -1,09 | 0,867 | -1,46 | 0,546 |
| Q8TAV4 | Stomatin-like protein 3 | STOML3 | 2 | 2 | 32,1 | 0,30 | 0,222 | 0,17 | 0,429 |
| P31948 | Stress-induced-phosphoprotein 1 | STIP1 | 4 | 4 | 62,6 | 0,35 | 0,110 | 0,02 | 0,759 |
| P09238 | Stromelysin-2 | MMP10 | 1 | 1 | 54,1 | 0,59 | 0,058 | -0,43 | 0,392 |
| P25103 | Substance-P receptor | TACR1 | 1 | 1 | 46,2 | -0,77 | 0,092 | -0,39 | 0,911 |
| O00391 | Sulfhydryl oxidase 1 | QSOX1 | 2 | 2 | 82,5 | 0,00 | 0,935 | 0,09 | 0,879 |
| P0DMM9 | Sulfotransferase 1A3 | SULT1A3 | 2 | 2 | 34,2 | 0,20 | 0,255 | -0,06 | 0,663 |
| P00441 | Superoxide dismutase [Cu-Zn] | SOD1 | 3 | 3 | 15,9 | 0,28 | 0,540 | -0,05 | 0,868 |
| Q9UGT4 | Sushi domain-containing protein 2 | SUSD2 | 2 | 2 | 90,1 | 0,38 | 0,397 | 3,75 | 0,020 |
| O60687 | Sushi repeat-containing protein SRPX2 | SRPX2 | 1 | 1 | 52,9 | 0,00 | 0,619 | -0,42 | 0,594 |
| Q9UH65 | Switch-associated protein 70 | SWAP70 | 9 | 9 | 69,0 | 1,72 | 0,071 | 0,30 | 0,475 |
| Q99536 | Synaptic vesicle membrane protein VAT-1 homolog | VAT1 | 1 | 1 | 41,9 | -0,33 | 0,305 | -0,05 | 0,889 |
| O15498 | Synaptobrevin homolog YKT6 | YKT6 | 3 | 3 | 22,4 | -0,17 | 0,324 | -0,12 | 0,509 |
| O43760 | Synaptogyrin-2 | SYNGR2 | 1 | 1 | 24,8 | 0,29 | 0,514 | -0,16 | 0,244 |
| O00161 | Synaptosomal-associated protein 23 | SNAP23 | 2 | 2 | 23,3 | -0,31 | 0,165 | -0,31 | 0,651 |
| O95721 | Synaptosomal-associated protein 29 | SNAP29 | 1 | 1 | 29,0 | 0,30 | 0,098 | -0,31 | 0,601 |
| Q8IYJ3 | Synaptotagmin-like protein 1 | SYTL1 | 7 | 7 | 61,8 | -0,45 | 0,023 | -0,47 | 0,219 |
| Q9HCH5 | Synaptotagmin-like protein 2 | SYTL2 | 7 | 7 | 104,9 | -0,30 | 0,403 | -0,41 | 0,467 |
| Q4VX76 | Synaptotagmin-like protein 3 | SYTL3 | 2 | 2 | 68,5 | -0,59 | 0,180 | -0,23 | 0,592 |
| Q96C24 | Synaptotagmin-like protein 4 | SYTL4 | 4 | 4 | 76,0 | -0,57 | 0,270 | -0,21 | 0,857 |
| Q8TDW5 | Synaptotagmin-like protein 5 | SYTL5 | 8 | 8 | 81,5 | -0,78 | 0,063 | -0,63 | 0,164 |
| O75558 | Syntaxin-11 | STX11 | 3 | 3 | 33,2 | 0,86 | 0,071 | -0,63 | 0,153 |
| Q8N4C7 | Syntaxin-19 | STX19 | 3 | 3 | 34,3 | -0,19 | 0,320 | 0,20 | 0,194 |
| P32856 | Syntaxin-2 | STX2 | 3 | 3 | 33,3 | -0,04 | 0,952 | -0,37 | 0,088 |
| Q13277 | Syntaxin-3 | STX3 | 4 | 4 | 33,1 | -0,25 | 0,429 | -0,34 | 0,206 |
| O15400 | Syntaxin-7 | STX7 | 4 | 4 | 29,8 | 0,24 | 0,262 | -0,44 | 0,129 |
| P61764 | Syntaxin-binding protein 1 | STXBP1 | 2 | 2 | 67,5 | -0,21 | 0,478 | -0,05 | 0,980 |
| Q15833 | Syntaxin-binding protein 2 | STXBP2 | 7 | 7 | 66,4 | -0,27 | 0,249 | -0,19 | 0,066 |
| O00186 | Syntaxin-binding protein 3 | STXBP3 | 2 | 2 | 67,7 | -0,05 | 0,830 | 0,05 | 0,751 |
| Q6ZWJ1 | Syntaxin-binding protein 4 | STXBP4 | 1 | 1 | 61,6 | -0,03 | 0,950 | -0,61 | 0,039 |
| Q5T5C0 | Syntaxin-binding protein 5 | STXBP5 | 1 | 1 | 127,5 | 0,27 | 0,826 | -0,18 | 0,737 |
| Q9H190 | Syntenin-2 | SDCBP2 | 2 | 2 | 31,6 | 1,44 | 0,045 | 0,93 | 0,058 |
| Q9Y490 | Talin-1 | TLN1 | 7 | 7 | 269,6 | 0,13 | 0,333 | 0,21 | 0,222 |
| Q8N9U0 | Tandem C2 domains nuclear protein | TC2N | 3 | 3 | 55,2 | 0,03 | 0,714 | -0,16 | 0,506 |
| O60784 | Target of Myb protein 1 | TOM1 | 1 | 1 | 53,8 | 1,67 | 0,237 | 0,61 | 0,117 |
| Q92804 | TATA-binding protein-associated factor 2N | TAF15 | 3 | 3 | 61,8 | -0,33 | 0,370 | 0,72 | 0,389 |
| O14907 | Tax1-binding protein 3 | TAX1BP3 | 1 | 1 | 13,7 | -0,51 | 0,235 | -0,07 | 0,957 |
| Q9BXI6 | TBC1 domain family member 10A | TBC1D10A | 3 | 3 | 57,1 | -0,18 | 0,468 | -0,18 | 0,611 |
| Q4KMP7 | TBC1 domain family member 10B | TBC1D10B | 2 | 2 | 87,1 | -0,85 | 0,023 | -0,42 | 0,407 |
| Q9ULP9 | TBC1 domain family member 24 | TBC1D24 | 2 | 2 | 62,9 | -0,16 | 0,529 | -0,23 | 0,169 |
| P17987 | T-complex protein 1 subunit alpha | TCP1 | 3 | 3 | 60,3 | 0,37 | 0,465 | 0,17 | 0,468 |
| P78371 | T-complex protein 1 subunit beta | CCT2 | 3 | 3 | 57,5 | 0,35 | 0,411 | 0,23 | 0,106 |
| P50991 | T-complex protein 1 subunit delta | CCT4 | 3 | 3 | 57,9 | 0,14 | 0,508 | 0,39 | 0,324 |
| Q99832 | T-complex protein 1 subunit eta | CCT7 | 1 | 1 | 59,3 | 0,91 | 0,187 | 0,18 | 0,919 |
| P49368 | T-complex protein 1 subunit gamma | CCT3 | 1 | 1 | 60,5 | 0,84 | 0,065 | 0,21 | 0,919 |
| P50990 | T-complex protein 1 subunit theta | CCT8 | 3 | 3 | 59,6 | -0,47 | 0,567 | 0,47 | 0,286 |
| P40227 | T-complex protein 1 subunit zeta | CCT6A | 2 | 2 | 58,0 | 0,86 | 0,122 | 0,56 | 0,240 |
| Q7Z6L1 | Tectonin beta-propeller repeat-containing protein 1 | TECPR1 | 1 | 1 | 129,6 | -0,28 | 0,593 | 0,34 | 0,288 |
| P24821 | Tenascin | TNC | 9 | 9 | 240,7 | -0,25 | 0,702 | -0,16 | 0,596 |
| Q9UGI8 | Testin | TES | 3 | 3 | 48,0 | 0,16 | 0,235 | 0,36 | 0,106 |
| Q8NG11 | Tetraspanin-14 | TSPAN14 | 1 | 1 | 30,7 | -0,50 | 0,183 | -0,20 | 0,799 |
| O43657 | Tetraspanin-6 | TSPAN6 | 2 | 2 | 27,5 | -0,37 | 0,577 | -0,76 | 0,118 |
| P19075 | Tetraspanin-8 | TSPAN8 | 1 | 1 | 26,0 | -0,40 | 0,653 | 0,96 | 0,119 |
| Q7Z4L5 | Tetratricopeptide repeat protein 21B | TTC21B | 2 | 2 | 150,8 | 0,04 | 0,886 | 0,23 | 0,651 |
| Q96NG3 | Tetratricopeptide repeat protein 25 | TTC25 | 2 | 2 | 76,6 | -0,24 | 0,832 | -0,22 | 0,475 |
| Q6PGP7 | Tetratricopeptide repeat protein 37 | TTC37 | 1 | 1 | 175,4 | 0,17 | 0,573 | 0,42 | 0,558 |
| Q9ULT0 | Tetratricopeptide repeat protein 7A | TTC7A | 4 | 4 | 96,1 | -0,18 | 0,161 | 0,56 | 0,009 |
| Q8TAM2 | Tetratricopeptide repeat protein 8 | TTC8 | 1 | 1 | 61,5 | 0,12 | 0,414 | -0,15 | 0,962 |
| Q92623 | Tetratricopeptide repeat protein 9A | TTC9 | 3 | 3 | 24,4 | 0,20 | 0,435 | 0,13 | 0,702 |
| P52888 | Thimet oligopeptidase | THOP1 | 1 | 1 | 78,8 | 0,69 | 0,236 | 0,32 | 0,650 |
| P10599 | Thioredoxin | TXN | 2 | 2 | 11,7 | -0,05 | 0,726 | -0,47 | 0,517 |
| Q16881 | Thioredoxin reductase 1, cytoplasmic | TXNRD1 | 2 | 2 | 70,9 | 0,16 | 0,418 | -0,33 | 0,332 |
| Q8NFU3 | Thiosulfate sulfurtransferase/rhodanese-like domain-containing protein 1 | TSTD1 | 2 | 2 | 12,5 | 0,02 | 0,922 | -0,10 | 0,706 |
| P26639 | Threonine--tRNA ligase, cytoplasmic | TARS | 4 | 4 | 83,4 | 0,35 | 0,423 | 0,17 | 0,497 |
| P07996 | Thrombospondin-1 | THBS1 | 3 | 3 | 129,3 | -0,24 | 0,385 | 0,87 | 0,195 |
| P19971 | Thymidine phosphorylase | TYMP | 4 | 4 | 49,9 | 0,20 | 0,478 | -0,01 | 0,844 |
| Q9UDY2 | Tight junction protein ZO-2 | TJP2 | 5 | 5 | 133,9 | 0,01 | 0,797 | 0,01 | 0,894 |
| O95049 | Tight junction protein ZO-3 | TJP3 | 4 | 4 | 101,3 | -0,12 | 0,593 | -0,26 | 0,407 |
| P13726 | Tissue factor | F3 | 2 | 2 | 33,0 | -0,75 | 0,339 | 0,38 | 0,591 |
| Q6P9B6 | TLD domain-containing protein 1 | TLDC1 | 3 | 3 | 51,0 | -0,15 | 0,560 | -0,24 | 0,333 |
| Q15025 | TNFAIP3-interacting protein 1 | TNIP1 | 1 | 1 | 71,8 | 3,55 | 0,016 | 0,16 | 0,924 |
| Q9H0E2 | Toll-interacting protein | TOLLIP | 4 | 4 | 30,3 | 0,12 | 0,460 | 0,30 | 0,313 |
| Q15399 | Toll-like receptor 1 | TLR1 | 1 | 1 | 90,2 | -0,32 | 0,277 | -0,59 | 0,170 |
| O60603 | Toll-like receptor 2 | TLR2 | 3 | 3 | 89,8 | 0,50 | 0,159 | -0,35 | 0,405 |
| O60602 | Toll-like receptor 5 | TLR5 | 2 | 2 | 97,8 | 0,00 | 0,927 | -0,07 | 0,751 |
| O75674 | TOM1-like protein 1 | TOM1L1 | 3 | 3 | 53,0 | -0,04 | 0,948 | 0,01 | 0,741 |
| Q6ZVM7 | TOM1-like protein 2 | TOM1L2 | 1 | 1 | 55,5 | -0,41 | 0,245 | -0,33 | 0,314 |
| Q8TDR0 | TRAF3-interacting protein 1 | TRAF3IP1 | 1 | 1 | 78,6 | 0,00 | 0,906 | -0,01 | 0,876 |
| P37837 | Transaldolase | TALDO1 | 1 | 1 | 37,5 | 0,22 | 0,079 | 0,04 | 0,733 |
| P37802 | Transgelin-2 | TAGLN2 | 4 | 4 | 22,4 | -0,05 | 0,862 | -0,54 | 0,117 |
| P55072 | Transitional endoplasmic reticulum ATPase | VCP | 3 | 3 | 89,3 | 0,45 | 0,141 | 0,29 | 0,059 |
| P29401 | Transketolase | TKT | 5 | 5 | 67,8 | -0,20 | 0,151 | 0,22 | 0,020 |
| Q7Z404 | Transmembrane channel-like protein 4 | TMC4 | 4 | 4 | 79,2 | -0,08 | 0,655 | -0,93 | 0,002 |
| Q6UXY8 | Transmembrane channel-like protein 5 | TMC5 | 13 | 13 | 114,7 | -0,08 | 0,477 | -0,69 | 0,127 |
| Q15363 | Transmembrane emp24 domain-containing protein 2 | TMED2 | 1 | 1 | 22,7 | -0,53 | 0,498 | 0,30 | 0,583 |
| Q9BVK6 | Transmembrane emp24 domain-containing protein 9 | TMED9 | 1 | 1 | 27,3 | 0,61 | 0,807 | 0,76 | 0,514 |
| O15393 | Transmembrane protease serine 2 | TMPRSS2 | 3 | 3 | 53,8 | -0,15 | 0,987 | -0,01 | 0,794 |
| Q86X19 | Transmembrane protein 17 | TMEM17 | 1 | 1 | 23,0 | 0,71 | 0,725 | -0,46 | 0,208 |
| A6NML5 | Transmembrane protein 212 | TMEM212 | 1 | 1 | 21,4 | 0,09 | 0,453 | -0,41 | 0,128 |
| Q9H6L2 | Transmembrane protein 231 | TMEM231 | 1 | 1 | 36,0 | -0,15 | 0,881 | 0,05 | 0,726 |
| C9JI98 | Transmembrane protein 238 | TMEM238 | 2 | 2 | 18,0 | -0,55 | 0,008 | 0,42 | 0,666 |
| Q9H2D6 | TRIO and F-actin-binding protein | TRIOBP | 1 | 1 | 261,2 | -0,13 | 0,426 | -0,07 | 0,679 |
| Q3LXA3 | Triokinase/FMN cyclase | TKFC | 3 | 3 | 58,9 | -0,20 | 0,243 | -0,35 | 0,051 |
| P60174 | Triosephosphate isomerase | TPI1 | 3 | 3 | 30,8 | -0,24 | 0,235 | 0,04 | 0,922 |
| Q14134 | Tripartite motif-containing protein 29 | TRIM29 | 1 | 1 | 65,8 | 0,30 | 0,233 | -0,04 | 0,581 |
| Q9Y3I0 | tRNA-splicing ligase RtcB homolog | RTCB | 1 | 1 | 55,2 | -0,19 | 0,616 | -0,16 | 0,696 |
| Q13641 | Trophoblast glycoprotein | TPBG | 2 | 2 | 46,0 | 0,27 | 0,317 | -0,51 | 0,337 |
| P06753 | Tropomyosin alpha-3 chain | TPM3 | 1 | 4 | 32,9 | 0,59 | 0,185 | -0,31 | 0,450 |
| P67936 | Tropomyosin alpha-4 chain | TPM4 | 2 | 5 | 28,5 | -0,26 | 0,912 | -0,59 | 0,713 |
| P23381 | Tryptophan--tRNA ligase, cytoplasmic | WARS | 1 | 1 | 53,1 | 1,03 | 0,135 | 0,84 | 0,402 |
| P50607 | Tubby protein homolog | TUB | 3 | 3 | 55,6 | -0,44 | 0,321 | -0,19 | 0,537 |
| Q9BQE3 | Tubulin alpha-1C chain | TUBA1C | 3 | 3 | 49,9 | -0,05 | 0,738 | -0,25 | 0,542 |
| P68371 | Tubulin beta-4B chain | TUBB4B | 5 | 5 | 49,8 | -0,29 | 0,821 | -0,19 | 0,518 |
| Q9BW30 | Tubulin polymerization-promoting protein family member 3 | TPPP3 | 7 | 7 | 19,0 | -0,18 | 0,713 | -0,66 | 0,020 |
| O94811 | Tubulin polymerization-promoting protein | TPPP | 1 | 1 | 23,7 | 0,47 | 0,109 | 1,26 | 0,009 |
| Q99426 | Tubulin-folding cofactor B | TBCB | 2 | 2 | 27,3 | 0,28 | 0,593 | 0,13 | 0,680 |
| O75347 | Tubulin-specific chaperone A | TBCA | 1 | 1 | 12,8 | 1,74 | 0,414 | -0,03 | 0,954 |
| Q03169 | Tumor necrosis factor alpha-induced protein 2 | TNFAIP2 | 4 | 4 | 72,6 | 0,70 | 0,125 | -0,62 | 0,625 |
| P21580 | Tumor necrosis factor alpha-induced protein 3 | TNFAIP3 | 2 | 2 | 89,6 | 1,88 | 0,021 | 0,08 | 0,667 |
| O95379 | Tumor necrosis factor alpha-induced protein 8 | TNFAIP8 | 1 | 1 | 23,0 | -0,07 | 0,695 | -0,03 | 0,860 |
| Q8WVP5 | Tumor necrosis factor alpha-induced protein 8-like protein 1 | TNFAIP8L1 | 1 | 1 | 20,8 | -0,29 | 0,077 | -0,87 | 0,061 |
| P50591 | Tumor necrosis factor ligand superfamily member 10 | TNFSF10 | 2 | 2 | 32,5 | -0,39 | 0,294 | 0,31 | 0,485 |
| P19438 | Tumor necrosis factor receptor superfamily member 1A | TNFRSF1A | 1 | 1 | 50,5 | 2,12 | 0,005 | 0,21 | 0,482 |
| Q99816 | Tumor susceptibility gene 101 protein | TSG101 | 6 | 6 | 43,9 | 0,08 | 0,294 | 0,06 | 0,228 |
| P09758 | Tumor-associated calcium signal transducer 2 | TACSTD2 | 2 | 2 | 35,7 | -0,19 | 0,966 | -0,59 | 0,288 |
| Q12792 | Twinfilin-1 | TWF1 | 3 | 3 | 40,3 | 0,03 | 0,693 | -0,15 | 0,424 |
| Q14642 | Type I inositol 1,4,5-trisphosphate 5-phosphatase | INPP5A | 2 | 2 | 47,8 | -0,37 | 0,182 | -0,15 | 0,058 |
| P41240 | Tyrosine-protein kinase CSK | CSK | 2 | 2 | 50,7 | -0,13 | 0,453 | -0,22 | 0,594 |
| P42685 | Tyrosine-protein kinase FRK | FRK | 8 | 9 | 58,2 | 0,62 | 0,037 | 0,12 | 0,728 |
| P23458 | Tyrosine-protein kinase JAK1 | JAK1 | 1 | 1 | 133,2 | 0,01 | 0,920 | -0,86 | 0,151 |
| O60674 | Tyrosine-protein kinase JAK2 | JAK2 | 1 | 1 | 130,6 | -0,31 | 0,622 | 0,76 | 0,157 |
| P06239 | Tyrosine-protein kinase Lck | LCK | 1 | 2 | 58,0 | 0,15 | 0,666 | -0,64 | 0,311 |
| P07948 | Tyrosine-protein kinase Lyn | LYN | 8 | 9 | 58,5 | 0,27 | 0,004 | -0,02 | 0,921 |
| Q6J9G0 | Tyrosine-protein kinase STYK1 | STYK1 | 1 | 1 | 47,5 | 0,47 | 0,339 | 0,07 | 0,981 |
| P43405 | Tyrosine-protein kinase SYK | SYK | 1 | 1 | 72,0 | -0,92 | 0,613 | -0,44 | 0,990 |
| P07947 | Tyrosine-protein kinase Yes | YES1 | 4 | 6 | 60,8 | -0,12 | 0,570 | 0,00 | 0,871 |
| Q06124 | Tyrosine-protein phosphatase non-receptor type 11 | PTPN11 | 2 | 2 | 68,4 | 0,12 | 0,403 | -0,18 | 0,085 |
| Q12923 | Tyrosine-protein phosphatase non-receptor type 13 | PTPN13 | 4 | 4 | 276,7 | -0,20 | 0,233 | 0,04 | 0,983 |
| Q9H3S7 | Tyrosine-protein phosphatase non-receptor type 23 | PTPN23 | 2 | 2 | 178,9 | 0,41 | 0,451 | 0,28 | 0,276 |
| P29350 | Tyrosine-protein phosphatase non-receptor type 6 | PTPN6 | 3 | 3 | 67,5 | -0,09 | 0,814 | -0,38 | 0,153 |
| P78324 | Tyrosine-protein phosphatase non-receptor type substrate 1 | SIRPA | 3 | 3 | 54,9 | -0,85 | 0,122 | -0,29 | 0,866 |
| P54577 | Tyrosine--tRNA ligase, cytoplasmic | YARS | 1 | 1 | 59,1 | 0,73 | 0,195 | 0,09 | 0,945 |
| P45974 | Ubiquitin carboxyl-terminal hydrolase 5 | USP5 | 4 | 4 | 95,7 | 0,16 | 0,241 | 0,12 | 0,125 |
| Q8N5J2 | Ubiquitin carboxyl-terminal hydrolase MINDY-1 | FAM63A | 2 | 2 | 51,7 | -0,44 | 0,195 | -0,11 | 0,800 |
| Q8WUN7 | Ubiquitin domain-containing protein 2 | UBTD2 | 2 | 2 | 26,2 | -0,18 | 0,221 | -0,34 | 0,007 |
| O14562 | Ubiquitin domain-containing protein UBFD1 | UBFD1 | 1 | 1 | 33,4 | -0,38 | 0,253 | -0,17 | 0,499 |
| Q96FW1 | Ubiquitin thioesterase OTUB1 | OTUB1 | 1 | 1 | 31,3 | 0,20 | 0,725 | 0,49 | 0,426 |
| Q9NZ09 | Ubiquitin-associated protein 1 | UBAP1 | 1 | 1 | 55,0 | 1,01 | 0,177 | 0,41 | 0,159 |
| P62837 | Ubiquitin-conjugating enzyme E2 D2 | UBE2D2 | 1 | 1 | 16,7 | 0,00 | 0,988 | 0,16 | 0,166 |
| P68036 | Ubiquitin-conjugating enzyme E2 L3 | UBE2L3 | 1 | 1 | 17,9 | -0,02 | 0,869 | 0,20 | 0,047 |
| P61088 | Ubiquitin-conjugating enzyme E2 N | UBE2N | 3 | 3 | 17,1 | -0,26 | 0,195 | -0,12 | 0,456 |
| Q13404 | Ubiquitin-conjugating enzyme E2 variant 1 | UBE2V1 | 1 | 1 | 16,5 | 0,15 | 0,219 | -0,11 | 0,806 |
| Q15819 | Ubiquitin-conjugating enzyme E2 variant 2 | UBE2V2 | 1 | 1 | 16,4 | -0,13 | 0,200 | -0,44 | 0,232 |
| Q8IX04 | Ubiquitin-conjugating enzyme E2 variant 3 | UEVLD | 1 | 1 | 52,2 | 0,05 | 0,675 | -0,16 | 0,849 |
| P22314 | Ubiquitin-like modifier-activating enzyme 1 | UBA1 | 7 | 7 | 117,8 | 0,23 | 0,261 | 0,20 | 0,105 |
| A0AVT1 | Ubiquitin-like modifier-activating enzyme 6 | UBA6 | 3 | 3 | 117,9 | 0,26 | 0,200 | 0,08 | 0,901 |
| O95164 | Ubiquitin-like protein 3 | UBL3 | 1 | 1 | 13,1 | -0,30 | 0,461 | -0,49 | 0,263 |
| Q9BZV1 | UBX domain-containing protein 6 | UBXN6 | 1 | 1 | 49,7 | -0,85 | 0,356 | -0,50 | 0,781 |
| Q6UX72 | UDP-GlcNAc:betaGal beta-1,3-N-acetylglucosaminyltransferase 9 | B3GNT9 | 1 | 1 | 43,7 | 0,54 | 0,689 | -0,29 | 0,642 |
| Q14376 | UDP-glucose 4-epimerase | GALE | 1 | 1 | 38,3 | 0,12 | 0,162 | 0,33 | 0,396 |
| O60701 | UDP-glucose 6-dehydrogenase | UGDH | 2 | 2 | 55,0 | -0,03 | 0,873 | 0,13 | 0,853 |
| Q16222 | UDP-N-acetylhexosamine pyrophosphorylase | UAP1 | 3 | 3 | 58,7 | -0,04 | 0,998 | 0,99 | 0,023 |
| Q96A22 | Uncharacterized protein C11orf52 | C11orf52 | 1 | 1 | 13,9 | -0,25 | 0,750 | -0,44 | 0,136 |
| Q5VWT5 | Uncharacterized protein C1orf168 | C1orf168 | 2 | 2 | 82,0 | -0,87 | 0,200 | -1,00 | 0,068 |
| A4QMS7 | Uncharacterized protein C5orf49 | C5orf49 | 1 | 1 | 17,0 | -0,08 | 0,952 | -0,62 | 0,582 |
| Q5T0Z8 | Uncharacterized protein C6orf132 | C6orf132 | 11 | 11 | 124,0 | -0,65 | 0,494 | -0,41 | 0,388 |
| Q6NV74 | Uncharacterized protein KIAA1211-like | KIAA1211L | 5 | 5 | 102,1 | -0,80 | 0,247 | -0,54 | 0,009 |
| Q9P206 | Uncharacterized protein KIAA1522 | KIAA1522 | 5 | 5 | 107,0 | -0,79 | 0,134 | -0,18 | 0,520 |
| O43795 | Unconventional myosin-Ib | MYO1B | 13 | 13 | 131,9 | -0,13 | 0,618 | 0,00 | 0,897 |
| O00159 | Unconventional myosin-Ic | MYO1C | 12 | 12 | 121,6 | -0,32 | 0,080 | 0,42 | 0,030 |
| O94832 | Unconventional myosin-Id | MYO1D | 16 | 16 | 116,1 | -0,27 | 0,214 | -0,13 | 0,502 |
| Q12965 | Unconventional myosin-Ie | MYO1E | 3 | 3 | 127,0 | 0,00 | 0,587 | 0,35 | 0,795 |
| Q9Y4I1 | Unconventional myosin-Va | MYO5A | 5 | 7 | 215,3 | -0,26 | 0,154 | 0,00 | 0,961 |
| Q9ULV0 | Unconventional myosin-Vb | MYO5B | 9 | 11 | 213,5 | -0,08 | 0,866 | -0,07 | 0,417 |
| Q9NQX4 | Unconventional myosin-Vc | MYO5C | 10 | 12 | 202,7 | -0,16 | 0,841 | -0,51 | 0,556 |
| Q9UM54 | Unconventional myosin-VI | MYO6 | 8 | 8 | 149,6 | 0,34 | 0,021 | 0,41 | 0,024 |
| Q9HD67 | Unconventional myosin-X | MYO10 | 4 | 4 | 237,2 | 0,32 | 0,128 | 0,04 | 0,822 |
| Q9Y224 | UPF0568 protein C14orf166 | C14orf166 | 2 | 2 | 28,1 | 0,01 | 0,920 | 0,05 | 0,774 |
| O75841 | Uroplakin-1b | UPK1B | 2 | 2 | 29,6 | -0,73 | 0,192 | -0,20 | 0,703 |
| Q9BT76 | Uroplakin-3b | UPK3B | 1 | 1 | 33,9 | -1,15 | 0,089 | -0,63 | 0,362 |
| B0FP48 | Uroplakin-3b-like protein | UPK3BL | 4 | 4 | 28,4 | -0,88 | 0,128 | -0,28 | 0,214 |
| Q92738 | USP6 N-terminal-like protein | USP6NL | 1 | 1 | 94,0 | -1,26 | 0,542 | -1,28 | 0,627 |
| P11684 | Uteroglobin | SCGB1A1 | 2 | 2 | 10,0 | -0,14 | 0,801 | -0,58 | 0,234 |
| Q16851 | UTP--glucose-1-phosphate uridylyltransferase | UGP2 | 3 | 3 | 56,9 | 0,43 | 0,049 | 0,56 | 0,051 |
| P54727 | UV excision repair protein RAD23 homolog B | RAD23B | 3 | 3 | 43,1 | -0,02 | 0,983 | 0,09 | 0,690 |
| Q7L1V2 | Vacuolar fusion protein MON1 homolog B | MON1B | 1 | 1 | 59,2 | 1,83 | 0,055 | -0,17 | 0,648 |
| Q709C8 | Vacuolar protein sorting-associated protein 13C | VPS13C | 6 | 6 | 422,1 | 0,57 | 0,005 | 0,02 | 0,750 |
| O75436 | Vacuolar protein sorting-associated protein 26A | VPS26A | 2 | 2 | 38,1 | 0,47 | 0,101 | 0,24 | 0,439 |
| Q9UK41 | Vacuolar protein sorting-associated protein 28 homolog | VPS28 | 2 | 2 | 25,4 | 0,32 | 0,261 | -0,17 | 0,796 |
| Q9H9H4 | Vacuolar protein sorting-associated protein 37B | VPS37B | 3 | 3 | 31,3 | 0,06 | 0,531 | 0,39 | 0,187 |
| A5D8V6 | Vacuolar protein sorting-associated protein 37C | VPS37C | 2 | 2 | 38,6 | -0,14 | 0,946 | -0,01 | 0,953 |
| Q9UN37 | Vacuolar protein sorting-associated protein 4A | VPS4A | 2 | 2 | 48,9 | -0,07 | 0,927 | 0,10 | 0,674 |
| O75351 | Vacuolar protein sorting-associated protein 4B | VPS4B | 5 | 5 | 49,3 | -0,05 | 0,998 | 0,26 | 0,326 |
| Q9NP79 | Vacuolar protein sorting-associated protein VTA1 homolog | VTA1 | 1 | 1 | 33,9 | 0,03 | 0,851 | -0,11 | 0,840 |
| Q86VN1 | Vacuolar protein-sorting-associated protein 36 | VPS36 | 3 | 3 | 43,8 | 0,34 | 0,010 | -0,13 | 0,396 |
| Q96H20 | Vacuolar-sorting protein SNF8 | SNF8 | 2 | 2 | 28,8 | 0,36 | 0,224 | -0,01 | 0,898 |
| Q14508 | WAP four-disulfide core domain protein 2 | WFDC2 | 1 | 1 | 13,0 | 0,32 | 0,489 | -0,40 | 0,510 |
| Q8TF74 | WAS/WASL-interacting protein family member 2 | WIPF2 | 3 | 3 | 46,3 | -0,33 | 0,198 | 0,05 | 0,993 |
| O95498 | Vascular non-inflammatory molecule 2 | VNN2 | 1 | 1 | 58,5 | 0,99 | 0,008 | -0,42 | 0,653 |
| Q8IWB7 | WD repeat and FYVE domain-containing protein 1 | WDFY1 | 1 | 1 | 46,3 | 0,23 | 0,115 | -0,53 | 0,219 |
| O75083 | WD repeat-containing protein 1 | WDR1 | 2 | 2 | 66,2 | 0,40 | 0,252 | -0,56 | 0,390 |
| Q8NEZ3 | WD repeat-containing protein 19 | WDR19 | 4 | 4 | 151,5 | -0,02 | 0,879 | -0,13 | 0,363 |
| Q9P2L0 | WD repeat-containing protein 35 | WDR35 | 3 | 3 | 133,5 | 0,45 | 0,188 | 0,00 | 0,959 |
| O94967 | WD repeat-containing protein 47 | WDR47 | 1 | 1 | 101,9 | -0,10 | 0,820 | 0,77 | 0,003 |
| Q8WVS4 | WD repeat-containing protein 60 | WDR60 | 1 | 1 | 122,5 | -0,26 | 0,506 | 0,39 | 0,643 |
| O95562 | Vesicle transport protein SFT2B | SFT2D2 | 1 | 1 | 17,8 | 0,82 | 0,051 | 0,35 | 0,137 |
| Q15836 | Vesicle-associated membrane protein 3 | VAMP3 | 1 | 1 | 11,3 | 0,12 | 0,326 | -0,09 | 0,590 |
| O95183 | Vesicle-associated membrane protein 5 | VAMP5 | 1 | 1 | 12,8 | -0,53 | 0,100 | 1,84 | 0,082 |
| Q9BV40 | Vesicle-associated membrane protein 8 | VAMP8 | 3 | 3 | 11,4 | 0,22 | 0,155 | -0,30 | 0,399 |
| P46459 | Vesicle-fusing ATPase | NSF | 5 | 5 | 82,5 | -0,09 | 0,542 | -0,10 | 0,136 |
| O15195 | Villin-like protein | VILL | 10 | 10 | 95,8 | -0,17 | 0,917 | -0,39 | 0,150 |
| P18206 | Vinculin | VCL | 12 | 12 | 123,7 | -0,20 | 0,433 | 0,31 | 0,169 |
| Q9Y6W5 | Wiskott-Aldrich syndrome protein family member 2 | WASF2 | 5 | 5 | 54,3 | -0,25 | 0,499 | -0,28 | 0,310 |
| Q7Z5L0 | Vitelline membrane outer layer protein 1 homolog | VMO1 | 1 | 1 | 21,5 | -1,56 | 0,057 | -0,64 | 0,724 |
| P21796 | Voltage-dependent anion-selective channel protein 1 | VDAC1 | 1 | 1 | 30,8 | 0,03 | 0,898 | 0,10 | 0,948 |
| Q96D96 | Voltage-gated hydrogen channel 1 | HVCN1 | 1 | 1 | 31,7 | 1,29 | 0,116 | 0,69 | 0,121 |
| O00534 | von Willebrand factor A domain-containing protein 5A | VWA5A | 4 | 4 | 86,4 | 0,24 | 0,193 | 0,17 | 0,673 |
| Q9Y2B5 | VPS9 domain-containing protein 1 | VPS9D1 | 1 | 1 | 68,9 | 0,01 | 0,595 | -0,45 | 0,979 |
| Q7Z7D3 | V-set domain-containing T-cell activation inhibitor 1 | VTCN1 | 1 | 1 | 30,9 | -1,03 | 0,126 | -1,06 | 0,075 |
| Q9HBG4 | V-type proton ATPase 116 kDa subunit a isoform 4 | ATP6V0A4 | 1 | 1 | 96,3 | 0,29 | 0,329 | -0,56 | 0,844 |
| P38606 | V-type proton ATPase catalytic subunit A | ATP6V1A | 1 | 1 | 68,3 | -0,52 | 0,318 | -0,76 | 0,787 |
| P21281 | V-type proton ATPase subunit B, brain isoform | ATP6V1B2 | 3 | 5 | 56,5 | 0,03 | 0,532 | 0,23 | 0,449 |
| P15313 | V-type proton ATPase subunit B, kidney isoform | ATP6V1B1 | 3 | 5 | 56,8 | 0,14 | 0,746 | 0,68 | 0,122 |
| P21283 | V-type proton ATPase subunit C 1 | ATP6V1C1 | 1 | 1 | 43,9 | 1,42 | 0,024 | 0,18 | 0,964 |
| Q8NEY4 | V-type proton ATPase subunit C 2 | ATP6V1C2 | 1 | 1 | 48,7 | -0,30 | 0,831 | 0,08 | 0,582 |
| P61421 | V-type proton ATPase subunit d 1 | ATP6V0D1 | 1 | 1 | 40,3 | 0,31 | 0,120 | -0,06 | 0,828 |
| Q9Y5K8 | V-type proton ATPase subunit D | ATP6V1D | 2 | 2 | 28,2 | 0,61 | 0,081 | -0,03 | 0,881 |
| O75348 | V-type proton ATPase subunit G 1 | ATP6V1G1 | 1 | 1 | 13,7 | 0,65 | 0,043 | 0,40 | 0,462 |
| Q9UI12 | V-type proton ATPase subunit H | ATP6V1H | 3 | 3 | 55,8 | 0,23 | 0,248 | 0,03 | 0,726 |
| Q969T9 | WW domain-binding protein 2 | WBP2 | 3 | 3 | 28,1 | 0,24 | 0,385 | 0,37 | 0,292 |
| Q9NQW7 | Xaa-Pro aminopeptidase 1 | XPNPEP1 | 1 | 1 | 69,9 | 0,16 | 0,104 | 0,20 | 0,677 |
| P12955 | Xaa-Pro dipeptidase | PEPD | 1 | 1 | 54,5 | 0,02 | 0,741 | 0,26 | 0,255 |
| Q92834 | X-linked retinitis pigmentosa GTPase regulator | RPGR | 1 | 1 | 113,3 | -0,14 | 0,565 | 0,35 | 0,749 |
| A8MT70 | Zinc finger B-box domain-containing protein 1 | ZBBX | 3 | 3 | 91,4 | 0,13 | 0,162 | 0,35 | 0,188 |
| Q7Z2W4 | Zinc finger CCCH-type antiviral protein 1 | ZC3HAV1 | 7 | 7 | 101,4 | -0,27 | 0,339 | -0,03 | 0,996 |
| P25311 | Zinc-alpha-2-glycoprotein | AZGP1 | 2 | 2 | 34,2 | -0,15 | 0,627 | 0,34 | 0,241 |
| Q96DA0 | Zymogen granule protein 16 homolog B | ZG16B | 1 | 1 | 22,7 | 1,69 | 0,189 | 0,92 | 0,195 |
| *Definition of abbreviations:* FC = fold change. | | | | | | | | | |
